# Ancient collagen reveals evolutionary history of the endemic South American ‘ungulates’

**DOI:** 10.1098/rspb.2014.2671

**Published:** 2015-05-07

**Authors:** Michael Buckley

**Affiliations:** Faculty of Life Sciences, Manchester Institute of Biotechnology, 131 Princess Street, Manchester M1 7DN, UK

**Keywords:** ancient collagen, *Macrauchenia*, *Toxodon*, South American ungulates

## Abstract

Since the late eighteenth century, fossils of bizarre extinct creatures have been described from the Americas, revealing a previously unimagined chapter in the history of mammals. The most bizarre of these are the ‘native’ South American ungulates thought to represent a group of mammals that evolved in relative isolation on South America, but with an uncertain affinity to any particular placental lineage. Many authors have considered them descended from Laurasian ‘condylarths’, which also includes the probable ancestors of perissodactyls and artiodactyls, whereas others have placed them either closer to the uniquely South American xenarthrans (anteaters, armadillos and sloths) or the basal afrotherians (e.g. elephants and hyraxes). These hypotheses have been debated owing to conflicting morphological characteristics and the hitherto inability to retrieve molecular information. Of the ‘native’ South American mammals, only the toxodonts and litopterns persisted until the Late Pleistocene–Early Holocene. Owing to known difficulties in retrieving ancient DNA (aDNA) from specimens from warm climates, this research presents a molecular phylogeny for both *Macrauchenia patachonica* (Litopterna) and *Toxodon platensis* (Notoungulata) recovered using proteomics-based (liquid chromatography–tandem mass spectrometry) sequencing analyses of bone collagen. The results place both taxa in a clade that is monophyletic with the perissodactyls, which today are represented by horses, rhinoceroses and tapirs.

## 1. Introduction

The very concept of extinction was developed from early nineteenth century investigations following the discovery of unusual South American mammal fossils. Charles Darwin himself was one of the first to collect *Toxodon platensis* and *Macrauchenia patachonica* fossils, which are believed to have initiated many debates on evolution and natural selection [1,2]. Despite playing an important part in Darwin's wider studies on evolution, the evolutionary history of the ‘native’ South American megafauna has remained highly debated ever since [3–6].

After the break-up of Pangaea approximately 200 Ma, at around 135 Ma Gondwana began to break up, when western Gondwana (South America and Africa) began to separate from eastern Gondwana (Antarctica, India, Madagascar, Australia and New Zealand) with South America finally separating from West Antarctica approximately 30 Ma with the opening of the Drake Passage [7,8]. It is believed that here in South America, a large variety of unique mammals evolved in relative isolation, including the xenarthrans and the extinct orders of Xenungulata, Notoungulata, Litopterna, Pyrotheria and Astrapotheria, five ancient ‘ungulate’ orders that evolved in isolation from other ‘ungulates’ for at least 60 Myr, until the Panamanian land bridge formed in the Late Pliocene approximately 3 Ma [9]. The notoungulates (e.g. *Toxodon*, *Mesotherium, Homalodotherium*) and litopterns (*Neolicaphrium, Macrauchenia* and *Xenorhinotherium*) were some of the only South American survivors of this Great American Biotic Interchange (GABI) event, with both groups eventually dying out in the Late Pleistocene–Early Holocene. While fossil South American ‘ungulates’ have long been recognized as part of Eutheria [5,10], their relationship to living placentals has yet to be confidently resolved [11,12].

The oldest putative litoptern is *Requisia vidmari* from the ‘Banco Negro Inferior’ of the Salamanca Formation [13], which has recently been dated to the early Palaeocene at 65.7–63.5 Ma [14]. However, there are some doubts as to whether *Requisia* is a litoptern or whether it is a member of a different order (Notopterna [15]). If the latter, the next oldest putative litoptern is *Wainka tshotshe* from the Carodnia Zone, which is currently estimated as Middle Palaeocene in age (61–62 Ma [16]). However, by the Late Palaeocene it is well established that the litopterns had a wide distribution throughout South America, with families including the Protolipternidae [17], Notonychopidae [15], Indalecidae [18], and continued throughout the Early Eocene to middle Miocene (e.g. Adianthidae [19]) and into the Late Pleistocene (Proterotheriidae and Macraucheniidae [20]; the latter forming one focus of this study). Owen first placed *Macrauchenia,* which weighed close to one tonne [21], within the order Perissodactyla, as did many that followed [22,23].

The notoungulates are also known from the Early Palaeocene [6] to the beginning of the Holocene, with the most recent being found in association with human remains [24]. The notoungulates [4] include the families Homalodotheriidae, Leontiniidae, Interatheriidae, Mesotheriidae, Hegetotheriidae and Toxodontidae. Also weighing approximately 1 tonne [21], the Pleistocene toxodonts (*Toxodon* and *Mixotoxodon*) were the size of rhinoceroses and hippopotami and possessed strongly arched upper incisors and molars and horizontally arranged lower incisors. Using the specimen brought back by Darwin, Owen referred the *Toxodon* to the now abandoned Order Pachydermata, which included the elephants, rhinoceroses and hippopotami, but added that they also had ‘affinities to the Rodentia, Edentata, and Herbivorous Cetacea’ (see [23], p.16; [2]).

Given the many morphological features that the South American ungulates share with the true ungulate groups (Artiodactyla and Perissodactyla) of Laurasiatheria, some authors consider that at least some are related to different groups of ‘condylarthrans’, a non-monophyletic assemblage that includes later ‘ungulate’ lineages [25]. It is traditionally considered that of the later Tertiary ungulates, the Artiodactyla is closely related to, or derived from, the ‘condylarth’ families Hyopsodontidae [26,27], or Arctocyonidae [28,29], and that Perissodactyla is derived from the Phenacodontidae [30–32]. The litopterns were initially thought to be derived from the phenacodontid condylarths [33]. Contrary to this, Cifelli [17], based on dental characters, suggested that the litopterns, as well as the didolodontids and possibly notoungulates, were derived from the hyopsodontid ‘condylarths’. Other recent reports have found both Hyopsodontidae and Phenacodontidae to be closer to Perissodactyla [34], while some place Hyopsodontidae and Phenacodontidae outside Euungulata (Cetartiodactyla and Perissodactyla) entirely [35], demonstrating the uncertainty surrounding their precise phylogenetic relationships. An alternative proposal put forward by McKenna [36] placed all of the ‘endemic’ South American fauna, including pyrotheres (previously allied with Proboscidea) and the xenungulates (previously allied with uintatheres) derived from a single radiation, into a grouping that he named ‘Meridiungulata’ that may have originated from a Late Cretaceous ancestor of the endemic Late Palaeocene ‘condylarth’ *Perutherium* [25]. But with increasing molecular sequence-derived phylogenetic reconstructions and the abandonment of the wastebacket taxon ‘Condylarthra’, where several of its ungulate descendants derive from distantly related groups, the placement of the South American ungulates remains uncertain.

Recent molecular evidence indicates that all living placental mammals belong to four major clades of eutherian mammals: Euarchontoglires, Laurasiatheria, Afrotheria and Xenarthra [37,38]. The euarchontoglirans and laurasiatherians are considered to form a well-supported grouping called Boreoeutheria, developed in Laurasia [39], whereas Afrotheria are mostly an African group and Xenarthra are primarily confined to South America.

However, although the monophyletic unity of Xenarthra has been supported by most morphological and molecular studies, the relationship of this group to the other major lineages remains unclear. One hypothesis recognizes the clade Atlantogenata, which comprises the predominantly Gondwana Xenarthra and Afrotheria [40–42]. Following this logic, the South American meridungulates have been considered by some to potentially form part of this Atlantogenata clade [43]. Alternative hypotheses have proposed the combination of Xenarthra and Boreoeutheria (Exafroplacentalia [44]) or the combination of Afrotheria and Boreoeutheria to the exclusion of Xenarthra (Epitheria [45]). One of the most recent analyses of South American ungulate phylogeny considered the notoungulates to be most closely related to mammal groups that are within Afrotheria based on similarities in tooth replacement, the number of thoracic vertebrate and the presence of a well-defined astragalar cotylar fossa [3]. However, others disagree with some of these proposed similarities [46–48].

### (a) Recovering a molecular phylogeny from the fossil record

In recent years, our understanding of mammal evolution has been substantially altered by the analysis of modern DNA [49,50]. However, there are numerous major classes of taxa that are beyond the accepted survival limits of aDNA. These are either owing to the geological age of the most recent representative fossils, or owing to their habitation and geographical location (such as warm or wet environments) and thus fossilization in climates that quickly degrade DNA molecules. Many of these regions are those with the greatest biodiversity, including many of the regions of the former Gondwanan supercontinent (e.g. Africa, Madagascar, South America and Australia) from which aDNA is rarely reported from remains more than a few hundred years old.

Proteins, another phylogenetically informative class of biomolecules, do survive in fossils for periods of time that are orders of magnitude greater than for DNA [51] and have been investigated for their potential to resolve the phylogeny of extinct taxa for decades. Some of the earliest molecular evidence to support the now widely accepted Afrotheria clade came from protein-based evidence [52], although this was not widely accepted until much later DNA sequence analyses. Early studies that used proteins for phylogenetic inferences were most frequently derived from immunological analyses [53–55], where applications of direct sequencing methods to ancient proteins (e.g. [56]) were limited by diagenetic alterations to the proteins (e.g. amino-terminal modifications) and the requirement to isolate and purify large quantities of protein [57]. However, immunological approaches to the study of ancient proteins were considered unreliable by some owing to the regular occurrence of non-specific reactions [58,59].

Recent developments in protein sequence analysis enable complex mixtures of proteins (i.e. proteomes) to be routinely analysed using techniques of ‘soft-ionization’ mass spectrometry. This technology now allows us the ability to obtain protein sequence information and infer evolutionary relationships from long-extinct organisms much deeper into the past than previously thought possible. Although the claims of protein sequence retrieval from dinosaur fossils [60] have proven controversial [61,62], their survival in remains from temperate climes from throughout the Pleistocene period is widely accepted [63,64].

### (b) Collagen structure, survival and phylogenetic potential

Although the biomineralized tissue that is bone contains thousands of different proteins [65], most of these do not survive long periods of time within a burial environment, where a general decrease in proteome complexity with increasing chronological age has been observed [66]. However, the dominant protein of bone, type 1 collagen, has been demonstrated to survive much longer than other non-collagenous proteins (NCPs) [66,67] and also, more importantly, in specimens that no longer yield aDNA [67]. Recent analyses unambiguously reporting the survival of collagen within Pliocene sub-fossil material dating from approximately 3.5 Ma [68] demonstrate its potential for a wide range of extinct taxa.

The exceptional survival of this protein is largely owing to its structure, in which large numbers of triple helical collagen molecules (tropocollagen) are cross-linked into highly stable fibrils and fibres [69,70]. In biomineralized tissues such as bone and dentine—where type 1 collagen is the dominant protein [71,72]—the spaces within and between collagen fibres are filled with mineral hydroxyapatite [73], which could further stabilize the structure, having the side-effect of prolonged survival in the burial environment. The collagen molecule is composed of three chains called alpha chains; type 1 collagen is composed of two highly conserved genetically identical alpha 1 (I) chains, and a third genetically distinct alpha 2 (I) chain. Collagen is typically characterized by a Gly-Xaa-Yaa amino acid sequence motif, where Xaa and Yaa can be almost any amino acid (with the notable exception of Cys within the processed tropocollagen molecule), but frequently a proline (Pro) and hydroxyproline (Hyp) to induce the twist required to create the well-known triple helix structure. However, in the alpha 2 (I) chain this Gly-Pro-Hyp motif is much less frequently adhered to, resulting in a much greater sequence variation relative to the alpha 1 (I) chain. This observation led to the development of proteomics-based methods that use alpha 2 (I) peptides as markers for species identification in fragmentary archaeological [74–77] and palaeontological bone [63,68], but has also proven useful in retrieving phylogenetic information producing topologies consistent with recent DNA-based approaches [78]. This study seeks to use these recently developed methods of collagen sequencing using proteomics methodologies to decipher the evolutionary history of the endemic South American ungulate orders Litopterna and Notoungulata.

## 2. Material and methods

### (a) Collagen screening

To screen a range of sub-fossil specimens (electronic supplementary material S1), collagen extraction was carried out following methods described by Wadsworth & Buckley [66]. In brief, specimens were decalcified with 0.6 M hydrochloric acid (HCl) for approximately 18 h (overnight), and centrifuged at 14 000 r.p.m. for 5 min. The supernatant was then removed and frozen, while the acid-insoluble residue was gelatinized with 6 M Guanidine hydrochloride/5 mM Tris–HCl for a further 18 h. The acid-soluble collagen was then applied to a 10 kDa ultrafilter (Vivaspin, UK) and centrifuged, which was repeated with the centrifuged supernatant from the acid-insoluble residue extraction. Once the solubilized proteins had passed through the ultrafilter, two volumes of ammonium bicarbonate (50 mM; ABC) were also passed through. Once both volumes had filtered through, a further 200 µl ABC were added to the filter, mixed and recovered. This was then incubated with 10 µl 100 mM dithiothreitol (in 50 mM ABC) for 10 min at 60°C. After cooling, 40 µl of iodoacetamide were then added to each sample and then stored in the dark at room temperature for 45 min. A further 10 µl of 100 mM dithiothreitol were added to quench the reaction and the sample was digested overnight with 2 µg sequencing grade trypsin (Promega, UK) at 37°C. The tryptic digests were then cleaned using C18 ziptips following the manufacturer's procotol (Varian OMIX, UK), dried down and resuspended with 10 µl 0.1% trifluoroacetic acid; 1 µl was then spotted onto a Bruker 384 well Matrix Assisted Laser Desorption Ionization (MALDI) target plate and co-crystalized with 1 µl alpha-cyano hydroxycinnamic acid prior to MALDI analysis. MALDI spectra representing peptide mass fingerprints (PMFs) were acquired on a Bruker Ultraflex II with a time of flight (ToF) mass analyser, over an *m/z* range of 700–3700 using up to 2000 laser acquisitions.

### (b) Collagen sequencing by liquid chromatography–tandem mass spectrometry

Digested samples were analysed by liquid chromatography–tandem mass spectrometry (LC–MS/MS) using an UltiMate 3000 Rapid Separation LC (RSLC, Dionex Corporation, Sunnyvale, CA, USA) coupled to an Orbitrap Elite (Thermo Fisher Scientific, Waltham, MA, USA) mass spectrometer (120k resolution, Full Scan, Positive mode, normal mass range 350–1500). Peptides in the sample were separated on a 75 mm × 250 µm i.d. 1.7 μM Ethylene Bridged Hybrid (BEH) C18 analytical column (Waters, UK) using a gradient from 92% A (0.1% formic acid in water) and 8% B (0.1% formic acid in acetonitrile) to 33% B in 44 min at a flow rate of 300 nl min^−1^. Peptides were then automatically selected for fragmentation by data-dependent analysis; 6 MS/MS scans (Velos ion trap, product ion scans, rapid scan rate, Centroid data; scan event: 500 count minimum signal threshold, top 6) were acquired per cycle, dynamic exclusion was employed and one repeat scan (2 MS/MS scans total) was acquired in a 30 s repeat duration with that precursor being excluded for the subsequent 30 s (activation: CID, 2 + default charge state, 2 *m*/*z* isolation width, 35 eV normalized collision energy, 0.25 Activation Q, 10.0 ms activation time). Peptide spectra obtained via LC–MS/MS were searched against the SwissProt database using the Mascot search engine (v. 2.2.0.6; Matrix Science, London, UK). Error-tolerant searches included the fixed carbamidomethyl modification of cysteine (+57.02 Da) and the variable modifications for oxidation of lysine and proline residues (all +15.99 Da) to account for post-translational modifications (the oxidation of lysine and proline being equivalent to hydroxylation commonly observed in collagen, the dominant protein in bone), whereas decoy searches were run with the additional variable modifications allowing for the oxidation of methionine and deamidation of asparagine and glutamine (+0.98 Da) to allow for diagenetic alterations. Enzyme specificity was limited to trypsin (trypsin/P) with one (error-tolerant) or two (decoy) missed cleavages allowed, mass tolerances were set at 5 ppm for the precursor ions and 0.5 Da for the fragment ions; all spectra were considered as having either 2+ or 3+ precursors. Highest matching peptide scores for homologous sequences were then manually inspected for quality (e.g. electronic supplementary material S2) and the most appropriate added to a custom sequence database (electronic supplementary material S3) for subsequent further error-tolerant and decoy Mascot searches.

### (c) Data analysis

The Mascot results from the MS/MS queries (totalling 50,842 for the four specimens that produced collagen PMFs; electronic supplementary material S4) were filtered to only include peptide matches greater than the highest false-positive peptide match score for that individual analysis (the highest false-positive peptide matches identified in the decoy searches ranged from 26 to 32 for the four samples against the custom concatenated type 1 collagen database); only peptide matches found in both specimens for each species were used for the sequence analyses. For increased confidence in the sequences obtained, several modifications to the dataset were made: (i) one set of analyses was carried out using peptide matches in both of each taxa (i.e. both *Macrauchenia* or both *Toxodon* analyses) and (ii) an alternative approach was to only include peptide sequences where their precursor masses had been observed in the PMF for that species. As almost all of the peaks observed in the PMFs of such total bone extract represent type 1 collagen peptides [79], this approach is intended to account for any issues with the LC–MS/MS data that may arise owing to either contamination (for which current LC-MS/MS instrumentation is much more sensitive) or to potential matches from other collagen types from taxa with known genome information resulting from paralogy [79]. A third variation was also tested in which all sequence information homologous to collagen sequence not observed in either *Macrauchenia* or *Toxodon* was removed in a ‘pseudoextinction’ approach [80]. These sequences were then ordered by position and manually aligned in BioEdit Sequence Alignment Editor v. 7.1.3.0 with X representing unknown/unmatched amino acid residues (? when at an indel site in sequences from other taxa). Phylogenetic analyses of the concatenated collagen alpha 1 and alpha 2 sequences (via an R residue; yielding a total length of 2099 amino acid residues) were then carried out using the PhyML plugin [81] for Geneious v. 7.1.2 with 44 other mammalian type 1 collagen sequences (concatenated chains) obtained from the Ensembl databases and the UCSC genome browser (the most abundant of each isobaric leucine/isoleucine residue used throughout). The JTT + I + G model was used, identified as most appropriate by PartitionFinderProtein v. 1.1.1 [82]. Trees were rooted to the duck-billed platypus (*Ornithorhynchus*) as a prototherian out-group. Nearest neighbour interchange (NNI) branch swapping was used with 10 000 bootstrap replicates carried out to estimate support (see electronic supplementary material S5 for best of both SPR and NNI analysis). Parsimony analyses were carried out using Phylip v. 3.695 and also rooted to the duck-billed platypus. Bayesian analyses were also carried out using the MrBayes v. 3.2.2 [83] with 2 500 000 MCMC generations (see electronic supplementary material S5 for trace analysis), discarding the first 25% as burn-in, estimated invariable gamma distribution (four categories), four chains (three heated, one cold) with unconstrained branch lengths and also rooted to the duck-billed platypus.

## 3. Results

**Figure 1.**
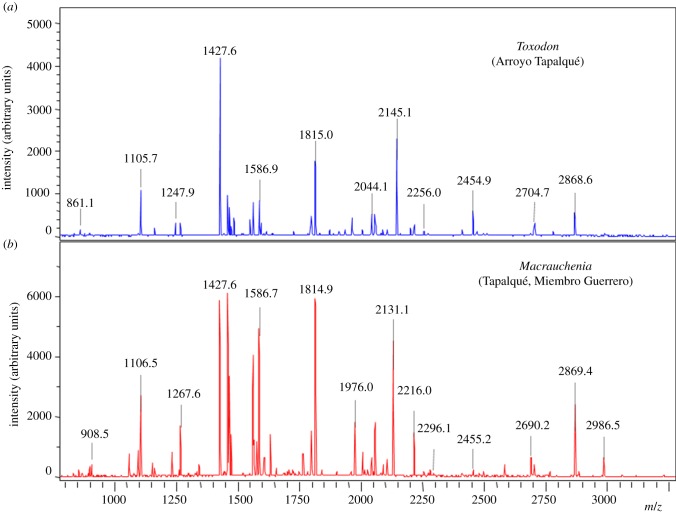
MALDI-ToF mass spectra of collagen extracted from *Toxodon* (*a*) and *Macrauchenia* (*b*).

**Figure 2.**
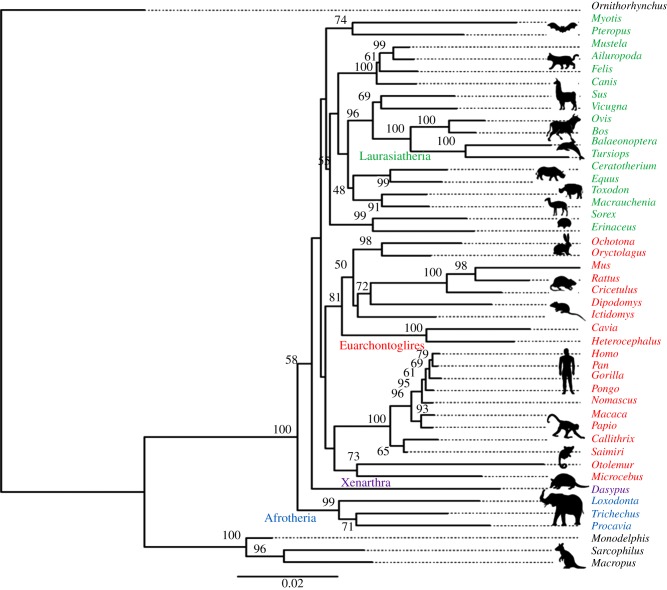
Phylogenetic analyses of *Toxodon* and *Macrauchenia* collagen sequences matched by LC–MS/MS rooted to the duck-billed platypus (*Ornithorhynchus*) showing maximum-likelihood analysis using PhyML with 10 000 bootstraps (less than 50 not shown except for *Toxodon* and *Macrauchenia*).

Collagen was successfully extracted from two *Macrauchenia patachonica* and two *Toxodon platensis* sub-fossil specimens from different palaeontological sites in Buenos Aires, Argentina. Prior to LC-OrbitrapElite-MS/MS analyses, MALDI-ToF-MS PMFs were acquired to evaluate the quality of the surviving proteins extracted and digested (figure 1). LC–MS/MS data were then searched against SwissProt by Mascot using error-tolerant searches to allow for further amino acid substitutions between species, which were manually investigated, and greater scoring matches from other species co-opted to improve the existing collagen sequence for each specimen (collagen was the only protein matched in all four samples). Molecular phylogenies were then reconstructed following further Mascot search results with varying levels of confidence using maximum-likelihood (ML) analyses, all recovering a similar placement for the South American ungulates (figure 2; electronic supplementary material S5: figures S2 and S3). To explore other phylogenetic methods, parsimony (electronic supplementary material S5: figure S4) and Bayesian (electronic supplementary material, figure S5) analyses were also carried out on the sequences matched in both specimens for each sub-fossil taxon; both yielded relationships of the South American ungulates within Laurasiatheria, monophyletic with Perissodactyla.

Owing to the limitations of sequence coverage for these sub-fossil collagens (table 1), a pseudoextinction approach to testing the phylogenetic signal within the dataset was also tested using ML whereby all sequences homologous to missing data from either *Toxodon* or *Macrauchenia* were excluded throughout the dataset [80], resulting in 1460 total characters. The resulting placement of the South American ungulates remained consistently within a clade that was monophyletic with Perissodactyla, although some differences were observed elsewhere in the topology where the primates no longer grouped with the monophyletic rodents and lagomorphs, and the xenarthran was placed sister to the chiropterans (electronic supplementary material, figure S2). An alternative approach to using proteomics-derived data but to minimize the inclusion of false-positive matches is also proposed here, which only takes into account peptides that were observed in the PMF experiments. The results from this analysis recovered the same tree topology as described above for the standard analyses (i.e. consistent overall topology with [37]) but with subtly lower bootstrap support for the association of the South American ungulates with Perissodactyla.

**Table 1.** Decoy rates and percentage sequence coverages (of 2097 collagen residues) of peptide matches above the highest false-positive peptide score for the sequence data of the four South American native ungulate specimens analysed as well as protein scores and number of peptide matches (number of unique peptides in brackets) for selected representative taxa (cropped to exclude sequence gaps; see electronic supplementary material S4 for further peptide score information). MO, Museo Olavarría; MLP, Museo de La Plata.

Macrauchenia				Toxodon			
MLP 71-III-6-1 (2.04/71% > 27)		MO (1.77/72% > 26)		MLP 81-II-5-7 (1.89/56% > 30)		MLP 86-III-25-15 (1.60/70% > 32)	
*Oryctolagus*	10 465 (97/74)	*Canis*	22 595 (78/60)	*Sus*	7583 (82/58)	*Canis*	14 035 (101/71)
*Sus*	10 244 (89/65)	*Equus*	22 509 (101/73)	*Sorex*	6970 (78/52)	*Sus*	13 778 (111/78)
*Equus*	10 159 (97/70)	*Sus*	22 079 (90/64)	*Canis*	6737 (73/55)	*Ceratotherium*	13 434 (107/76)
*Ceratotherium*	9929 (84/61)	*Ceratotherium*	21 421 (85/61)	*Ceratotherium*	6633 (69/53)	*Bos*	13 400 (109/78)
*Canis*	9925 (93/64)	*Oryctolagus*	20 257 (97/69)	*Bos*	6565 (79/59)	*Sorex*	13 214 (104/71)
*Sorex*	9231 (82/59)	*Bos*	20 135 (80/62)	*Equus*	6417 (76/52)	*Equus*	13 077 (113/78)
*Bos*	9193 (92/67)	*Sorex*	20 109 (93/60)	*Oryctolagus*	6202 (74/52)	*Oryctolagus*	12 906 (105/76)
*Gorilla*	9129 (97/68)	*Gorilla*	17 579 (93/64)	*Gorilla*	5720 (84/55)	*Gorilla*	12 054 (110/72)
*Loxodonta*	8218 (87/66)	*Loxodonta*	17 232 (84/61)	*Myotis*	5448 (73/46)	*Myotis*	11 443 (95/60)
*Dasypus*	7785 (87/53)	*Mus*	15 361 (69/44)	*Dasypus*	5358 (63/44)	*Loxoodonta*	10 504 (104/69)
*Myotis*	7575 (77/54)	*Myotis*	22 573 (118/76)	*Mus*	5286 (47/38)	*Mus*	9970 (83/56)
*Mus*	7015 (70/43)	*Dasypus*	14 732 (85/53)	*Loxodonta*	5178 (73/51)	*Dasypus*	9884 (95/60)

The phylogenetic results from the available collagen sequences clearly demonstrate its potential to retrieve topologies consistent with those recently reported for molecular analyses of extant taxa [37]. According to the analyses that are most consistent with DNA-based methods (ML), the afrotherians are the first placental superorder to diverge, with xenarthrans placed as sister clade to the remaining taxa, consistent with the Exoafroplacentalia model of eutherian evolution. Boreoeutheria is recovered with Euarchonta and Glires forming a clade (Euarchontoglires) sister to Laurasiatheria. In the latter, the Chiroptera are the first order to diverge within Laurasiatheria, followed by Eulipotyphla (albeit both with poor support), then by Carnivora and finally the Perissodactyla and Cetartiodactyla; the latter three forming Fereuungulata. Within this phylogeny both *Toxodon* and *Macrauchenia*, which were found to be monophyletic (Meridungulata), were consistently placed as sister taxa to Perissodactyla within Laurasiatheria. Although there was only one synapomorphy identified for this proposed grouping, there were several between the South American native ungulates and either *Ceratotherium* or *Equus;* however, a similar trend in low numbers of group synapomorphies is observed in other lineages such as the cetartiodactyls, which form a strongly supported clade.

## 4. Discussion

Following many decades of debate hitherto based purely on skeletal morphology, the molecular phylogeny retrieved from collagen sequencing consistently resolves the evolutionary history of these morphologically unusual South American native ‘ungulates’. Their placement as sister group to the perissodactyls indicates that the litopterns and notoungulates, and potentially others of the ‘native’ South American mammals, derive from a lineage nested within the Euungulata (Perissodactyla and Cetartiodactyla, with estimated divergence times of approx. 75 Ma [84,85]), well within the ‘northern mammal’ superordinal clade Boroeutheria and so refuting their placement within Afrotheria (also supported by the relatively low protein scores for *Loxodonta* shown in table 1) as suggested by Agnolin & Chimento [3].

Given that the fossil record of Tertiary South American ‘ungulates’ starts in the earliest Palaeocene age, with the condylarths and a notoungulate from the Santa Lucia Formation at Tiupampa, Bolivia [6], and condylarths and litopterns from Punta Peligro, in Patagonia, Argentina [13], it is likely that the colonization of South America by the Meridungulate ancestor(s) occurred from North America during the earliest Palaeocene, or even the Late Cretaceous when the two continents were probably only separated by a narrow water gap, but later became more widely separated [86]. The findings of litopterns (Sparnotheriodontidae) and astrapotherians (Trigonostylopoidea) occurring in Eocene deposits of Seymour Island, along with marsupials, xenarthrans and gondwanatherians [87], have been interpreted as an indication that Antarctica and South America would have had a land connection in the Late Palaeocene–Early Eocene, when major regressive events are recorded in northern Antarctica and southernmost Patagonia [88]. However, given that the group managed to cross the gap from North to South America, it is entirely plausible that they similarly crossed the gap between Patagonia and Antarctica.

The results presented here that identify the Meridungulata as ‘northern mammals’ are consistent with a recent study by Muizon & Cifelli [6] that found dental evidence of affinities among litopterns, didolodontids and mioclaenids (including both North and South American groups). They had designated this group as a new order of mammals, the ‘Panameriungulata’, but the relationships of these groups to extant taxa remained unclear. The findings that the South American ungulates are not placed within a clade representing Atlantogenata is also consistent with evidence from the palaeontological record [89] and some molecular clock estimates [90,91] in that the relevant tectonic events are too old (older than 100 Ma) to be the causal factor behind intra-placental divergences [4]. In a recent study that carried out phylogenetic analyses of eutherian mammals combining morphological and molecular data [48], namely, the South American ‘ungulates’ (the litoptern *Diadiaphorus*, the astrapothere *Trigonostylops*, the notoungulates *Henricosbornia*, *Simpsonotus* and *Thomashuxleya*, and the pyrothere *Pyrotherium*) and the ‘condylarths’ (*Didolodus* and *Paulacoutoia*) were consistently placed in a clade with the fossil perissodactyl *Hyracotherium*, congruent with the current paper's molecular results.

Given the findings of this research placing *Macrauchenia* and *Toxodon* with perissodactyls, it would be of interest to further investigate other Late Pleistocene survivors of these groups. For example, the analysis of *Neolicaphrium*, which is a member of the only other litoptern family to survive the GABI [20], the Proterotheriidae could further resolve the relationships within this group. Likewise of the notoungulates, *Mixotoxodon* has been found further north in central South America [24] and even southern North America [92]. Future analyses of freshly recovered and better preserved material may be able to retrieve further sequence information from NCPs that could yield greater taxonomic resolution [93]. However, making use of the developing field of proteomics to obtain phylogenetically informative sequence information clearly has wide potential application, whereby sequence analysis of this kind could restructure the systematics of a large number of such groups as well as many other questionable placements of fossil taxa beyond the scope of aDNA sequence retrieval worldwide. In this example, this research has provided critical new clues to the origins of these enigmatic taxa that have been at the centre of one of the longest-standing debates in mammalian palaeontology as the first description of litopterns and notoungulates on the basis of bones collected by Darwin in 1834.

## Supplementary Material

ESM1

## Supplementary Material

ESM2

## Supplementary Material

ESM3

## Supplementary Material

ESM4

## Supplementary Material

ESM5

## Data accessibility

Data presented in this paper are available as an electronic supplementary material in five parts and proteomics data and raw tree files accessible through Dryad: doi:10.5061/dryad.9tt2t.

(1) List of specimens sampled and tested for collagen preservation using MALDI-ToF-MS,
(2) additional MALDI spectra and supplementary LC-MS/MS spectra,
(3) sequences and original accession numbers,
(4) mascot peptide ion scores for the four collagen-yielding samples and
(5) additional phylogenetic trees.
