# Supplementary material for: Ancient collagen reveals evolutionary history of the endemic South American ‘ungulates’
Source: Proc Biol Sci. 2015 May 7;282(1806):20142671. doi: 10.1098/rspb.2014.2671 (PMC4426609; doi:10.1098/rspb.2014.2671)
Supplement: ESM2 [file rspb20142671supp2.pdf]

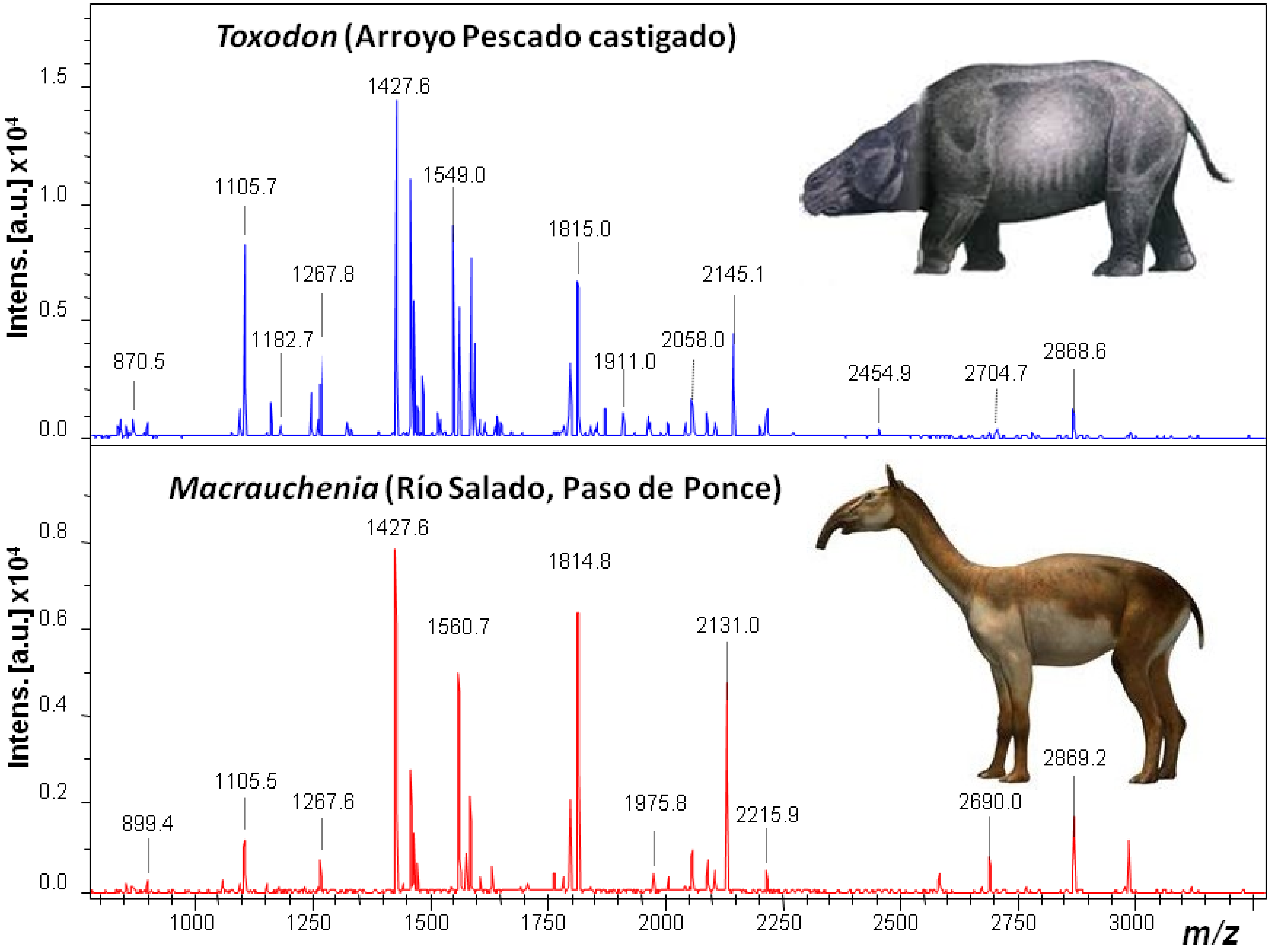
Toxodon (Arroyo Pescado castigado)
Intens. [a.u.] x10⁴
1.5
1.0
0.5
0.0
1427.6
1105.7
1549.0
1815.0
1267.8
2145.1
1182.7
2058.0
870.5
1911.0
2868.6
2454.9
2704.7
Macrauchenia (Río Salado, Paso de Ponce)
Intens. [a.u.] x10⁴
0.8
0.6
0.4
0.2
0.0
1427.6
1814.8
1560.7
2131.0
2869.2
1105.5
1267.6
1975.8
2215.9
2690.0
899.4
1000
1250
1500
1750
2000
2250
2500
2750
3000
m/z

GFPGSDGIAGPK

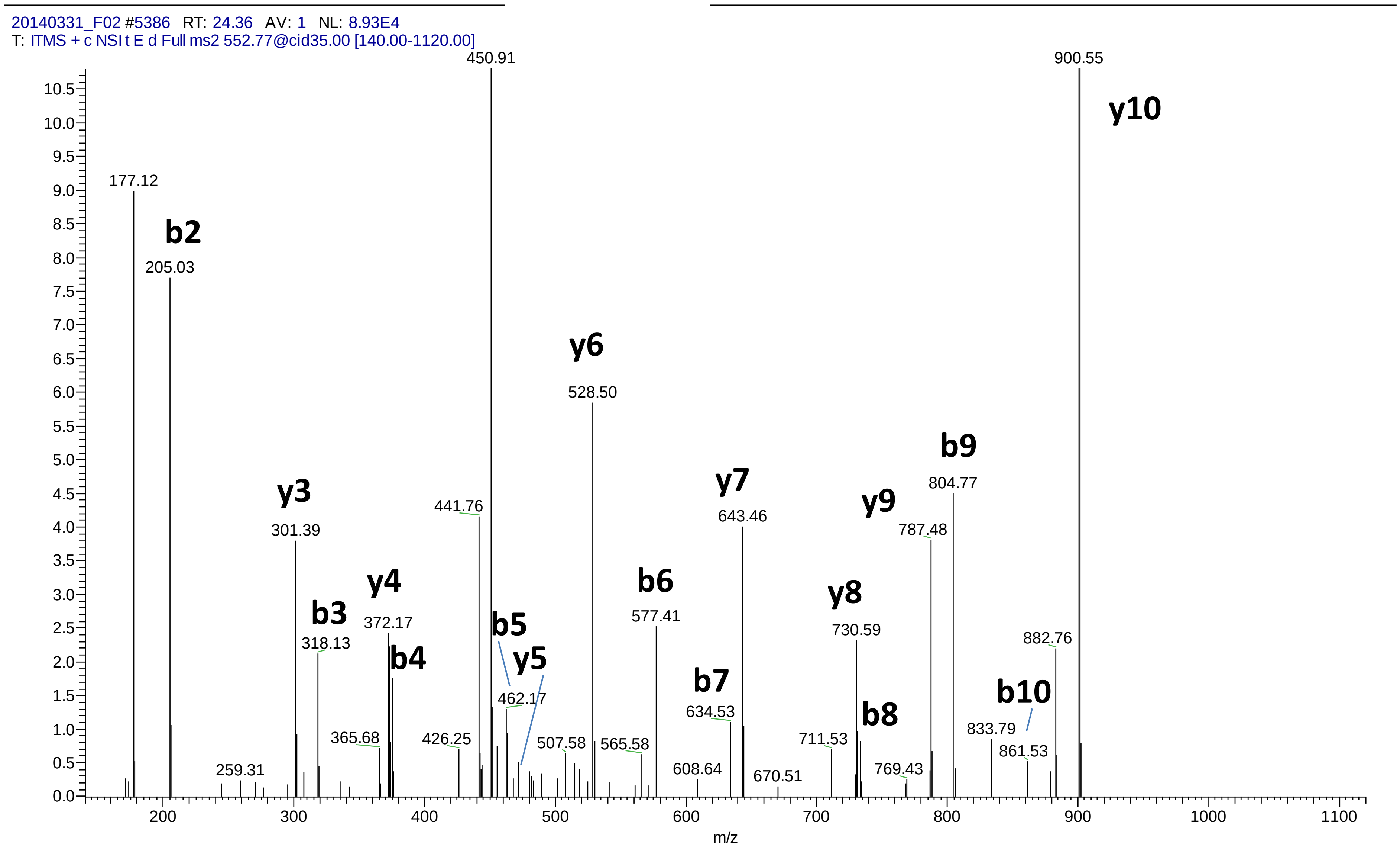

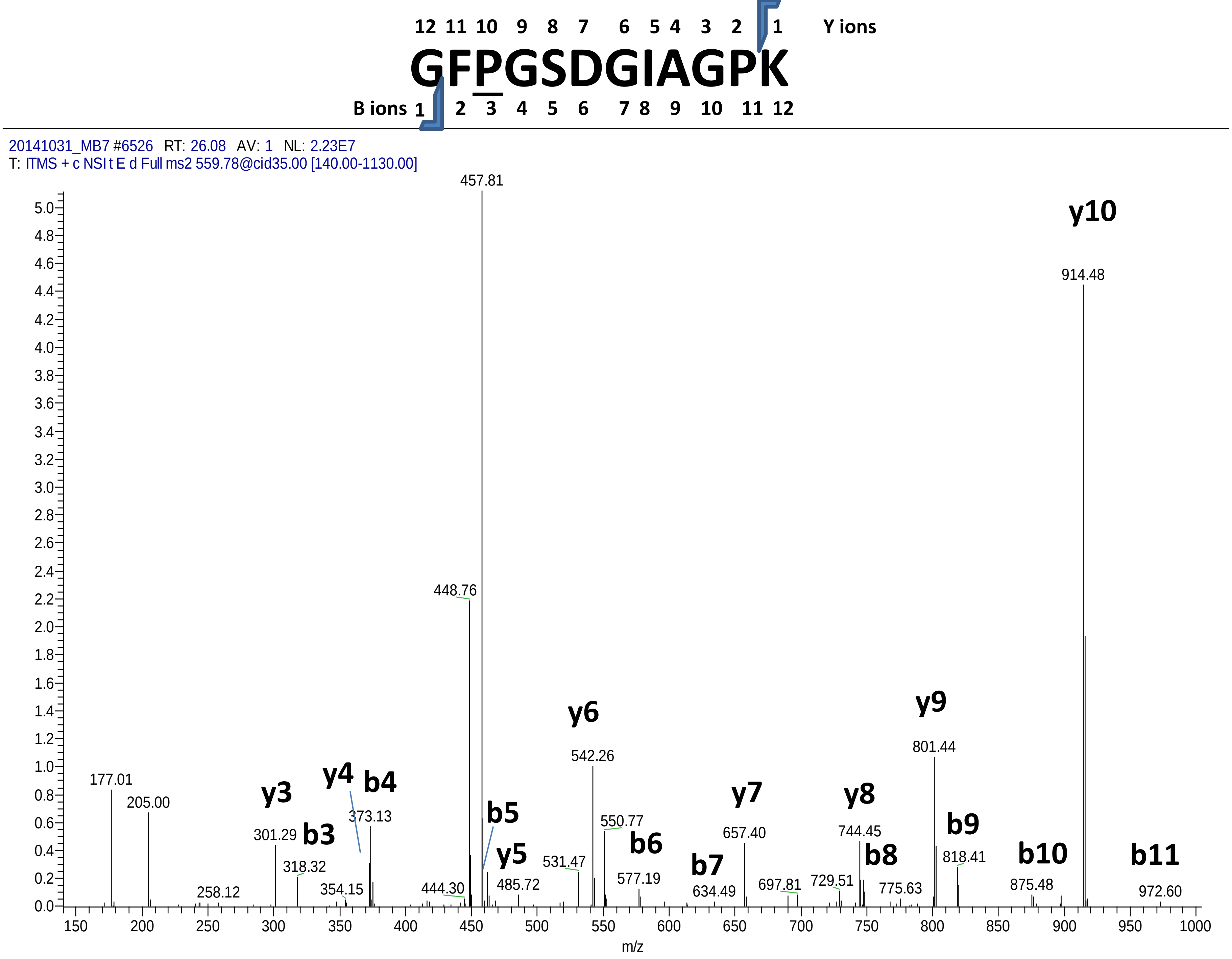
12 11 10 9 8 7 6 5 4 3 2 1 Y ions
GFPGSDGIAGPK
B ions 1 2 3 4 5 6 7 8 9 10 11 12
20141031_MB7 #6526 RT: 26.08 AV: 1 NL: 2.23E7
T: ITMS + c NSI t E d Full ms2 559.78@cid35.00 [140.00-1130.00]
457.81
914.48
448.76
542.26
801.44
177.01
205.00
301.29
318.32
373.13
354.15
258.12
444.30
485.72
531.47
550.77
577.19
634.49
657.40
697.81
729.51
744.45
775.63
818.41
875.48
972.60
y10
y9
y8
y7
y6
y5
y4
y3
b3
b4
b5
b6
b7
b8
b9
b10
b11
m/z

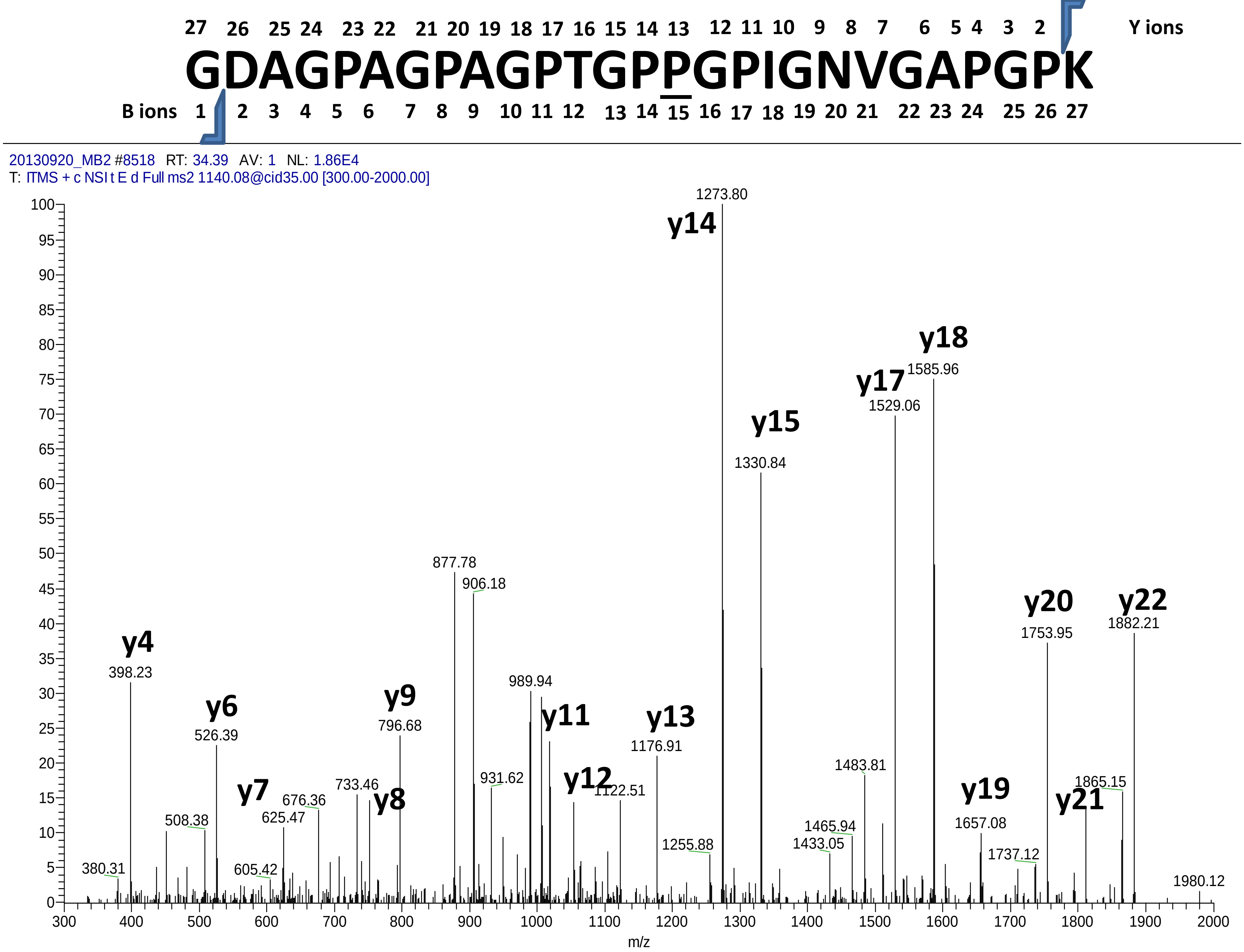
27 26 25 24 23 22 21 20 19 18 17 16 15 14 13 12 11 10 9 8 7 6 5 4 3 2
Y ions
GDAGPAGPAGPTGPPGPIGNVGAPGPK
B ions 1 2 3 4 5 6 7 8 9 10 11 12 13 14 15 16 17 18 19 20 21 22 23 24 25 26 27
20130920_MB2 #8518 RT: 34.39 AV: 1 NL: 1.86E4
T: ITMS + c NSI t E d Full ms2 1140.08@cid35.00 [300.00-2000.00]
y4
398.23
380.31
508.38
y6
526.39
y7
605.42
625.47
676.36
733.46
y8
y9
796.68
877.78
906.18
931.62
989.94
y11
y12
1122.51
y13
1176.91
1255.88
y14
1273.80
y15
1330.84
1433.05
1465.94
1483.81
y17
1529.06
y18
1585.96
y19
1657.08
1737.12
y20
1753.95
y21
1865.15
y22
1882.21
1980.12
m/z

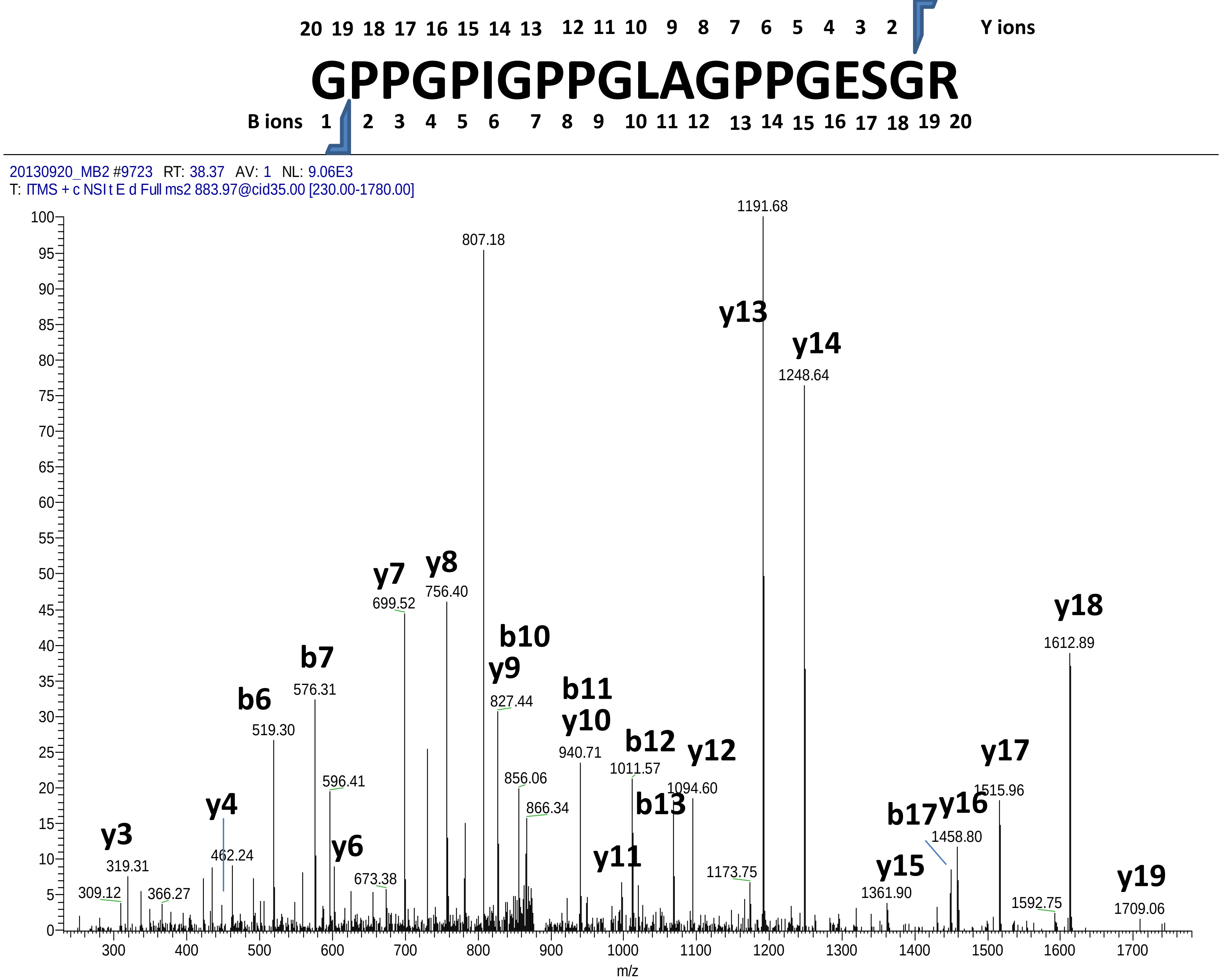
20 19 18 17 16 15 14 13 12 11 10 9 8 7 6 5 4 3 2 Y ions
GPPGPIGPPGLAGPPGESGR
B ions 1 2 3 4 5 6 7 8 9 10 11 12 13 14 15 16 17 18 19 20
20130920_MB2 #9723 RT: 38.37 AV: 1 NL: 9.06E3
T: ITMS + c NSI t E d Full ms2 883.97@cid35.00 [230.00-1780.00]
1191.68
807.18
y13
y14
1248.64
y7
y8
699.52
756.40
b10
y9
b7
576.31
827.44
b11
y10
b6
519.30
940.71
b12
y12
1011.57
596.41
856.06
1094.60
b13
y4
866.34
y3
y6
462.24
319.31
673.38
y11
1173.75
309.12
366.27
y18
1612.89
y17
1515.96
b17
y16
1458.80
y15
1361.90
1592.75
y19
1709.06
m/z

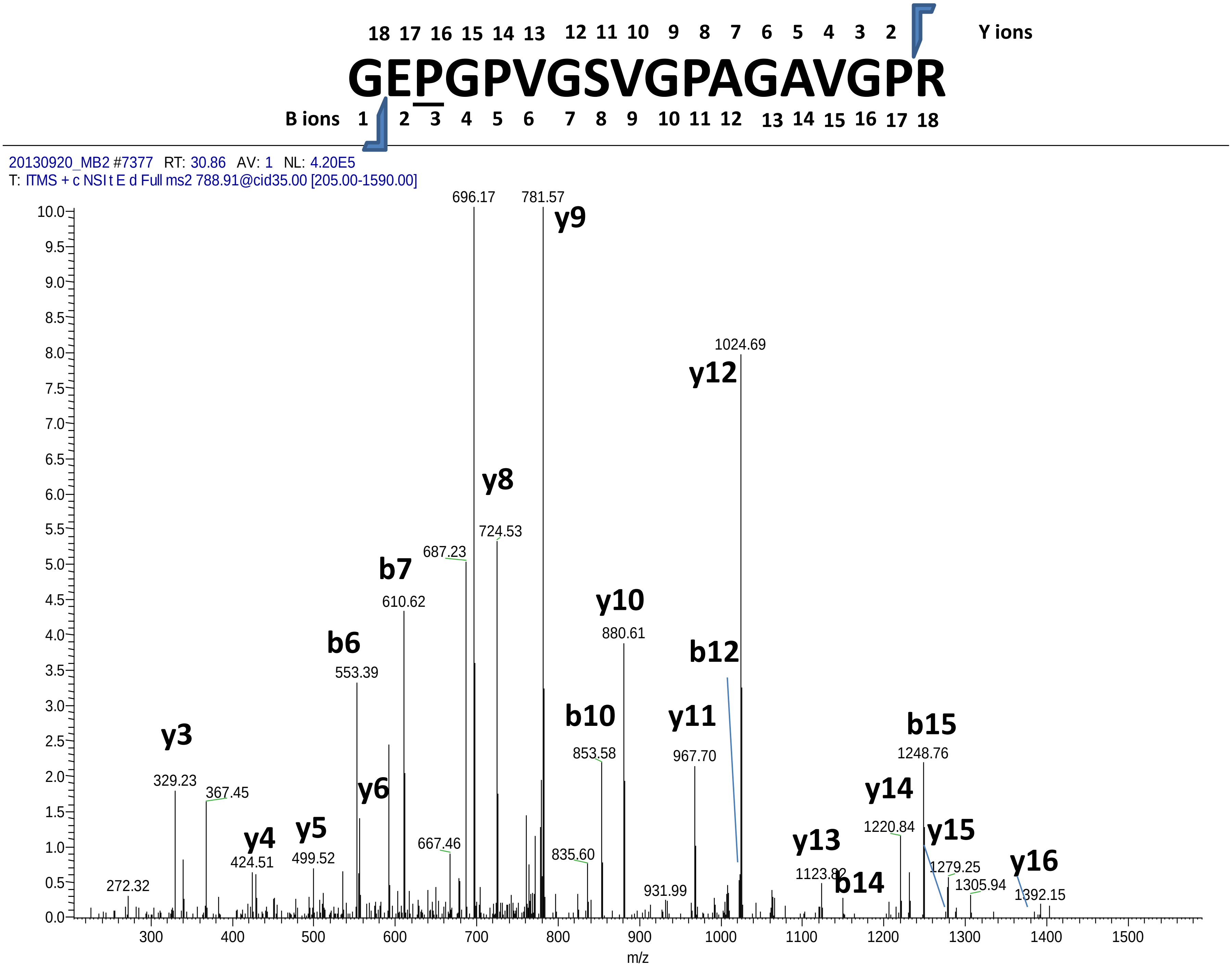

18 17 16 15 14 13 12 11 10 9 8 7 6 5 4 3 2
Y ions
GEPGPVGSVGPAGAVGPR
B ions 1 2 3 4 5 6 7 8 9 10 11 12 13 14 15 16 17 18
20130920_MB2 #7377 RT: 30.86 AV: 1 NL: 4.20E5
T: ITMS + c NSI t E d Full ms2 788.91@cid35.00 [205.00-1590.00]
696.17
781.57
y9
1024.69
y12
y8
724.53
687.23
b7
610.62
y10
880.61
b6
553.39
b12
b10
y11
b15
y3
853.58
967.70
1248.76
329.23
367.45
y6
y14
y5
y4
1220.84
y15
667.46
y13
424.51
499.52
835.60
y16
1279.25
1123.82
b14
1305.94
272.32
931.99
1392.15
10.0
9.5
9.0
8.5
8.0
7.5
7.0
6.5
6.0
5.5
5.0
4.5
4.0
3.5
3.0
2.5
2.0
1.5
1.0
0.5
0.0
300
400
500
600
700
800
900
1000
1100
1200
1300
1400
1500
m/z

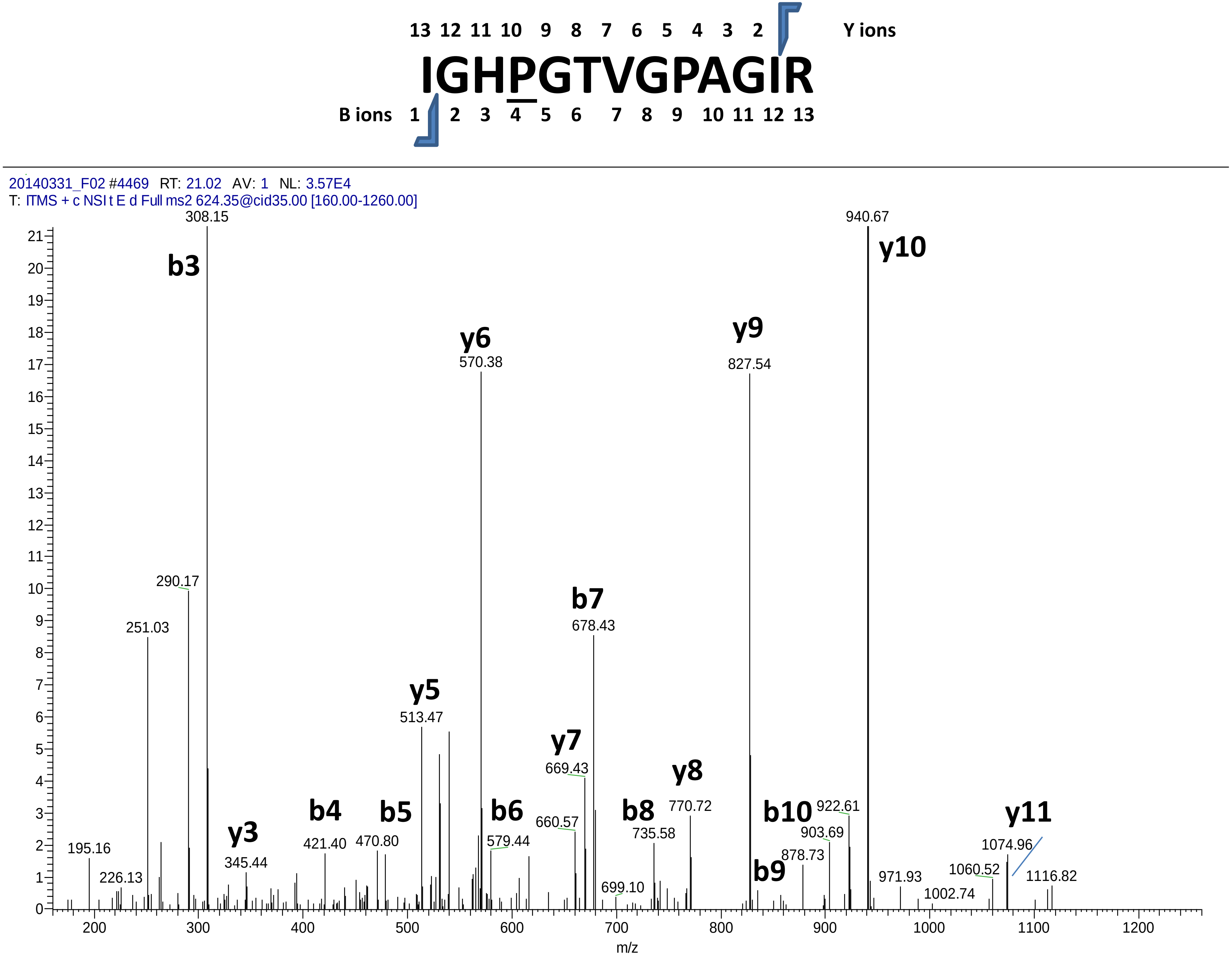

13 12 11 10 9 8 7 6 5 4 3 2
Y ions
IGHPGTVGPAGIR
B ions 1 2 3 4 5 6 7 8 9 10 11 12 13
20140331_F02 #4469 RT: 21.02 AV: 1 NL: 3.57E4
T: ITMS + c NSI t E d Full ms2 624.35@cid35.00 [160.00-1260.00]
308.15
b3
940.67
y10
y6
570.38
y9
827.54
290.17
251.03
b7
678.43
y5
513.47
y7
669.43
y8
770.72
b4
b5
b6
b8
b10
922.61
y11
y3
345.44
421.40
470.80
579.44
660.57
735.58
903.69
878.73
b9
1074.96
195.16
226.13
699.10
971.93
1060.52
1116.82
1002.74
m/z

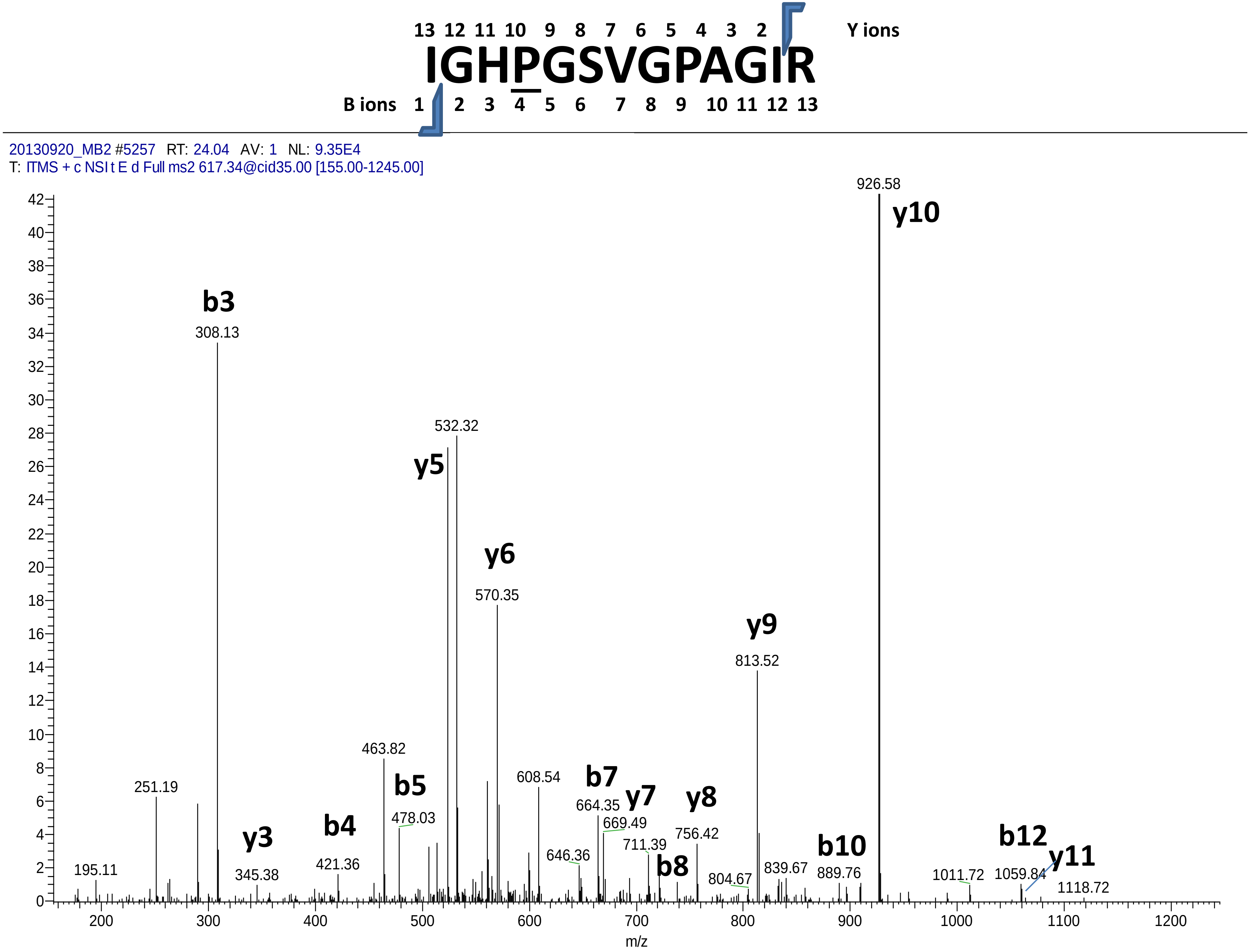

13 12 11 10 9 8 7 6 5 4 3 2
Y ions
IGHPGSVGPAGIR
B ions 1 2 3 4 5 6 7 8 9 10 11 12 13
20130920_MB2 #5257 RT: 24.04 AV: 1 NL: 9.35E4
T: ITMS + c NSI t E d Full ms2 617.34@cid35.00 [155.00-1245.00]
926.58
y10
b3
308.13
532.32
y5
y6
570.35
y9
813.52
463.82
b5
251.19
608.54
b7
664.35
y7
669.49
y8
756.42
478.03
y3
b4
711.39
421.36
646.36
b8
b10
b12
y11
195.11
345.38
804.67
839.67
889.76
1011.72
1059.84
1118.72
m/z

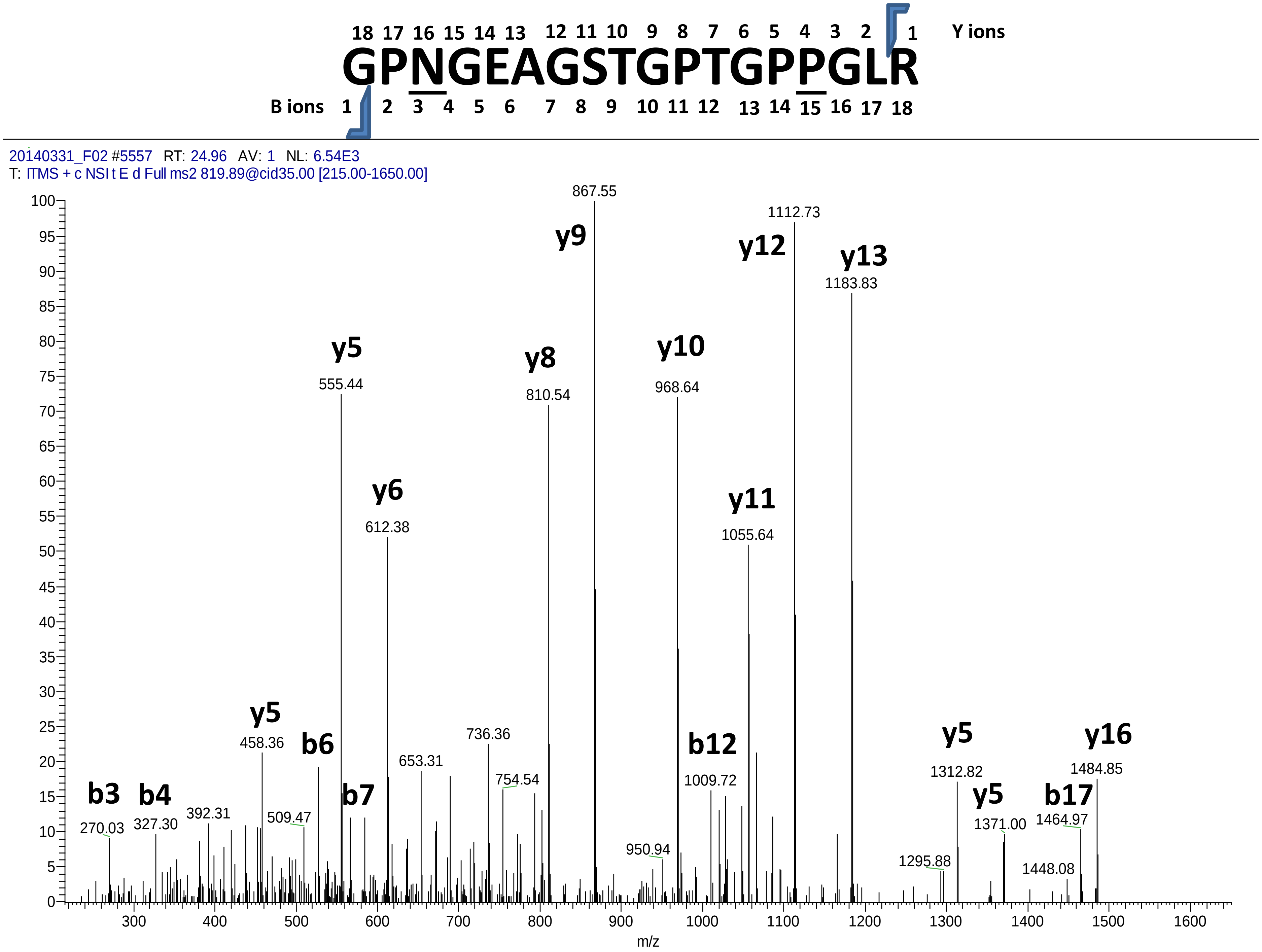

18 17 16 15 14 13 12 11 10 9 8 7 6 5 4 3 2 1 Y ions
GPNGEAGSTGPTGPPGLR
B ions 1 2 3 4 5 6 7 8 9 10 11 12 13 14 15 16 17 18
20140331_F02 #5557 RT: 24.96 AV: 1 NL: 6.54E3
T: ITMS + c NSI t E d Full ms2 819.89@cid35.00 [215.00-1650.00]
867.55
y9
1112.73
y12
y13
1183.83
y5
555.44
y8
810.54
y10
968.64
y6
612.38
y11
1055.64
y5
458.36
b6
736.36
653.31
b12
1009.72
y5
1312.82
y16
1484.85
b3
b4
b7
754.54
270.03
327.30
392.31
509.47
950.94
y5
1371.00
b17
1464.97
1295.88
1448.08
m/z
