# Supplementary material for: Ancient collagen reveals evolutionary history of the endemic South American ‘ungulates’
Source: Proc Biol Sci. 2015 May 7;282(1806):20142671. doi: 10.1098/rspb.2014.2671 (PMC4426609; doi:10.1098/rspb.2014.2671)
Supplement: ESM3 [file rspb20142671supp3.docx]

The sequences were initially acquired from UniProt (6 digit accession labels) or the Ensembl Genome Browser (accession labels starting with ‘ENS’). Many sequences from the latter were checked through BLAT searches to the appropriate genome (Kent 2002). Sequences from species not present in either UniProt or Ensembl were obtained from the UCSC genome browser (Kent et al. 2002). Amino acids from regions considered to be erroneous, i.e., due to lengths of non-helical sequence (lacking the highly conserved glycine residue every three positions) or the presence of cysteine were replaced with an X.

**UCSC Genome Browser:** Kent WJ, Sugnet CW, Furey TS, Roskin KM, Pringle TH, Zahler AM, Haussler D. [The human genome browser at UCSC](http://www.genome.org/cgi/content/abstract/12/6/996). Genome Res. 2002 Jun;12(6):996-1006.

**BLAT:** Kent WJ. [BLAT - the BLAST-like alignment tool](http://www.genome.org/cgi/content/abstract/12/4/656). Genome Res. 2002 Apr;12(4):656-64.

**Accession Information:**

>Equus_F6VUP8_F6RTL6

>Ceratotherium_UCSC_UCSC

>Vicugna_UCSC_ENSVPAT00000002274

>Tursiops_ENSTTRT00000005496_ENSTTRT00000003720

>Balaeonoptera_UCSC_UCSC

>Bos_P02453_P02465

>Ovis_ENSOART00000005320_ENSOART00000001624

>Sus_ENSSSCT00000019139_ENSSSCT00000016699

>Canis_Q9XSJ7_O46392

>Ailuropoda_G1M024_G1MH95

>Felis_ENSFCAT00000004567_ENSFCAT00000027973

>Ornithorhynchus_ENSOANT00000002769_ENSOANT00000003999

>Pongo_ENSPPYT00000010431_ENSPPYT00000020749

>Gorilla_ENSGGOT00000013270_ENSGGOT00000006068

>Saimiri_UCSC_UCSC

>Cricetulus_UCSC_UCSC

>Heterocephalus_UCSC_UCSC

>Papio_ENSPANT00000019135_ENSPANT00000026844

>Microcebus_ENSMICT00000007261_ENSMICT00000008520

>Dipodomys_ENSDORT00000013505_ENSDORT00000003027

>Cavia_ENSCPOT00000013727_ENSCPOT00000010743

>Oryctolagus_ENSOCUT00000012892_ENSOCUT00000012284

>Ictidomys_ENSSTOT00000002009_ENSSTOT00000010817

>Macropus_ENSMEUT00000008452_ENSMEUT00000000935

>Procavia_ENSPCAT00000012263_ENSPCAT00000002825

>Erinaceus_ENSEEUT00000007941_ENSEEUT00000011128

>Sorex_UCSC_ENSSART00000003076

>Trichechus_UCSC_UCSC

>Dasypus_ENSDNOT00000018275_ENSDNOT00000001925

>Loxodonta_G3UE48_G3TIC0

>Callithrix_U3DP14_U3D607

>Myotis_G1QDY4_G1PSJ6

>Pteropus_ENSPVAT00000008600_ENSPVAT00000008862

>Mustela_M3YVG8_M3XR96

>Pan_H2QDE6_H2QDE6

>Nomascus_ENSNLET00000011502_ENSNLET00000019765

>Macaca_H9Z595_H9Z2D1

>Otolemur_ENSOGAT00000024752_ENSOGAT00000005979

>Rattus_P02454_P02466

>Mus_P11087_Q91VL4

>Homo_P02452_P08123

>Ochotona_ENSOPRT00000013202_ENSOPRT00000011598

>Sarcophilus_G3WK23_G3VSR0

**Sequences:**

**>Macrauchenia**

**XXXXXXXXXXXX?XXXXXXXXXXXXXXXXXXXXXXXXXXXXXXXXXXXXXXXXXXXXXXG**

**PPGPPGKNGDDGEAGKPGRXXXXXXXXXXXXXGLPGTAGLPGMKXXXXXXXXXXXXGDAG**

**PAGPKGEPGSPGENGAPGQMGPRXXXXXXGRPGAPGPAGARGNDGATGAAGPPGPTGPAG**

**PPGFPGAVGAKGEAGPQGARGSEGPQGVRXXXXXXXXXXXXXXXXXXXXXXXXXXXGANG**

**APGIAGAPGFPGARGPSGPQGPSGPPGPKGNSGEPGAPGSKXXXXXXGEPGPTGVQGPPG**

**PAGEEGKRXXXGEPGPTGLPGPPGERXXXXXXGFPGSDGIAGPKXXXXXXXXXXXXXXXG**

**SPGEAGRPGEAGLPGAKGLTGSPGSPGPDGKTGPPGPAGQDGRPGPPGPPGARGQAGVMG**

**FPGPKXXXXXXXXXXXXGVPGPPGAVGPAGKDGEAGAQGPPGPAGPAGERXXXXXXXXXX**

**XXXXXXXXXXXXXXXXXXXXXXXXXXXXXXXXXXXXXXXXXXXXGVQGPPGPAGPRGSNG**

**APGNDGAKGDAGAPGAPGSQGAPGLQGMPGERGAAGLPGPKGDRXXXXXXGADGSPGKDG**

**VRGLTGPIGPPGPAGAPGDKGESGPSGPAGPTGARGAPGDRGEPGPPGPAGFAGPPGADG**

**QPGAKXXXXXXXXXGDAGPAGPAGPTGPPGPIGNVGAPGPKXXXGSAGPPGATGFPGAAG**

**RVGPPGPSGNAGPPGPPGPVGKXXXXXXXGETGPAGRPGEVGPPGPPGPSGEKGSPGADG**

**PAGAPGTPGPQGIAGQRGVVGLPGQRXXXGFPGLPGPSGEPGKXXXXXXXXXXGPPGPIG**

**PPGLAGPPGESGREGAPGAEGSPGRDGSPGAKGDRGETGPAGPPGAPGAPGAPGPVGPAG**

**KSGDRGETGPAGPAGPIGPVGARGPAGPQGPRXXXXXXXXXXXXXXXXXXGFSGLQGPPG**

**PPGSPGEQGPSGASGPAGPRGPPGSAGAPGKDGLNGLPGPIGPPGPRXXXXXXXXXXXXX**

**XXXXXXXXXXXXXXXXXXXXXXXXXXXXXXXXXXXXXXXXXX?XX?X?XXXXXXXXXXXX**

**XXXXXXXXXXXXXXXXXXXXXXXXXXXXXXXXXXXXXXXXXXXXXXXXXXXXXXXXXXXX**

**XXXXXGFPGTPGLPGFKXXXGHNGLDGLKGQPGAPGVKXXXXXXXXXXXXXXXXXXXXXX**

**XXXXVGAPGPAGARGSDGSVGPVGPAGPIGSAGPPGFPGAPGPKGELGPVGNPGPAGPAG**

**PRGEVGLPGVSGPVGPPGNPGANGLPGAKGAAGLPGVAGAPGLPGPRGIPGPVGAAGATG**

**ARGLVGEPGPAGTKGESGNKGEPGSAGPQGPPGPSGEEGKRGPNGEAGSTGPAGPPGLRX**

**XXXXXGLPGADGRAGVMGPPGSRGASGPAGVRGPNGDSGRPGEPGLMGPRGFPGSPGNVG**

**PAGKEGPAGLPGIDGRPGPIGPAGARGEPGNIGFPGPKXXXXXXXXXXXXXXXXXXXXXX**

**XXXXXXXXXXXXXXXXXXXXXXXGEQGPAGPPGFQGLPGPAGTAGEAGKPGERGLPGEFG**

**LPGPAGARXXXGPPGESGAAGPSGPIGSRGPSGPPGPDGNKGEPGVVGAPGTAGPSGPSG**

**LPGERGAAGIPGGKXXXXXXXXXGEIGNPGRXXXXGAPGAVGAPGPAGANGDRXXXXXXX**

**XXXXXXXXXXXXXXGEVGPAGPNGFAGPAGAAGQPGAKGERXXXGPKGENGPVGPTGPVG**

**AAGPSGPNGPPGPAGSRGDGGPPGATGFPGAAGRTGPPGPSGITGPPGPPGAAGKXXXXX**

**XXGDQGPVGRAGETGASGPPGFAGEKXXXXXXXXXXXXXXXXXXXXXXXXXXXXXXXXXX**

**XXGLPGVAGSVGEPGPLGIAGPPGARGPPGAVGSPGVNGAPGEAGRXXXXXXXXXXXXXX**

**XXXXXXXXXXXXXXXXXXXXXXXXXXXXXXXXXXHGNRGEPGPVGSVGPAGAVGPRGPSG**

**PQGIRXXXXXXXXXXXXXXXXXXXXXXXXXXXXXXXXXXXXXXXXXXXXXXXXGPAGPTG**

**PAGKDGRIGHPGSVGPAGIRXXXXXXXXXXXXXXXXXXXXXXXXXXXXXXXXXXXXXXX**

**>Toxodon**

**XXXXXXXXXXXX?XXXXXXXXXXXXXXXXXXXXXXXXXXXXXXXXXXXXXXXXXXXXXXG**

**PPGPPGKxxxxxxxxxxxxxxxxxxxxxxxxxxxxxxxxxxxxxxxxxxxxxxxxxxxxx**

**xxxxxGEPGSPGENGAPGQMGPRXXXXXXGRPGAPGPAGARGNDGATGAAGPPGPTGPAG**

**PPGFPGAVGAKxxxxxxxxxGSEGPQGVRGEPGPPGPAGAAGPAGNPGADGQPGAKGANG**

**APGIAGAPGFPGARGPSGPQGPSGPPGPKXXXXXXXXXXXXXXXXXXGEPGPTGVQGPPG**

**PAGEEGKRXXXGEPGPTGLPGPPGERXXXXXXGFPGSDGVAGPKxxxxxxxxxxxxxxxG**

**SPGEAGRPGEAGLPGAKGLTGSPGSPGPDGKxxxxxxxxxxxxxxxxxxxxxxGQAGVMG**

**FPGPKXXXXXXXXXXXXGVPGPPGAVGPAGKXXXXXXXXXXXXXXXXXXXXXXXXXXXXX**

**XXXXXXXXXXXXXXXXXXXXXXXXXXXXXXXXXXXXXXXXXXXXGVQGPPGPAGPRxxxx**

**xxxxxxxxGDAGAPGAPGSQGAPGLQGMPGERGAAGLPGPKxxxXXXXXXxxxxxxxxxx**

**xxGLTGPIGPPGPAGAPGDKGESGPSGPAGPTGARGAPGDRGEPGPPGPAGFAGPPGADG**

**QPGAKGEPGDAGAKGDAGPAGPAGPTGPPGPIGNVGAPGPKxxxGSAGPPGATGFPGAAG**

**RVGPPGPSGNAGPPGPPGPVGKXXXXXXXGETGPAGRPGEVGPPGPPGPSGEKGSPGADG**

**PAGAPGTPGPQGIAGQRGVVGLPGQRXXXGFPGLPGPSGEPGKxxxxxxxxxxGPPGPIG**

**PPGLAGPPGESGRxxxxxxxxxxxxXXXXXXXGDRGETGPAGPPGAPGAPGAPGPVGPAG**

**KSGDRGETGPAGPAGPIGPAGARGPAGPQGPRXXXXXXXXXXXXXXXXXXGFSGLQGPPG**

**PPGSPGEQGPSGASGPAGPRGPPGSAGAPGKDGLNGLPGPIGPPGPRXXXXXXXXXXXXX**

**XXXXXXXXXXXXXXXXXXXXXXXXXXXXXXXXXXXXXXXXXX?XX?X?XXXXXXXXXXXX**

**XXXXXXXXXXXXXXXXXXXXXXXXXXXXXXXXXXXXXXXXxxxxxxxxxxxxxxxxXXXX**

**XXXXXxxxxxxxxxxxxXXXXXXXXXXXXXXXXXXXXXxxxxxxxxxxxxxxxxxxXXXX**

**XXxxxxxxxxxxxxGSDGSVGPVGPAGPIGSAGPPGFPGAPGPKGELGPVGNPGPAGPAG**

**PRGEVGLPGVSGPVGPPGNPGANGLTGAKGAAGLPGVAGAPGLPGPRGIPGPVGAAGATG**

**ARGLVGEPGPAGSKXXXXXXXXXXXXXXXXXXXXXXXXXXRGPNGEAGStGPtGPPGLRX**

**XXXXXxxxxxxxxAGVMGPPGSRXXXXXXXXXXXXXXXXXXXXXXXXXXXGFPGSPGNIG**

**PAGKEGPVGLPGIDGRPGPTGPAGARGEPGNIGFPGPKGPTGDPGKNGDKGHAGLAGARX**

**XXXXXXXXXXXXXXXXXXXXXXXxxxxxxxxxxxxxxxxxxxxxxxxxxxxxxGLPGEFG**

**LPGPAGARGERGPPGESGAVGPSGPIGSRGPSGPPGPDGNKGEPGVLGAPGTAGPSGPSG**

**LPGERGAAGIPGGKGEKxxxxxxXXXXXXXXXXXXGAPGAIGAPGPAGANGDRXXXXXXX**

**XXXXXXXXXXXXXXGEVGPAGPNGFAGPAGAAGQPGAKGERXXXXXXGENGPVGPTGPVG**

**SAGPSGPNGPPGPAGSRGDGGPPGATGFPGAAGRTGPPGPSGITGPPGPPGAAGKXXXXX**

**XXxxxxxxxxXXXXXXXXXXXXXXXXxxxxxxxxxxxxxxxxxxxxxxxxxxxxxxxxxx**

**xxGLPGVAGSLGEPGPLGIAGPPGARGPPGAVGNPGVNGAPGEAGRXXXXXXXXXXXXXX**

**XXXXXXXXXXXXXXXXXXXXXXXXXXXXXXXXXXxxxxGEPGPAGSVGPAGAVGPRGPSG**

**PQGIRXXXXXXXXXXXXXXXXXXXXXXXXXXXXXXXXXXXXXXXXXXXXXXXXGPAGPSG**

**PAGKDGRIGHPGTVGPAGIRXXXXXXXXXXXXXXXXXXXXXXXXXXXXXXXXXXXXXXX**

**>Macrauchenia_PMF**

**XXXXXXXXXXXX?XXXXXXXXXXXXXXXXXXXXXXXXXXXXXXXXXXXXXXXXXXXXXXx**

**xxxxxxxxxxxxxxxxxxxXXXXXXXXXXXXXxxxxxxxxxxxxXXXXXXXXXXXXxxxx**

**xxxxxXXXXXXXXXXXXXXXXXXXXXXXXGRPGAPGPAGARXXXXXXXXXXXXXXXXXXX**

**XXXXXXXXXXXXXXXXXXXXXXXXXXXXXXXXXXXXXXXXXXXXXXXXXXXXXXXXGANG**

**APGIAGAPGFPGARGPSGPQGPSGPPGPKXXXXXXXXXXXXXXXXXXGEPGPTGVQGPPG**

**PAGEEGKRXXXGEPGPTGLPGPPGERXXXXXXXXXXXXXXXXXXXXXXXXXXXXXXXXXG**

**SPGEAGRPGEAGLPGAKXXXXXXXXXXXXXXXXXXXXXXXXXXXXXXXXXXXXGQAGVMG**

**FPGPKXXXXXXXXXXXXXXXXXXXXXXXXXXDGEAGAQGPPGPAGPAGERXXXXXXXXXX**

**XXXXXXXXXXXXXXXXXXXXXXXXXXXXXXXXXXXXXXXXXXXXGVQGPPGPAGPRXXXX**

**XXXXXXXXGDAGAPGAPGSQGAPGLQGMPGERXXXXXXXXXXXXXXXXXXXXXXXXXXXX**

**XXGLTGPIGPPGPAGAPGDKGESGPSGPAGPTGARGAPGDRXXXXXXXXXXXXXXXXXXX**

**XXXXXXXXXXXXXXGDAGPAGPAGPTGPPGPIGNVGAPGPKXXXGSAGPPGATGFPGAAG**

**RVGPPGPSGNAGPPGPPGPVGKXXXXXXXXXXXXXXXXXXXXXXXXXXXXXXXGSPGADG**

**PAGAPGTPGPQGIAGQRGVVGLPGQRXXXXXXXXXXXXXXXXXXXXXXXXXXXxxxxxxx**

**xxxxxxxxxxxxxxxxxxxxxxxxxxxxxxxxxxxxxxxxxxxxxxxxxxxxxxxxxxxx**

**xSGDRGETGPAGPAGPIGPVGARxxxxxxxxxXXXXXXXXXXXXXXXXXXGFSGLQGPPG**

**PPGSPGEQGPSGASGPAGPRGPPGSAGAPGKDGLNGLPGPIGPPGPRXXXXXXXXXXXXX**

**XXXXXXXXXXXXXXXXXXXXXXXXXXXXXXXXXXXXXXXXXX?XX?X?XXXXXXXXXXXX**

**XXXXXXXXXXXXXXXXXXXXXXXXXXXXXXXXXXXXXXXXXXXXXXXXXXXXXXXXXXXX**

**XXXXXxxxxxxxxxxxxXXXxxxxxxxxxxxxxxxxxxXXXXXXXXXXXXXXXXXXXXXX**

**XXXXxxxxxxxxxxxxxxxxxxxxxxxxxxxxxxxxxxxxxxxxxxxxxxxxxxxxxxxx**

**xxxxxxxxxxxxxxxxxxxxxxxxxxxxxGAAGLPGVAGAPGLPGPRGIPGPVGAAGATG**

**ARxxxxxxxxxxxxxxxxxxxxxxxxxxxxxxxxxxxxxxRGPNGEAGSTGPAGPPGLRX**

**XXXXXxxxxxxxxxxxxxxxxxxGASGPAGVRxxxxxxxxxxxxxxxxxxxxxxxxxxxx**

**xxxxEGPAGLPGIDGRxxxxxxxxxxGEPGNIGFPGPKXXXXXXXXXXXXXXXXXXXXXX**

**XXXXXXXXXXXXXXXXXXXXXXXxxxxxxxxxxxxxxxxxxxxxxxxxxxxxxGLPGEFG**

**LPGPAGARXXXGPPGESGAAGPSGPIGSRxxxxxxxxxxxxxxxxxxxxxxxxxxxxxxx**

**xxxxxxxxxxxxxxXXXXXXXXXxxxxxxxxXXXXxxxxxxxxxxxxxxxxxxXXXXXXX**

**XXXXXXXXXXXXXXGEVGPAGPNGFAGPAGAAGQPGAKGERXXXxxxGENGPVGPTGPVG**

**AAGPSGPNGPPGPAGSRGDGGPPGATGFPGAAGRxxxxxxxxxxxxxxxxxxxxxXXXXX**

**XXxxxxxxxxxxxxxxxxxxxxxxxxXXXXXXXXXXXXXXXXXXXXXXXXXXXXXXXXXX**

**XXGLPGVAGSVGEPGPLGIAGPPGARxxxxxxxxxxxxxxxxxxxxXXXXXXXXXXXXXX**

**XXXXXXXXXXXXXXXXXXXXXXXXXXXXXXXXXXHGNRGEPGPVGSVGPAGAVGPRxxxx**

**xxxxxXXXXXXXXXXXXXXXXXXXXXXXXXXXXXXXXXXXXXXXXXXXXXXXXxxxxxxx**

**xxxxxxxIGHPGSVGPAGIRXXXXXXXXXXXXXXXXXXXXXXXXXXXXXXXXXXXXXXX**

**>Toxodon_PMF**

**XXXXXXXXXXXX?XXXXXXXXXXXXXXXXXXXXXXXXXXXXXXXXXXXXXXXXXXXXXXx**

**xxxxxxxxxxxxxxxxxxxxxxxxxxxxxxxxxxxxxxxxxxxxxxxxxxxxxxxxxxxx**

**xxxxxXXXXXXXXXXXXXXXXXXXXXXXXGRPGAPGPAGARXXXXXXXXXXXXXXXXXXX**

**XXXXXXXXXXXxxxxxxxxxXXXXXXXXXXXXXXXXXXXXXXXXXXXXXXXXXXXXGANG**

**APGIAGAPGFPGARGPSGPQGPSGPPGPKXXXXXXXXXXXXXXXXXXGEPGPTGVQGPPG**

**PAGEEGKRXXXGEPGPTGLPGPPGERXXXXXXXXXXXXXXXXXXxxxxxxxxxxxxxxxG**

**SPGEAGRPGEAGLPGAKXXXXXXXXXXXXXXxxxxxxxxxxxxxxxxxxxxxxGQAGVMG**

**FPGPKXXXXXXXXXXXXXXXXXXXXXXXXXXXXXXXXXXXXXXXXXXXXXXXXXXXXXXX**

**XXXXXXXXXXXXXXXXXXXXXXXXXXXXXXXXXXXXXXXXXXXXGVQGPPGPAGPRxxxx**

**xxxxxxxxGDAGAPGAPGSQGAPGLQGMPGERXXXXXXXXXxxxXXXXXXxxxxxxxxxx**

**xxGLTGPIGPPGPAGAPGDKGESGPSGPAGPTGARGAPGDRXXXXXXXXXXXXXXXXXXX**

**XXXXXXXXXXXXXXGDAGPAGPAGPTGPPGPIGNVGAPGPKxxxGSAGPPGATGFPGAAG**

**RVGPPGPSGNAGPPGPPGPVGKXXXXXXXXXXXXXXXXXXXXXXXXXXXXXXXGSPGADG**

**PAGAPGTPGPQGIAGQRGVVGLPGQRXXXXXXXXXXXXXXXXXxxxxxxxxxxxxxxxxx**

**xxxxxxxxxxxxxxxxxxxxxxxxxXXXXXXXxxxxxxxxxxxxxxxxxxxxxxxxxxxx**

**xSGDRGETGPAGPAGPIGPAGARGPAGPQGPRXXXXXXXXXXXXXXXXXXGFSGLQGPPG**

**PPGSPGEQGPSGASGPAGPRGPPGSAGAPGKDGLNGLPGPIGPPGPRXXXXXXXXXXXXX**

**XXXXXXXXXXXXXXXXXXXXXXXXXXXXXXXXXXXXXXXXXX?XX?X?XXXXXXXXXXXX**

**XXXXXXXXXXXXXXXXXXXXXXXXXXXXXXXXXXXXXXXXxxxxxxxxxxxxxxxxXXXX**

**XXXXXxxxxxxxxxxxxXXXXXXXXXXXXXXXXXXXXXxxxxxxxxxxxxxxxxxxXXXX**

**XXxxxxxxxxxxxxxxxxxxxxxxxxxxxxxxxxxxxxxxxxxxGELGPVGNPGPAGPAG**

**PRxxxxxxxxxxxxxxxxxxxxxxxxxxxGAAGLPGVAGAPGLPGPRGIPGPVGAAGATG**

**ARxxxxxxxxxxxxXXXXXXXXXXXXXXXXXXXXXXXXXXRGPNGEAGStGPtGPPGLRX**

**XXXXXxxxxxxxxxxxxxxxxxxXXXXXXXXXXXXXXXXXXXXXXXXXXXxxxxxxxxxx**

**xxxxEGPVGLPGIDGRxxxxxxxxxxGEPGNIGFPGPKxxxxxxxxxxxxxxxxxxxxxX**

**XXXXXXXXXXXXXXXXXXXXXXXxxxxxxxxxxxxxxxxxxxxxxxxxxxxxxGLPGEFG**

**LPGPAGARGERGPPGESGAVGPSGPIGSRxxxxxxxxxxxxxxxxxxxxxxxxxxxxxxx**

**xxxxxxxxxxxxxxxxxxxxxxxXXXXXXXXXXXXxxxxxxxxxxxxxxxxxxxXXXXXX**

**XXXXXXXXXXXXXXGEVGPAGPNGFAGPAGAAGQPGAKGERXXXXXXGENGPVGPTGPVG**

**SAGPSGPNGPPGPAGSRGDGGPPGATGFPGAAGRxxxxxxxxxxxxxxxxxxxxxXXXXX**

**XXxxxxxxxxXXXXXXXXXXXXXXXXxxxxxxxxxxxxxxxxxxxxxxxxxxxxxxxxxx**

**xxGLPGVAGSLGEPGPLGIAGPPGARxxxxxxxxxxxxxxxxxxxxXXXXXXXXXXXXXX**

**XXXXXXXXXXXXXXXXXXXXXXXXXXXXXXXXXXxxxxGEPGPAGSVGPAGAVGPRxxxx**

**xxxxxXXXXXXXXXXXXXXXXXXXXXXXXXXXXXXXXXXXXXXXXXXXXXXXXxxxxxxx**

**xxxxxxxIGHPGTVGPAGIRXXXXXXXXXXXXXXXXXXXXXXXXXXXXXXXXXXXXXXX**

**>Equus**

**QLSYGYDEKSAG-ISVPGPMGPSGPRGLPGPPGAPGPQGFQGPPGEPGEPGASGPMGPRG**

**PPGPPGKNGDDGEAGKPGRPGERGPPGPQGARGLPGTAGLPGMKGHRGFSGLDGAKGDAG**

**PAGPKGEPGSPGENGAPGQMGPRGLPGERGRPGAPGPAGARGNDGATGAAGPPGPTGPAG**

**PPGFPGAVGAKGEAGPQGARGSEGPQGVRGEPGPPGPAGAAGPAGNPGADGQPGAKGANG**

**APGIAGAPGFPGARGPSGPQGPSGPPGPKGNSGEPGAPGNKGDTGAKGEPGPTGIQGPPG**

**PAGEEGKRGARGEPGPTGLPGPPGERGGPGARGFPGADGVAGPKGPAGERGAPGPAGPKG**

**SPGEAGRPGEAGLPGAKGLTGSPGSPGPDGKTGPPGPAGQDGRPGPPGPPGARGQAGVMG**

**FPGPKGAAGEPGKAGERGVPGPPGAVGPAGKDGEAGAQGPPGPAGPAGERGEQGPAGSPG**

**FQGLPGPAGPPGESGKPGEQGVPGDLGAPGPSGARGERGFPGERGVQGPPGPAGPRGSNG**

**APGNDGAKGDAGAPGAPGSQGAPGLQGMPGERGAAGLPGPKGDRGDAGPKGADGSPGKDG**

**VRGLTGPIGPPGPAGAPGDKGETGPSGPAGPTGARGAPGDRGEPGPPGPAGFAGPPGADG**

**QPGAKGEPGDAGAKGDAGPPGPAGPAGPPGPIGSVGAPGPKGARGSAGPPGATGFPGAAG**

**RVGPPGPSGNAGTPGPPGPVGKEGGKGPRGETGPAGRPGEAGPPGPPGPSGEKGSPGADG**

**PAGAPGTPGPQGIAGQRGVVGLPGQRGERGFPGLPGPSGEPGKQGPSGASGERGPPGPVG**

**PPGLAGPPGESGREGAPGAEGSPGRDGSPGPKGDRXXXXXXXXXXXXXXXXXXXXXXXXX**

**XXXXXXXXGPAGPAGPIGPVGARGPAGPQGPRGDKGETGEQGDRGIKGHRGFSGLQGPPG**

**PPGSPGEQGPSGASGPAGPRGPPGSAGAPGKDGLNGLPGPIGPPGPRGRTGDAGPVGPPG**

**PPGPPGPPGPPSGGFDFSFLPQPPQEKSHDGGRYYRARQFDA-KG-G-GPGPMGLMGPRG**

**PPGASGAPGPQGFQGPAGEPGEPGQTGPAGARGPPGPPGKAGEDGHPGKPGRPGERGVVG**

**PQGARGFPGTPGLPGFKGIRGHNGLDGLKGQPGAPGVKGEPGAPGENGTPGQAGARGLPG**

**ERGRVGAPGPAGARGSDGSVGPVGPAGPIGSAGPPGFPGAPGPKGELGPVGNPGPAGPAG**

**PRGEVGLPGLSGPVGPPGNPGANGLTGAKGAAGLPGVAGAPGLPGPRGIPGPAGAAGATG**

**ARGLVGEPGPAGSKGESGNKGEPGAAGPQGPPGPSGEEGKRGPNGEPGSTGPAGPPGLRG**

**SPGSRGLPGADGRAGVMGPAGSRGASGPAGVRGPNGDSGRPGEPGLMGPRGFPGSPGNIG**

**PAGKEGPVGLPGIDGRPGPIGPAGARGEPGNIGFPGPKGPSGEPGKPGDKGHAGLAGARG**

**APGPDGNNGAQGPPGPQGVQGGKGEQGPAGPPGFQGLPGPAGTAGEVGKPGERGLPGEFG**

**LPGPAGARGERGPPGESGAAGPAGPIGSRGPSGPPGPDGNKGEPGVLGAPGTAGPSGPSG**

**LPGERGAAGIPGGKGEKGETGLRGEIGNPGRDGARGAPGAVGAPGPAGANGDRGEAGAAG**

**PAGPAGPRGSPGERGEVGPAGPNGFAGPAGAAGQPGAKGERGTKGPKGENGPVGPTGPVG**

**AAGPSGPNGPPGPAGSRGDGGPPGVTGFPGAAGRTGPPGPSGISGPPGPPGAAGKEGLRG**

**PRGDQGPVGRAGETGASGPPGFAGEKGPSGEPGTAGPPGTPGPQGLLGAPGILGLPGSRG**

**ERGLPGVAGSLGEPGPLGIAGPPGARGPPGAVGAPGVNGAPGEAGRDGNPGSDGPPGRDG**

**QPGHKGERGYPGNAGPVGAVGAPGPHGPVGPTGKHGHRGEPGPVGSVGPVGAVGPRGPSG**

**PQGVRGDKGEPGDKGPRGLPGLKGHNGLQGLPGLAGQHGDQGAPGSVGPAGPRGPAGPTG**

**PVGKDGRSGQPGTVGPAGVRGSQGSQGPAGPPGPPGPPGPPGPSGGGYDFGYDGDFYRA**

**>Ceratotherium**

**XXXXXXXXXXXX?XXXXXXXGPSGPRGLPGPPGAPGPQGFQGPPGEPGEPGASGPMGPRG**

**PPGPPGKNGDDGEAGKPGRPGERGPPGPQGARGLPGTAGLPGMKGHRGFSGLDGAKGDAG**

**PAGPKGEPGSPGENGAPGQMGPRGLPGERGRPGAPGPAGARGNDGATGAAGPPGPTGPAG**

**PPGFPGAVGAKGEAGPQGARGSEGPQGVRGEPGPPGPAGAAGPAGNPGADGQPGAKGANG**

**APGIAGAPGFPGARGPSGPQGPSGPPGPKGNSGEPGAPGSKGDTGAKGEPGPTGIQGPPG**

**PAGEEGKRGARGEPGPTGLPGPPGERXXXXXXXXXXXXXXXXXXGPAGERGAPGPAGPKG**

**SPGEAGRPGEAGLPGAKGLTGSPGSPGPDGKTGPPGPAGQDGRPGPPGPPGARGQAGVMG**

**FPGPKGAAGEPGKAGERGVPGPPGAVGPAGKDGEAGAQGPPGPAGPAGERGEQGPAGSPG**

**FQGLPGPAGPPGESGKPGEQGVPGDLGAPGPSGARGERGFPGERGVQGPPGPAGPRGANG**

**APGNDGAKGDAGAPGAPGSQGAPGLQGMPGERGAAGLPGPKGDRGDAGPKGADGAPGKDG**

**VRGLTGPIGPPGPAGAPGDKGESGPSGPAGPTGARGAPGDRGEPGPPGPAGFAGPPGADG**

**QPGAKGEPGDAGAKGDAGPPGPAGPTGPPGPIGSVGAPGPKGARGSAGPPGATGFPGAAG**

**RVGPPGPSGNAGPPGPPGPVGKEGGKGPRGETGPAGRPGEAGPPGPPGPSGEKGSPGADG**

**PAGAPGTPGPQGIAGQRGVVGLPGQRGERGFPGLPGPSGEPGKQGPSGASGERGPPGPVG**

**PPGLAGPPGESGREGAPGAEGSPGRDGSPGPKGDRGETGPAGPPGAPGAPGAPGPVGPAG**

**KSGDRGETGPAGPAGPVGPVGARGPAGPQGPRGDKGETGEQGDRGIKGHRGFSGLQGPPG**

**PPGSPGEQGPSGASGPAGPRGPPGSAGAPGKDGLNGLPGPIGPPGPRGRTGEAGPVGPPG**

**PPGPPGPPGPPSAGFDFSFLPQPPQEKAHDGGRYYRARXXXX?XX?X?XXXXXGLMGPRG**

**PPGASGAPGPQGFQGPAGEPGEPGQTGPAGARGPPGPPGKAGEDGHPGKPGRPGERGVVG**

**PQGARGFPGTPGLPGFKGIRGHNGLDGLKGQPGAPGVKGEPGAPGENGTPGQXGARGLPG**

**ERGRVGAPGPAGARGSDGSVGPVGPAGPIGSAGPPGFPGAPGPKGELGPVGNPGPAGPAG**

**PRGEVGLPGLSGPVGPPXXXXXXXXXXXXXXXGLPGVAGAPGLPGPRGIPGPAGAAGATG**

**ARGLVGEPGPAGSKGESGNKGEPGSVGAQGPPGPSGEEGKRGPNGEAGSTGPAGPPGLRG**

**SPGSRGLPGADGRAGVMGLAGSRGATGPAGARGPSGDSGRPGEPGLMGPRXXXXXXXXXX**

**XXXXXXXXGLPGIDGRPGPVGPAGARGEPGNIGFPGPKGPXXXXXXXXXXXXXXXXXXXG**

**APGPDGNNGAQGPPGPQGVQGGKGEQGPAGPPGFQGLPGPAGTAGEVGKPGERXXXXXXX**

**XXXXXXXXGERGPPGESGAAGPAGPIGSRGPSGAPGPDGNKGEPGVLGAPGTAGPSGPSG**

**LPGERGAAGIPGGKGEKGETGLRGEIGNSGRDGARGPPGAVGAPGPAGANGDRXXXXXXX**

**XXXXXXXXXXXGERGEVGPAGPNGFAGPAGAAGQPGAKGERGTKGPKGEIGPVGPTGAVG**

**SAGPSGPNGPPGPAGSRGDGGPPGATGFPGAAGRTGPPGPSGITGPPGPPGAAGKEGVRG**

**PRGDQGPVGRAGETGSSGPPGFAGEKGPSGEPGTAGPPGTPGPQGLLGAPGILGLPGSRG**

**ERGLPGVAGSXGEPGPLGIAGPPGARGPPGAVGAPGVNGAPGETGRDGNPGNDGPPGRDG**

**QPGHKGERGYPGNAGPVGAVGAPGSHGPVGPTGKHGNRGEPXXXXXXXXXXXXXXXXXXG**

**PQGVRGDKGEPGDKGPRGLPGLKGHGGLQGLPGLAGHHGDQGAPGSVGPAGPRGPAGPTG**

**PVGKDGRSGQPGTVGPAGVRGSQGSQGPAXXXXXXXXXXXXXXXXXXXXXXXXXXXXXX**

**>Vicugna**

**QMSYGYDEKSTG-ISVPGPMGPSGPRGLPGPPGAPGPQGFQGPPGEPGEPGSSGPMGPRG**

**PPGPPGKNGDDGEAGKPGRPGERGPPGPQGARGLPGTAGLPGMKGHRGFSGLDGAKGDAG**

**PAGPKGEPGSPGENGAPGQMGPRGLPGERGRPGAPGPAGARGNDGATGAAGPPGPTGPAG**

**PPGFPGAVGAKGEAGPQGARGSEGPQGVRGEPGPPGPAGAAGPAGNPGADGQPGAKGANG**

**APGIAGAPGFPGARGPSGPQGPSGPPGPKGNSGEPGAPGNKGDTGAKGEPGPTGVQGPPG**

**PAGEEGKRGARGEPGPAGLPGPPGERGGPGSRGFPGADGVAGPKGPAGERGSPGPAGPKG**

**SPGEAGRPGEAGLPGAKGLTGSPGSPGPDGKTGPPGPAGQDGRPGPPGPPGARGQAGVMG**

**FPGPKGAAGEPGKAGERGVPGPPGAVGPAGKDGEAGAQGPPGPAGPAGERGEQGPAGSPG**

**FQGLPGPAGPPGEAGKPGEQGVPGDLGAPGPSGARGERGFPGERGVQGPPGPAGPRGANG**

**APGNDGAKGDAGAPGAPGSQGAPGLQGMPGERGAAGLPGPKGDRGDAGPKGADGSPGKDG**

**VRGLTGPIGPPGPAGAPGDKGETGPSGPAGPTGARGAPGDRGEPGPPGPAGFAGPPGADG**

**QPGAKGEPGDAGAKGDAGPPGPAGPTGPPGPIGSVGAPGPKGARGSAGPPGATGFPGAAG**

**RVGPPGPSGNAGPPGPPGPVGKEGSKGPRGETGPAGRPGEVGPPGPPGPAGEKGAPGADG**

**PAGAPGTPGPQGIAGQRGVVGLPGQRGERGFPGLPGPSGEPGKQGPSGPNGERGPPGPMG**

**PPGLAGPPGESGREGAPGAEGSPGRDGSPGAQGDRGETGPAGPPGAPGAPGAPGPVGPAG**

**KSGDRGETGPAGPAGPIGPVGARGPAGPQGPRGDKGETGEQGDRGIKGHRGFSGLQGPPG**

**PPGSPGEQGPSGASGPAGPRGPPGSAGAPGKDGLNGLPGPIGPPGPRGRTGDAGPXXXXX**

**XXXPLGPPGPPSGGFDFSFLPQPPQEKxxxxxxxxxxRQFDG-KGxxxGPGPMGLMGPRG**

**PPGAAGAPGPQGFQGPAGEPGEPGQTGPAGARGPPGPPGKAGEDGHPGKPGRPGERGVVG**

**PQGARGFPGTPGLPGFKGIRGHNGLDGLKGQPGAPGVKGEPGAPGENGTPGQTGARGLPG**

**ERGRVGAPGPAGARGSDGSVGPVGPAGPIGSAGPPGFPGAPGPKGELGPVGNPGPAGPAG**

**PRGEVGLPGVSGPVGPPGNPGANGLTGAKGAAGLPGVAGAPGLPGPRGIPGPTGAAGATG**

**ARGLVGEPGPAGSKGESGNKGEPGAAGPQGPPGPSGEEGKRGPTGEVGSPGPAGPPGLRG**

**NPGSRGLPGADGRAGVMGPAGSRGATGPAGVRGPNGDSGRPGEPGLMGPRGFPGSPGNIG**

**PAGKEGPVGLPGIDGRPGPIGPAGARGEPGNIGFPGPKGPAGEPGKHGEKGHAGLAGARG**

**APGPDGNNGAQGPPGPQGVQGGKGEQGPAGPPGFQGLPGPAGTAGEVGKPGERGLPGEFG**

**LPGPAGPRGERGPPGESGAAGPAGPIGSRGPSGPPGPDGNKGEPGVLGAPGTAGPSGPSG**

**LPGERGAAGIPGGKGEKGETGLRGDVGSPGRDGARGAPGAVGAPGPAGANGDRGEAGPAG**

**AAGPAGPRGSPGERGEVGPAGPNGFAGPAGAAGQPGAKGERGTKGPKGENGPVGPTGPVG**

**AAGPSGPNGPPGPAGSRGDGGPPGATGFPGAAGRTGPPGPSGISGPPGPPGPAGKEGLRG**

**PRGDQGPVGRAGETGASGPPGFAGEKGPSGEPGTAGPPGTPGPQGLLGAPGFLGLPGSRG**

**ERGLPGVAGAVGEPGPLGISGPPGARGPPGGVGSPGVNGAPGEAGRDGNPGSDGPPGRDG**

**QPGHKGERGYPGNAGPTGVVGAPGPQGPVGPAGKHGNRGEPGAAGSVGPTGAIGPRGPSG**

**PQGIRGDKGEPGDKGPRGLPGLKGHNGLQGLPGLAGHHGDQGAPGPVGPAGPRGPAGPSG**

**PAGKDGRSGHPGTVGPAGIRGSQGSQGPAGPPGPPGPPGPPGPSGGGYDFGYDGDFYRA**

**>Pteropus**

**QMSYGYDEKSAG-VSVPGPMGPSGPRGLPGPPGAPGPQGFQGPPGEPGEPGASGPMGPRG**

**PPGPPGKNGDDGEAGKPGRPGERGPPGPQGARGLPGTAGLPGMKGHRGFSGLDGAKGDSG**

**PAGPKGEPGSPGENGAPGQMGPRGLPGERGRPGAPGPAGARGNDGATGAAGPPGPTGPAG**

**PPGFPGAVGAKGEAGPQGSRGSEGPQGVRGEPGPPGPAGAAGPAGNPGADGQPGAKGANG**

**APGIAGAPGFPGARGPSGPQGPGGPPGPKGNSGEPGAPGNKGDAGAKGEPGPTGIQGPPG**

**PAGEEGKRGARGEPGPSGLPGPPGERGGPGSRGFPGADGVAGPKGPAGERGSPGPAGPKG**

**SPGEAGRPGEAGLPGAKGLTGSPGSPGPDGKTGPAGPAGQDGRPxPPgPPxARGQAGVMG**

**FPGXXXXXGEPGKAGERGVPGPPGAVGAAGKDGEAGAQGPPGPAGPAGERGEQGPAGSPG**

**FQGLPGPSGPPGEAGKPGEQGVPGDLGAPGPSGARGERGFPGERGVQGPPGPAGPRGANG**

**APGNDGAKGDAGAPGAPGSQGAPGLQGMPGERGAAGLPGPKGDRGDAGPKGADGAPGKDG**

**VRGLTGPIGPPGPAGAPGDKGESGPSGPAGPTGARGAPGDRGEPGPPGPAGFAGPPGADG**

**QPGAKGEPGDAGAKGDAGPAGPAGPAGPPGPIGNVGAPGPKGARGSAGPPGATGFPGAAG**

**RVGPPGPSGNAGPPGPPGPVGKEGGKGPRGETGPAGRPGEAGPPGPPGPAGEKGSPGADG**

**PAGAPGTPGPQGIAGQRGVVGLPGQRGERGFPGLPGPSGEPGKQGPSGTSGERGPPGPMG**

**PPGLAGPPGESGREGSPGAEGSPGRDGSPGPKGDRGETGPAGAPGAPGAPGAPGPVGPAG**

**KSGDRGETGPAGPAGPVGPVGARGPTGPQGPRGDKGETGEQGDRGIKGHRGFSGLQGPPG**

**PPXXXXXXXXXXXXXXXXXXGPPGSAGAAGKDGLNGLPGPIGPPGPRGRTGDAGPVGPPG**

**PPGPPGPPGPPSGGFDFSFLPQPPQEKAHDGGRYYRARQYDG-KGVGLGPGPMGLMGPRG**

**PPGAAGAPGPQGFQGPAGEPGEPGQTGPAGARGPTGPPGKAGEDGHPGKPGRPGERGVVG**

**PQGARGFPGTPGLPGFKGIRGHNGLDGLKGQPGAPGIKXXXXXXXXXXXXXXXGARGLPG**

**ERGRVGAPGPAGARGSDGSVGPVGPAGPIGSAGPPGFPGAPGPKGELGPVGNPGPAGPAG**

**PRGEVGLPGLSGPVGPPGNPGANGLTGAKGAAGLPGVAGAPGLPGPRGIPGPPGAVGATG**

**ARGLVGEPGPAGSKGESGNKGEPGSAGAQGPPGPSGEEGKRGSNGEAGSAGPPGPPGLRG**

**SPGSRGLPGADGRAGVMGPAGSRGATGPAGVRGPSGDSGRXXXXGLMGPRGFPGSPGNVG**

**PAGKEGPMGLPGIDGRPGPIGPAGARGEPGNIGFPGPKGPTGDPGKSGEKGHAGLAGPRG**

**APGPDGNNGAQGPPGLQGVQGGKGEQGPAGPPGFQGLPGPAGTTGEVGKPGERGLPGEFG**

**LPGPAGPRGERGPPGESGAVGPSGPIGSRGPSGPPGPDGNKGEPGGVGAPGTAGASGSGG**

**LPGERGAAGIPGGKGEKGETGLRGEVGSTGRDGARGAPGAIGAPGPAGATGDRGEAGPAG**

**PAGPAGPRGSPGERGEVGPAGPNGFAGPAGAAGQPGAKGERGTKGPKGENGPVGPTGPAG**

**SSGPAGPNGPPGPAGSRGDGGPPXXXXXXGAAGRTGPSGPSGITGPPGPPGAAGKEGVRG**

**PRGDQGPVGRTGETGAGGPPGFTGEKGPSGEPGTAGPPGTPGPQGLLGAPGILGLPGSRG**

**ERGLPGVAGSVGEPGPLGISGPPGARGPPGAVGNPGVNGAPGEAGRDGNPGNDGPPGRDG**

**QPGHKGERGYPGNPGPVGALGAPGPHGPVGPTGKHGNRGEPGPAGSVGPTGAVGPRGPSG**

**PQGIRGDKGEPGDKGPRGLPGLKGHNGLQGLPGLAGHHGDQGSPGSVGPAGPRGPAGPSG**

**PAGKDGRTGHPGTVGPAGIRGSQGSQGPAGPPGPPGPPGPPGVSGGGYDFGFDGDFYRA**

**>Erinaceus**

**QMSYGYDEKSTGGMSVPGPMGPSGPRGLPGPPGSPGPQGFQGPPGEPGEPGASXXXXXXX**

**XXXXXXXXXXXGEAGKPGRPGERGPPGPQGARGLPGTAGLPGMKGHRGFSGLDGAKGDSG**

**PAGPKGEPGSPGENGAPGQMGPRGLPGERGRPGATGPAGARGNDGATGAAGPPGPTGPAG**

**PPGFPGAVGAKGEAGPQGARGSEGPQGVRGEPGPPGPAGAAGPAGNPGADGQPGAKGANG**

**APGIAGAPGFPGARGPSGPQGPSGPPGPKGNSGEPGAPGNKGDTGAKGEPGPAGVQGPPG**

**PAGEEGKRGARGEPGPTGLPGPPGERGGPGSRGFPGSDGAAGPKGPAGERGSPGPAGPKG**

**SPGEAGRPGEAGLPGAKGLTGSPGSPGPDGKTGPPGPAGQDGRPGPPGPPGARGQAGVMG**

**FPGPKGAAGEPGKAGERGVPGPPGAVXXXXXXXXXXXXXXXXXXGPAGERGEQGPAGSPG**

**FQGLPGPAGPPGEAGKPGEQGAPGDLGAPGPSGARGERGFPGERGVQGPPGPAGPRGSNG**

**APGNDGAKGDAGAPGAPGSQGAPGLQGMPGERGAAGLPGPKGDRGDAGPKGADGSPGKDG**

**VRGLTGPIGPPGPAGAPGDKGESGPSGPAGPTGARGAPGDRGEPGPPGPAGFAGPPGADG**

**QPGAKGEPGDAGAKGDSGPPGPAGPTGPPGPIGNVGAPGPKGARGAAGPPGATGFPGAAG**

**RVGPPGPSGNAGPPGPPGPVGKEGGKGPRGETGPAGRPGEAGPPGPPGPAGEKGSPGADG**

**PAGSPGTPGPQGIAGQRGVVGLPGQRGERGFPGLPGPSGEPGKQGPSGASGERGPPGPMG**

**PPGLAGPPGESGREGSPGAEGSPGRDGSPGPKGDRGETGPAGPPGAPGAPGAPGPVGPAG**

**KSGDRGETGPAGPAGPIGPAGARGPAGPQGPRGDKGETGEQGDRGMKGHRGFSGLQGPPG**

**PPGSPGEQGPSGASGPAGPRGPPGSAGAAGKDGLNGLPGPIGPPGPRGRTGDAGPXGPPG**

**PPGPPGPPGPPSGGFDLNFLPQPPQEKAHDGGRYYRARQYDG-KGVGLGPGPMGLMGPRG**

**PPGASGAPGPPGFQGPAGEPGEPGQTGPAGARGPTGPPGKAGEDGHPGKPGRPGERGVVG**

**PQGARGFPGTPGLPGFKGIRGHNGLDGLKGQPGAPGVKGEPGAPGENGTPGQTGARGLPG**

**ERGRVGAPGPAGARGSDGSVGPVGPAGPIGSAGPPGFPGAPGPKGELGPVGNPGPSGPAG**

**ARGEVGLPGVSGPVGPPGNPGANGLTGAKGAAGLPGVAGAPGLPGPRGIPGPVGAAGASG**

**ARGLVGEPGPAGSKGETGNKGEPGSAGAQGLPGPSGEEGKRGQNGEAGSAGPAGPPGLRG**

**SPGSRGLPGADGRPGVMGPPGSRGASGPAGVRGPSGDSGRPGEPGLMGPRGFPGSPGNVG**

**PAGKEGPSGLPGIDGRPGPIGPAGARGEPGNIGFPGPKGPSGDPGKSGDKGHAGLAGARG**

**APGPDGNNGAQGPPGAQGVQGGKGEQGPAGPPGFQGLPGPAGTTGEVGKPGERGLPGEFG**

**LPGPAGPRGERGPPGQSGAAGPSGPIGSRGPSGSPGPDGNKGEPGVLGAPGTAGPSGPGG**

**LPGERGAAGVPGGKGEKGETGLRGEIGNPGRDGARXXXXXXXXXXXXXXXGDRGEAGPAG**

**PAGPAGPRGSPGERGEVGPAGPNGFAGPAGAAGQPGAKGERGTKGPKGENGIVGPTGPVG**

**AAGPSGPNGPPGPAGGRGDGGPPGATGFPGAAGRTGPPGPSGITGPPGPPGAAGKEGLRG**

**PRGDQGPVGRTGETGGSGPPGFTGEKGPAGEPGTAGPPGTAGPQGLLGAPGILGLPGSRG**

**ERGLPGVFGSVGEPGPLGIAGPPGARGPPGAVGNPGVNGAPGEAGRDGNPGSDGPPGRDG**

**QPGHKGERGYPGNAGSVGAAGAPGPHGSVGPAGKHGNRGEPGPAGAVGPVGAFGPRGPSG**

**PQGIRGDKGEPGDKGPRGLPGLKGHNGLQGLPGLAGQHGDQGAPGSVGPAGPRGPAGPSG**

**PAGKDGHNGQPGTVGPAGIRGSQGNQGPAGPAGPPGPPGPPGPSGGGYDFGYEGDFYRA**

**>Sorex**

**XXXXXXXXXXXX?XXXXXXXGPSGPRGLPGPPGAPGPQGFQGPPGEPGEPGASXXXXXXX**

**XXXXXXXXXXXGEAGKPGRPGERGPPGPQGARGLPGTAGLPGMKGHRGFSGLDGAKGDSG**

**PAGPKGEPGSPGENGAPGQMGPRGLPGERGRPGAPGPAGARGNDGATGAAGPPGPTGPAG**

**PPGFPGAVGAKGEAGPQGARGSEGPQGVRGEPGPPGPAGAAGPAGNPGADGQPGAKGANG**

**APGIAGAPGFPGARGPSGPQGPSGPPGPKGNSGEPGAPGNKGDTGAKGEPGPAGVQGPPG**

**PAGEEGKRGARGEPGPTGLPGPPGERXXXXXXXXXXXXXXXXXXGPAGERGSPGPAGPKG**

**SPGEAGRPGEAGLPGAKGLTGSPGSPGPDGKTGPPGPAGQDGRPGPPGPPGARGQAGVMG**

**FPGPKGAAGEPGKAGERGVPGPPGAVXXXXXXXXXXXXXXXXXXGPAGERGEQGPAGSPG**

**FQGLPGPAGPPGEAGKPGEQGAPGDLGAPGPSGARGERGFPGERGVQGPPGPAGPRGSNG**

**APGNDGAKGDAGAPGAPGSQGAPGLQGMPGERGAAGLPGPKGDRGDAGPKGADGSPGKDG**

**VRGLTGPIGPPGPAGAPGDKGESGPSGPAGPTGARGAPGDRGEPGPPGPAGFAGPPGADG**

**QPGAKGEPGDAGAKGDAGPPGPAGPTGAPGPIGNVGAPGPKGARGSAGPPGATGFPGAAG**

**RVGPPGPSGNAGPPGPPGPVGKEGGKGPRGETGPAGRPGEVGPPGPPGPAGEKGSPGADG**

**PAGSPGTPGPQGIAGQRGVVGLPGQRGERGFPGLPGPSGEPGKQGPSGSSGERGPPGPMG**

**PPGLAGPPGESGREGSPGAEGSPGRDGSPGPKGDRGETGPAGPPGAPGAPGAPGPVGPAG**

**KSGDRGETGPAGPAGPIGPAGARGPAGPQGPRGDKGETGEQGDRGMKGHRGFSGLQGPPG**

**PPGSPGEQGPSGASGPAGPRGPPGSAGAAGKDGLNGLPGPIGPPGPRGRTGDAGPVGPPG**

**PPGPPGPPGPPSGGFDFSFMPQPPQEKAHDGGRYYRARQYDG-KGVGLGPGPMGLMGPRG**

**PPGASGAPGPQGFQGPAGEPGEPGQTGPAGARGPPGPPGKAGEDGHPGKAGRPGERGVVG**

**PQGARGFPGTPGLPGFKGIRGHNGLDGLKGQPGAPGVKGEPGAPGENGTPGQAGARGLPG**

**ERGRVGAPGPAGARGSDGSVGPVGPAGAIGSAGPPGFPGAPGPKGELGAVGNPGPAGPAG**

**ARGEVGLPGVSGPVGPAGNPGANGLTGAKGAAGLPGVAGAPGLPGPRGIPGPVGAAGASG**

**PRGLIGEPGPAGSKGEGGNKGEPGSAGPQGPPGPSGEEGKRGQNGEPGSAGPTGPPGLRG**

**TPGSRGLPGADGRPGVMGPPGSRGASGPAGARGPNGDSGRPGEPGLVGPRGFPGSPGSVG**

**PAGKEGPVGLPGIEGRPGAIGPAGARGEPGNIGFPGPKGPNGEPGKSGDKGHPGLAGARG**

**APGPDGNNGAQGPPGPQGVQGGKGEQGPAGPPGFQGLPGPAGTTGEVGKPGERGLPGEFG**

**LPGPAGPRGERGPPGESGAAGPAGPIGSRGPSGPPGPDGNKGEPGVVGAPGNAGPSGPGG**

**LPGERGAAGIPGGKGEKGETGLRGEIGNPGRDGARGAPGAVGAPGPSGAAGDRGEAGAAG**

**PAGPAGPRGSPGERGEVGPAGPNGFAGPAGAAGQPGAKGERGTKGPKGENGVVGPTGPVG**

**AAGPSGPNGPPGPAGSRGDGGPPGATGFPGAAGRTGPPGPSGITGPPGPPGAAGKEGLRG**

**PRGDQGPVGRTGETGASGLPGFAGEKGPNGEPGTAGPPGTPGPQGLLGAPGILGLPGSRG**

**ERGLPGIAGSVGEPGPLGISGPPGARGPPGAVGNPGVNGAPGEAGRDGNPGSDGPPGRDG**

**QPGHKGERGYPGNAGPVGAVGAPGPHGPVGPTGKHGNRGEPGPAGAVGPAGAFGPRGPSG**

**PQGIRGDKGEPGDKGARGLPGLKGHNGLQGLPGLAGHHGDQGAPGSVGPAGPRGPAGPSG**

**PAGKDGRSGHPGTVGPAGIRGSQGNQGPAGPPGPPGPPGPPGPSGGGYDFGYDGDFYRA**

**>Myotis**

**QMSYGYDEKSAG-VSVPGPMGPSGPRGLPGPPGSPGPQGFQGPPGEPGEPGASGPMGPRG**

**PPGPPGKNGDDGEAGKPGRPGERGPPGPQGARGLPGTAGLPGMKGHRGFSGLDGAKGDAG**

**PAGPKGEPGSPGENGVPGQMGPRGLPGERGRPGAPGPAGARGNDGATGAAGPPGPTGPAG**

**PPGFPGAVGAKGEAGPQGSRGSEGPQGVRGEPGPPGPAGAAGPAGNPGADGQPGAKGANG**

**APGIAGAPGFPGARGPSGPQGPSGAPGPKGNSGEPGAPGNKGDTGAKGEPGPTGIQGPPG**

**PAGEEGKRGARGEPGPAGLPGPPGERGGPGSRGFPGADGVAGPKGPAGERGSPGPAGPKG**

**SPGEAGRPGEAGLPGAKGLTGSPGSPGPDGKTGPTGPAGQDGRPGPPGPPGARGQAGVMG**

**FPGPKGAAGEPGKAGERGVPGPPGAVGPAGKDGEAGAQGAPGPAGPAGERGEQGPAGSPG**

**FQGLPGPAGPPGEAGKPGEQGAPGDLGAPGPSGARGERGFPGERGVQGPPGPAGPRGSNG**

**APGNDGAKGDAGAPGAPGSQGAPGLQGMPGERGAAGLPGPKGDRGDAGPKGADGAPGKDG**

**VRGLTGPIGPPGPAGAPGDKGETGPSGPAGPTGARGAPGDRGEPGPPGPAGFAGPPGADG**

**QPGAKGEPGDAGAKGDAGPAGPAGPAGPPGPIGNVGAPGPKGARGSAGPPGATGFPGAAG**

**RVGPPGPSGNAGPPGPPGPAGKEGGKGPRGETGPAGRPGEVGPPGPPGPAGEKGSPGSDG**

**PAGSPGTPGPQGIAGQRGVVGLPGQRGERGFPGLPGPSGEPGKQGPSGSSGERGPPGPMG**

**PPGLAGPPGESGREGSPGAEGSPGRDGSPGPKGDRGETGPAGPPGAPGAPGAPGPVGPAG**

**KSGDRGETGPAGPAGPIGPAGARGPAGPQGPRGDKGETGEQGDRGIKGHRGFSGLQGPPG**

**PPGSPGDQGPSGASGPAGPRGPPGSPGAAGKDGLNGLAGPIGPPGPRGRTGDAGPVGPPG**

**PPGPPGPPGPPSGGFDFSFMPQPPQEKAHDGGRYYRARQFDG-KGVGGGPGPMGLMGPRG**

**PPGAAGAPGPQGFQGPAGEPGEPGQTGPAGSRGPAGPPGKAGEDGHPGKPGRPGERGVVG**

**PQGARGFPGTPGLPGFKGIRGHNGLDGLKGQPGAPGIKGEPGAPGENGTPGQTGARGLPG**

**ERGRVGAPGPAGARGSDGSVGPVGPAGPIGSAGPPGFPGAPGPKGELGPVGNPGPSGPAG**

**PRGEVGLPGLSGPVGPPGNPGANGLAGAKGAAGLPGVAGAPGLPGPRGIPGPPGAAGAAG**

**PRGLIGEPGPAGSKGETGNKGEPGSAGAQGPPGPSGEEGKRGTAGEAGPAGPPGPAGLRG**

**NPGSRGLPGADGRAGVMGPAGPRGATGPAGARGPNGDAGRPGEPGLMGPRGFPGSPGNVG**

**PAGKEGPVGLPGIDGRPGPIGPAGARGEPGNIGFPGPKGPTGDAGKPGERGHAGLAGARG**

**APGPDGNNGAQGPPGPQGVQGGKGEQGPAGPPGFQGLPGPAGTAGEAGKPGERGLPGEFG**

**LPGPAGPRGERGPPGESGAVGPSGPIGSRGPSGPPGPDGNKGEPGSVGAPGSAGAPGPGG**

**LPGERGAAGIPGGKGDKGEPGLRGEMGTTGRDGARGAPGAMGAPGPSGASGDRGEAGAAG**

**PAGPAGPRGSPGERGEVGPAGPNGFAGPAGAAGQPGAKGERGTKGPKGENGVVGPTGPVG**

**AAGPSGPNGPPGPAGTRGDGGPPGMTGFPGAAGRTGPPGPSGITGPPGPPGASGKEGLRG**

**PRGDQGPVGRTGETGATGPPGFVGEKGPSGEPGAAGPPGTPGPQGLLGAPGILGLPGSRG**

**ERGLPGVSGSVGEPGPLGIAGPPGARGPPGAVGSPGVNGAPGEAGRDGNPGSDGPPGRDG**

**QPGHKGDRGYPGNAGPVGTVGAPGPHGPVGPTGKHGNRGEPGPAGSVGPTGAVGPRGPSG**

**AQGIRGDKGEPGEKGPRGLPGLKGHNGLQGLPGLAGHHGDQGAPGTVGPAGPRGPAGPSG**

**PPGKDGRNGHPGVVGPAGIRGTQGSQGPAGPPGPPGPPGPPGISGGGYDFGFDGDFYRA**

**>Tursiops**

**QMSYGYDEKSTG-ISVPGPMGPSGPRGLPGPPGTPGPQGFQGPPGEPGEPGASGPMGPRG**

**PPGPPGKNGDDGEAGKPGRPGERGPPGPQGARGLPGTAGLPGMKGHRGFSGLDGAKGDAG**

**PAGPKGEPGSPGENGAPGQMGPRGLPGERGRPGAPGPAGARGNDGATGAAGPPGSTGPAG**

**PPGFPGAVGAKGEAGPQGSRGSEGPQGVRGEPGPPGPAGATGPAGNPGADGQPGAKGANG**

**APGIAGAPGFPGARGPSGPQGPSGPPGPKGNSXXXXXXXXXXXXXXXXXXGPTGIQGPPG**

**PAGEEGKRGARGEPGPAGLPGPPGERGGPGSRGFPGADGIAGPKGPAGERGAPGLAGPKX**

**SPGEAGRPGEAGLPGAKGLTGSPGSPGPDGKTGPPGPAGQDGRPGPPGPPGSRGQAGVMG**

**FPGPKGAAGEPGKAGERGVPGPPGAAGPAGKDGEAGAQGPPGPAGPAGERGEQGPAGSPG**

**FQGLPGPSGPPGEAGKPGEQGAPGDLGAPGPSGARGERGFPGERGVQGPSGPAGPRGSNG**

**PPGNDGAKGDAGAPGAPGNQGAPGLQGMPGERGAAGLPGPKGDRGDAGPKGADGAPGKDG**

**VRGLTGPIGPPGPAGAPGDKGETGPSGPAGPTGARGAPGDRGEPGPPGPAGFAGPPGADG**

**QPGAKGEPGDAGAKGDAGAPGSAGPTGPPGPIGNVGAPGPKGARGSAGPPGATGFPGAAG**

**RVGPPGPSGNAGPPGPSGPAGKEGSKGPRGETGPAGRAGEVGPPGPPGPAGEKGAPGADG**

**PAGSPGSPGPQGIAGQRGVVGLPGQRGERGFPGLPGPSGEPGKQGPSGASGERGPPGPMG**

**PPGLAGPPGESGREGAPGAEGSPGRDGSPGPKGDRGETGPAGPPGAPGSPGAPGPVGPAG**

**KSGDRGETGPAGPAGPIGPAGARGPTGPQGPRGDKGETGEQGDRGIKGHRGFSGLQGPPG**

**PPGSPGEQGPSGASGPAGPRGPPGSAGTPGKDGLNGLPGPIGPPGPRGRTGDAGPAGPPG**

**PPGPPGPPGPPSGGYDFSFLPQPPQEKAQDGGRYYRARQYDG-KGVGLGPGPMGLMGPRG**

**PPGASGVPGPQGFQGPPGEPGEPGQTGPAGARGPPGPPGKAGEDGHPGKPGRPGERGVVG**

**PQGARGFPGTPGLPGFKGIRGHNGLDGLKGQPGTPGVKGEPGAPGENGIPGQVGARGLPG**

**ERGRVGAPGPAGARGSDGSVGPVGPAGPVGSAGPPGFPGAPGPKGELGPVGNPGPAGPAG**

**SRGEVGLPGVSGPVGPPGNPGANGLHGAKGAAGLPGVAGAPGLPGPRGIPGPVGAAGATG**

**ARGLVGEPGPAGSKGESGNKGEPGAAGPTGPPGPSGEEGKRGSTGEIGSAGPPGPPGLRG**

**NPGSRGLPGADGRAGVMGPHGSRGGTGPAGVRGPSGDSGRPGEPGLMGPRGFPGSPGNVG**

**PAGKEGPMGLPGIDGRPGPIGPAGARGEPGNIGFPGPKGPTGDPGKNGEKGHAGLAGPRG**

**APGPDGNNGAQGPPGPQGVSGGKGEQGPAGPPGFQGLPGPAGTAGEAGKAGERGLPGEFG**

**LPGPAGPRGERGPPGESGAAGPTGPVGSRGPSGPAGPDGNKGEPGVVGAPGSAGPSGPNG**

**LPGERGAAGIPGGKGEKGETGLRGDAGSHGRDGARGAPGAVGAPGPAGANGDRGEAGPAG**

**PAGPAGPRGSPGERGEVGPAGPNGFAGPAGAAGQPGAKGERGTKGPKGENGPTGPTGPVG**

**AAGPAGPNGPPGPAGSRGDGGPPGATGFPGAAGRTGPPGPSGITGPPGPPGPAGKEGLRG**

**PRGDQGPVGRTGETGASGPPGFVGEKGPSGEPGTAGSPGTPGPQGLLGAPGFLGLPGSRG**

**ERGLPGVAGSVGEPGPLGIAGPTGARGPPGAVGNPGVNGAPGEAGRDGNPGNDGPPGRDG**

**QAGHKGDRGYPGNAGPTGTVGAPGPQGPVGPTGKHGNRGEPGPSGPIGLAGAVGPRGPSG**

**PQGIRGDKGEPGDKGPRGLPGLKGHNGLQGLPGLAGHHGDQGAPGTVGPAGPRGPSGPSG**

**PSGKDGRTGHPGAVGPAGIRGSQGSQGPSGPPGPPGPPGPPGPSGGGYDFGFDGDFYRA**

**>Balaeonoptera**

**QMSYGYDEKSTG-ISVPGPMGPSGPRGLPGPPGAPGPQGFQGPPGEPGEPGASGPMGPRG**

**PPGPPGKNGDDGEAGKPGRPGERGPPGPQGARGLPGTAGLPGMKGHRGFSGLDGAKGDAG**

**PAGPKGEPGSPGENGAPGQMGPRGLPGERGRPGAPGPAGARGNDGATGAAGPPGPTGPAG**

**PPGFPGAVGAKGEAGPQGSRGSEGPQGVRGEPGPPGPAGATGPAGNPGADGQPGAKGANG**

**APGIAGAPGFPGARGPSGPQGPSGPPGPKGNSGEPGAPGNKGDTGAKGEPGPTGIQGPPG**

**PAGEEGKRGTRGEPGPAGLPGPPGERGGPGSRGFPGADGVSGPKGPAGERGAPGPAGPKG**

**SPGEAGRPGEAGLPGAKGLTGSPGSPGPDGKTGPPGPAGQDGRPGPPGPPGSRGQAGVMG**

**FPGPKGAAGEPGKAGERGVPGPPGAVGPAGKDGEAGAQGPPGPAGPAGERGEQGPAGSPG**

**FQGLPGPAGPPGEAGKPGEQGVPGDLGAPGPSGARGERGFPGERGVQGPSGPAGPRGSNG**

**APGNDGAKGDAGAPGAPGNQGAPGLQGMPGERGAAGLPGLKGDRGDVGPKGADGAPGKDG**

**VRGLTGPIGPPGPAGAPGDKGETGPSGPAGPTGARGAPGDRGEPGPPGPAGFAGPPGADG**

**QPGAKGEPGDAGAKGDAGLPGAAGPTGPPGPIGNVGAPGPKGARGSAGPPGATGFPGAAG**

**RVGPPGPSGNAGPPGPSGPAGKEGSKGARGETGPAGRAGEVGPPGPPGPAGEKGAPGADG**

**PAGAPGSPGPQGIAGQRGVVGLPGQRGERGFPGLPGPSGEPGKQGPSGASGERGPPGPMG**

**PPGLAGPPGESGREGAPGAEGSPGRDGSPGPKGDRGETGPAGPPGAPGAPGAPGPVGPAG**

**KSGDRGETGPAGPAGPIGPVGARGPAGPQGPRGDKGETGEQGDRGIKGHRGFSGLQGPPG**

**PPGSPGEQGPSGASGPAGPRGPPGSAGTPGKDGLNGLPGPIGPPGPRGRTGDAGPAGPPG**

**PPGPPGPPGPPSGGYDFSFMPQPPQEKAQDGGRYYRARQFDA-KGVG?GPGPMGLMGPRG**

**PPGASGAPGPQGFQGLPGEPGEPGQTGPAGSRGPPGPPGKAGEDGHPGKPGRPGERGVVG**

**PQGARGFPGTPGLPGFKGIRGHNGLDGLKGQPGAPGVKGEPGAPGENGTPGQTGARGLPG**

**ERGRVGAPGPAGARGSDGSVGPVGPAGPIGSAGPPGFPGAPGPKGELGPVGNPGPPGPAG**

**SRGEVGLAGVSGPVGPPGNPGANGLPGAKGAAGLPGVAGAPGLPGPRGIPGPVGAAGATG**

**ARGLVGEPGPAGSKGESGNKGEPGAAGPTGPPGPSGEEGKRGTTGEIGSAGPPGPPGLRG**

**NPGSRGLPGADGRAGVMGPHGSRGGTGPAGMRGPSGDSGRPGEPGLMGPRGFPGSPGNVG**

**PAGKEGPVGLPGIDGRPGAIGPAGARGEPGNIGFPGPKGPSGDPGKAGEKGHAGLAGARG**

**APGPEGNNGAQGPPGLQGVSGGKGEQGPAGPPGFQGLPGPAGTAGEAGKPGERGLPGEFG**

**LPGPAGARGERGPPGESGAAGPTGPIGNRGPSGPAGPDGNKGEPGVVGAPGTAGPSGPSG**

**LPGERGAAGIPGGKGEKGETGLRGDIGSPGRDGARGAPGAVGAPGPAGANGDRGEAGPAG**

**PAGPAGPRGSPGERGEVGPAGPNGFAGPAGAAGQPGAKGERGTKGPKGENGPAGPTGPVG**

**AAGPSGPNGPPGPAGSRGDGGPPGVTGFPGAAGRTGPPGPSGITGPPGPTGPAGKEGLRG**

**PRGDQGPVGRTGETGASGPPGFVGEKGPSGEPGTAGSPGTPGPQGLLGAPGFLGLPGSRG**

**ERGLPGVAGSVGEPGPLGISGPTGARGPPGAVGNPGVNGAPGEAGRDGNPGNDGPPGRDG**

**QPGHKGDRGYPGNAGPTGTAGAPGPQGPQGPVGKHGNRGEPGPAGAVGPAGAVGPRGPSG**

**PQGIRGDKGEPGDKGPRGLPGLKGHNGLQGLPGLKGHHGDQGAPGTVGPAGPRGPAGPSG**

**PSGKDGRTGHPGAVGPAGIRGSQGSQGPAGPPGPPGPPGPPGPSGGGYEFGFDGDFYRA**

**>Bos**

**QLSYGYDEKSTG-ISVPGPMGPSGPRGLPGPPGAPGPQGFQGPPGEPGEPGASGPMGPRG**

**PPGPPGKNGDDGEAGKPGRPGERGPPGPQGARGLPGTAGLPGMKGHRGFSGLDGAKGDAG**

**PAGPKGEPGSPGENGAPGQMGPRGLPGERGRPGAPGPAGARGNDGATGAAGPPGPTGPAG**

**PPGFPGAVGAKGEGGPQGPRGSEGPQGVRGEPGPPGPAGAAGPAGNPGADGQPGAKGANG**

**APGIAGAPGFPGARGPSGPQGPSGPPGPKGNSGEPGAPGSKGDTGAKGEPGPTGIQGPPG**

**PAGEEGKRGARGEPGPAGLPGPPGERGGPGSRGFPGADGVAGPKGPAGERGAPGPAGPKG**

**SPGEAGRPGEAGLPGAKGLTGSPGSPGPDGKTGPPGPAGQDGRPGPPGPPGARGQAGVMG**

**FPGPKGAAGEPGKAGERGVPGPPGAVGPAGKDGEAGAQGPPGPAGPAGERGEQGPAGSPG**

**FQGLPGPAGPPGEAGKPGEQGVPGDLGAPGPSGARGERGFPGERGVQGPPGPAGPRGANG**

**APGNDGAKGDAGAPGAPGSQGAPGLQGMPGERGAAGLPGPKGDRGDAGPKGADGAPGKDG**

**VRGLTGPIGPPGPAGAPGDKGEAGPSGPAGPTGARGAPGDRGEPGPPGPAGFAGPPGADG**

**QPGAKGEPGDAGAKGDAGPPGPAGPAGPPGPIGNVGAPGPKGARGSAGPPGATGFPGAAG**

**RVGPPGPSGNAGPPGPPGPAGKEGSKGPRGETGPAGRPGEVGPPGPPGPAGEKGAPGADG**

**PAGAPGTPGPQGIAGQRGVVGLPGQRGERGFPGLPGPSGEPGKQGPSGASGERGPPGPMG**

**PPGLAGPPGESGREGAPGAEGSPGRDGSPGAKGDRGETGPAGPPGAPGAPGAPGPVGPAG**

**KSGDRGETGPAGPAGPIGPVGARGPAGPQGPRGDKGETGEQGDRGIKGHRGFSGLQGPPG**

**PPGSPGEQGPSGASGPAGPRGPPGSAGSPGKDGLNGLPGPIGPPGPRGRTGDAGPAGPPG**

**PPGPPGPPGPPSGGYDLSFLPQPPQEKAHDGGRYYRARQFDA-KG-G-GPGPMGLMGPRG**

**PPGASGAPGPQGFQGPPGEPGEPGQTGPAGARGPPGPPGKAGEDGHPGKPGRPGERGVVG**

**PQGARGFPGTPGLPGFKGIRGHNGLDGLKGQPGAPGVKGEPGAPGENGTPGQTGARGLPG**

**ERGRVGAPGPAGARGSDGSVGPVGPAGPIGSAGPPGFPGAPGPKGELGPVGNPGPAGPAG**

**PRGEVGLPGLSGPVGPPGNPGANGLPGAKGAAGLPGVAGAPGLPGPRGIPGPVGAAGATG**

**ARGLVGEPGPAGSKGESGNKGEPGAVGQPGPPGPSGEEGKRGSTGEIGPAGPPGPPGLRG**

**NPGSRGLPGADGRAGVMGPAGSRGATGPAGVRGPNGDSGRPGEPGLMGPRGFPGSPGNIG**

**PAGKEGPVGLPGIDGRPGPIGPAGARGEPGNIGFPGPKGPSGDPGKAGEKGHAGLAGARG**

**APGPDGNNGAQGPPGLQGVQGGKGEQGPAGPPGFQGLPGPAGTAGEAGKPGERGLPGEFG**

**LPGPAGARGERGPPGESGAAGPTGPIGSRGPSGPPGPDGNKGEPGVVGAPGTAGPSGPSG**

**LPGERGAAGIPGGKGEKGETGLRGDIGSPGRDGARGAPGAIGAPGPAGANGDRGEAGPAG**

**PAGPAGPRGSPGERGEVGPAGPNGFAGPAGAAGQPGAKGERGTKGPKGENGPVGPTGPVG**

**AAGPSGPNGPPGPAGSRGDGGPPGATGFPGAAGRTGPPGPSGISGPPGPPGPAGKEGLRG**

**PRGDQGPVGRSGETGASGPPGFVGEKGPSGEPGTAGPPGTPGPQGLLGAPGFLGLPGSRG**

**ERGLPGVAGSVGEPGPLGIAGPPGARGPPGNVGNPGVNGAPGEAGRDGNPGNDGPPGRDG**

**QPGHKGERGYPGNAGPVGAAGAPGPQGPVGPVGKHGNRGEPGPAGAVGPAGAVGPRGPSG**

**PQGIRGDKGEPGDKGPRGLPGLKGHNGLQGLPGLAGHHGDQGAPGAVGPAGPRGPAGPSG**

**PAGKDGRIGQPGAVGPAGIRGSQGSQGPAGPPGPPGPPGPPGPSGGGYEFGFDGDFYRA**

**>Ovis**

**QLSYGYDEKSTG-ISVPGPMGPSGPRGLPGPPGAPGPQGFQGPPGEPGEPGASGPMGPRG**

**PPGPPGKNGDDGEAGKPGRPGERGPPGPQGARGLPGTAGLPGMKGHRGFSGLDGAKGDAG**

**PAGPKGEPGSPGENGTPGQMGPRGLPGERGRPGAPGPAGARGNDGATGAAGPPGPTGPAG**

**PPGFPGAVGAKGEAGPQGPRGSEGPQGVRGEPGPPGPAGAAGPAGNPGADGQPGAKGANG**

**APGIAGAPGFPGARGPSGPQGPSGPPGPKGNSGEPGAPGSKGDTGAKGEPGPTGIQGPPG**

**PAGEEGKRGARGEPGPAGLPGPPGERGGPGSRGFPGADGVAGPKGPAGERGAPGPAGPKG**

**SPGEAGRPGEAGLPGAKGLTGSPGSPGPDGKTGPPGPAGQDGRPGPPGPPGARGQAGVMG**

**FPGPKGAAGEPGKAGERGVPGPPGAVGPAGKDGEAGAQGPPGPAGPAGERGEQGPAGSPG**

**FQGLPGPAGPPGEAGKPGEQGVPGDLGAPGPSGARGERGFPGERGVQGPPGPAGPRGANG**

**APGNDGAKGDAGAPGAPGSQGAPGLQGMPGERGAAGLPGPKGDRGDAGPKGADGAPGKDG**

**VRGLTGPIGPPGPAGAPGDKGETGPSGPAGPTGARGAPGDRGEPGPPGPAGFAGPPGADG**

**QPGAKGEPGDAGAKGDAGPPGPAGPAGPPGPIGNVGAPGPKGARGSAGPPGATGFPGAAG**

**RVGPPGPSGNAGPPGPPGPAGKEGSKGPRGETGPAGRAGEVGPPGPPGPAGEKGAPGADG**

**PAGAPGTPGPQGIAGQRGVVGLPGQRGERGFPGLPGPSGEPGKQGPSGASGERGPPGPMG**

**PPGLAGPPGESGREGAPGAEGSPGRDGAPGAKGDRGETGPAGPPGAPGAPGAPGPVGPAG**

**KSGDRGETGPAGPAGPIGPVGARGPAGPQGPRGDKGETGEQGDRGIKGHRGFSGLQGPPG**

**PPGSPGEQGPSGASGPAGPRGPPGSAGTPGKDGLNGLPGPIGPPGPRGRTGDAGPAGPPG**

**PPGPPGPPGPPSGGYDLSFLPQPPQEKAHDGGRYYRARQFDG-KG-G-GPGPMGLMGPRG**

**PPGASGAPGPQGFQGPPGEPGEPGQTGPAGARGPPGPPGKAGEDGHPGKPGRPGERGVVG**

**PQGARGFPGTPGLPGFKGIRGHNGLDGLKGQPGAPGVKGEPGAPGENGTPGQTGARGLPG**

**ERGRVGAPGPAGARGSDGSVGPVGPAGPIGSAGPPGFPGAPGPKGELGPVGNPGPAGPAG**

**PRGEVGLPGLSGPVGPPGNPGANGLPGAKGAAGLPGVAGAPGLPGPRGIPGPVGAAGATG**

**ARGLVGEPGPAGSKGESGNKGEPGAVGQPGPPGPSGEEGKRGSTGEIGPAGPPGPPGLRG**

**NPGSRGLPGADGRAGVMGPAGSRGATGPAGVRGPNGDSGRPGEPGLMGPRGFPGSPGNIG**

**PAGKEGPAGLPGIDGRPGPIGPAGARGEPGNIGFPGPKGPTGDPGKAGEKGHAGLAGPRG**

**APGPDGNNGAQGPPGLQGVQGGKGEQGPAGPPGFQGLPGPAGTAGEAGKPGERGLPGEFG**

**LPGPAGARGERGPPGESGAAGPTGPIGSRGPSGPPGPDGNKGEPGVVGAPGTAGPSGPSG**

**LPGERGAAGIPGGKGEKGETGLRGDVGSPGRDGARGAPGAVGAPGPAGANGDRGEAGPAG**

**PAGPAGPRGSPGERGEVGPAGPNGFAGPAGAAGQPGAKGERGTKGPKGENGPVGPTGPVG**

**AAGPSGPNGPPGPAGSRGDGGPPGATGFPGAAGRTGPPGPAGISGPPGPPGPAGKEGLRG**

**PRGDQGPVGRTGEPGAAGPPGFVGEKGPSGEPGTAGPPGTPGPQGLLGAPGFLGLPGSRG**

**ERGLPGVAGSVGEPGPLGIAGPPGARGPPGNVGNPGVNGAPGEAGRDGNPGNDGPPGRDG**

**QPGHKGERGYPGNAGPVGAAGAPGPQGPVGPTGKHGSRGEPGPVGAVGPAGAVGPRGPSG**

**PQGIRGDKGEPGDKGPRGLPGLKGHNGLQGLPGLAGHHGDQGAPGAVGPAGPRGPAGPTG**

**PAGKDGRTGQPGAVGPAGIRGSQGSQGPAGPPGPPGPPGPPGPSGGGYDFGFDGDFYRA**

**>Sus**

**QLSYGYDEKSAG-ISVPGPMGPSGPRGLPGPPGAPGPQGFQGPPGEPGEPGASGPMGPRG**

**PPGPPGKNGDDGEAGKPGRPGERGPPGPQGARGLPGPAGLPGMKGHRGFSGLDGAKGDAG**

**PAGPKGEPGSPGENGAPGQMGPRGLPGERGRPGPPGTAGARGNDGATGAAGPPGPTGPAG**

**PPGFPGAVGAKGEAGPQGARGSEGPQGVRGEPGPPGPAGAAGPAGNPGADGQPGAKGANG**

**APGIAGAPGFPGARGPSGPQGPSGPPGPKGNSGEPGAPGSKGDTGAKGEPGPTGVQGPPG**

**PAGEEGKRGARGEPGPAGLPGPPGERGGPGSRGFPGADGVAGPKGPAGERGSPGPAGPKG**

**SPGEAGRPGEAGLPGAKGLTGSPGSPGPDGKTGPPGPAGQDGRPGPPGPPGARGQAGVMG**

**FPGPKGAAGEPGKAGERGVPGPPGAVGPAGKDGEAGAQGPPGPAGPAGERGEQGPAGSPG**

**FQGLPGPAGPPGEAGKPGEQGVPGDLGAPGPSGARGERGFPGERGVQGPPGPAGPRGANG**

**APGNDGAKGDAGAPGAPGSQGAPGLQGMPGERGAAGLPGPKGDRGDAGPKGADGAPGKDG**

**VRGLTGPIGPPGPAGAPGDKGETGPSGPAGPTGARGAPGDRGEPGPPGPAGFAGPPGADG**

**QPGAKGEPGDAGAKGDAGPPGPAGPTGPPGPIGSVGAPGPKGARGSAGPPGATGFPGAAG**

**RVGPPGPSGNAGPPGPPGPAGKEGSKGPRGETGPAGRPGEVGPPGPPGPAGEKGSPGADG**

**PAGAPGTPGPQGIAGQRGVVGLPGQRGERGFPGLPGPSGEPGKQGPSGPSGERGPPGPMG**

**PPGLAGPPGESGREGAPGAEGSPGRDGAPGPKGDRGESGPAGPPGAPGAPGAPGPVGPAG**

**KSGDRGETGPAGPAGPVGPVGARGPAGPQGPRGDKGETGEQGDRGIKGHRGFSGLQGPPG**

**PPGSPGEQGPSGASGPAGPRGPPGSAGAPGKDGLNGLPGPIGPPGPRGRTGDAGPVGPPG**

**PPGPPGPPGPPSGGFDFSFLPQPPQEKAHDGGRYYRARQYDG-KGVGAGPGPMGLMGPRG**

**PPGAVGAPGPQGFQGPAGEPGEPGQTGPAGARGPPGPPGKAGEDGHPGKPGRPGERGVVG**

**PQGARGFPGTPGLPGFKGIRGHNGLDGLKGQPGAPGVKGEPGAPGENGTPGQTGARGLPG**

**ERGRVGAPGPAGARGNDGSVGPVGPAGPIGSAGPPGFPGAPGPKGELGPVGNPGPAGPAG**

**PRGEVGLPGVSGPVGPPGNPGANGLPGAKGAAGLPGVAGAPGLPGPRGIPGPAGAAGATG**

**ARGLVGEPGPAGSKGESGNKGEPGAAGPQGPPGPSGEEGKRGPNGEVGSAGPPGPPGLRG**

**NPGSRGLPGADGRAGVMGPPGSRGPTGPAGVRGPNGDSGRPGEPGLMGPRGFPGSPGNVG**

**PAGKEGPAGLPGIDGRPGPIGPAGARGEPGNIGFPGPKGPTGDPGKNGEKGHAGLAGARG**

**APGPDGNNGAQGPPGPQGVQGGKGEQGPAGPPGFQGLPGPAGTAGEVGKPGERGLPGEFG**

**LPGPAGPRGERGPPGESGAAGPAGPIGSRGPSGPPGPDGNKGEPGVLGAPGTAGPSGPSG**

**LPGERGAAGIPGGKGEKGETGLRGDVGSPGRDGARGAPGAVGAPGPAGANGDRGEAGPAG**

**PAGPAGPRGSPGERGEVGPAGPNGFAGPAGAAGQPGAKGERGTKGPKGENGPVGPTGPVG**

**AAGPAGPNGPPGPAGSRGDGGPPGATGFPGAAGRIGPPGPSGISGPPGPPGPAGKEGLRG**

**PRGDQGPVGRTGETGASGPPGFAGEKGPSGEPGTAGPPGTPGPQGLLGAPGFLGLPGSRG**

**ERGLPGVAGSVGEPGPLGIAGPPGARGPPGAVGNPGVNGAPGEAGRDGNPGSDGPPGRDG**

**QAGHKGERGYPGNPGPAGAAGAPGPQGAVGPAGKHGNRGEPGPAGSVGPAGAVGPRGPSG**

**PQGIRGDKGEPGDKGPRGLPGLKGHNGLQGLPGLAGHHGDQGAPGPVGPAGPRGPAGPSG**

**PAGKDGRTGQPGAVGPAGIRGSQGSQGPAGPPGPPGPPGPPGPSGGGYDFGYEGDFYRA**

**>Canis**

**QMSYGYDEKSTGGISVPGPMGPSGPRGLPGPPGAPGPQGFQGPPGEPGEPGASGPMGPRG**

**PPGPPGKNGDDGEAGKPGRPGERGPPGPQGARGLPGTAGLPGMKGHRGFSGLDGAKGDAG**

**PAGPKGEPGSPGENGAPGQMGPRGLPGERGRPGAPGPAGARGNDGATGAAGPPGPTGPAG**

**PPGFPGAVGAKGEAGPQGARGSEGPQGVRGEPGPPGPAGAAGPAGNPGADGQPGAKGANG**

**APGIAGAPGFPGARGPSGPQGPSGPPGPKGNSGEPGAPGNKGDTGAKGEPGPTGIQGPPG**

**PAGEEGKRGARGEPGPTGLPGPPGERGGPGSRGFPGADGVAGPKGPAGERGSPGPAGPKG**

**SPGEAGRPGEAGLPGAKGLTGSPGSPGPDGKTGPPGPAGQDGRPGPPGPPGARGQAGVMG**

**FPGPKGAAGEPGKAGERGVPGPPGAVGPAGKDGEAGAQGPPGPAGPAGERGEQGPAGSPG**

**FQGLPGPAGPPGEAGKPGEQGVPGDLGAPGPSGARGERGFPGERGVQGPPGPAGPRGANG**

**APGNDGAKGDAGAPGAPGSQGAPGLQGMPGERGAAGLPGPKGDRGDAGPKGADGSPGKDG**

**VRGLTGPIGPPGPAGAPGDKGEAGPSGPAGPTGARGAPGDRGEPGPPGPAGFAGPPGADG**

**QPGAKGEPGDAGAKGDAGPPGPAGPTGPPGPIGNVGAPGPKGARGSAGPPGATGFPGAAG**

**RVGPPGPSGNAGPPGPPGPAGKEGGKGARGETGPAGRPGEVGPPGPPGPAGEKGSPGADG**

**PAGAPGTPGPQGIAGQRGVVGLPGQRGERGFPGLPGPSGEPGKQGPSGTSGERGPPGPMG**

**PPGLAGPPGESGREGAPGAEGSPGRDGSPGPKGDRGETGPAGPPGAPGAPGAPGPVGPAG**

**KNGDRGETGPAGPAGPIGPVGARGPAGPQGPRGDKGETGEQGDRGIKGHRGFSGLQGPPG**

**PPGSPGEQGPSGASGPAGPRGPPGSAGSPGKDGLNGLPGPIGPPGPRGRTGDAGPVGPPG**

**PPGPPGPPGPPSGGFDFSFLPQPPQEKAHDGGRYYRARQYDG-KGVGLGPGPMGLMGPRG**

**PPGASGAPGPQGFQGPAGEPGEPGQTGPAGARGPPGPPGKAGEDGHPGKPGRPGERGVVG**

**PQGARGFPGTPGLPGFKGIRGHNGLDGLKGQPGAPGVKGEPGAPGENGTPGQTGARGLPG**

**ERGRVGAPGPAGARGSDGSVGPVGPAGPIGSAGPPGFPGAPGPKGELGPVGNPGPAGPAG**

**PRGEVGLPGVSGPVGPPGNPGANGLTGAKGAAGLPGVAGAPGLPGPRGIPGPVGAAGATG**

**ARGLVGEPGPAGSKGESGNKGEPGSAGAQGPPGPSGEEGKRGPNGEAGSAGPSGPPGLRG**

**SPGSRGLPGADGPAGVMGPPGPRGATGPAGVRGPNGDSGRPGEPGLMGPRGFPGAPGNVG**

**PAGKEGPMGLPGIDGRPGPIGPAGARGEPGNIGFPGPKGPTGDPGKNGDKGHAGLAGARG**

**APGPDGNNGAQGPPGPQGVQGGKGEQGPAGPPGFQGLPGPAGTAGEVGKPGERGLPGEFG**

**LPGPAGPRGERGPPGESGAAGPSGPIGSRGPSGPPGPDGNKGEPGVLGAPGTAGASGPGG**

**LPGERGAAGIPGGKGEKGETGLRGEIGNPGRDGARGAPGAMGAPGPAGATGDRGEAGPAG**

**PAGPAGPRGTPGERGEVGPAGPNGFAGPAGAAGQPGAKGERGTKGPKGENGPVGPTGPIG**

**SAGPSGPNGPPGPAGSRGDGGPPGATGFPGAAGRTGPPGPSGITGPPGPPGAAGKEGLRG**

**PRGDQGPVGRTGETGASGPPGFTGEKGPSGEPGTAGPPGTPGPQGLLGAPGILGLPGSRG**

**ERGLPGVAGSVGEPGPLGIAGPPGARGPPGAVGAPGVNGAPGEAGRDGNPGNDGPPGRDG**

**QAGHKGERGYPGNIGPVGAVGAPGPHGPVGPTGKHGNRGEPGPAGSVGPVGAVGPRGPSG**

**PQGIRGDKGEPGEKGPRGLPGLKGHNGLQGLPGLAGQHGDQGAPGSVGPAGPRGPAGPSG**

**PAGKDGRTGQPGTVGPAGIRGSQGSQGPAGPPGPPGPPGPPGPSGGGYDFGYEGDFYRA**

**>Ailuropoda**

**QMSYGYDEKSTGGISVPGPMGPSGPRGLPGPPGAPGPQGFQGPPGEPGEPGASGPMGPRG**

**PPGPPGKNGDDGEAGKPGRPGERGPPGPQGARGLPGTAGLPGMKGHRGFSGLDGAKGDAG**

**PAGPKGEPGSPGENGAPGQMGPRGLPGERGRPGAPGPAGARGNDGATGAAGPPGPTGPAG**

**PPGFPGAVGAKGEAGPQGARGSEGPQGVRGEPGPPGPAGAAGPAGNPGADGQPGAKGANG**

**APGIAGAPGFPGARGPSGPQGPSGPPGPKGNSGEPGAPGNKGDTGAKGEPGPTGIQGPPG**

**PAGEEGKRGARGEPGPTGLPGPPGERGGPGSRGFPGADGVAGPKGPAGERGSPGPAGPKG**

**SPGEAGRPGEAGLPGAKGLTGSPGSPGPDGKTGPPGPAGQDGRPGPPGPPGARGQAGVMG**

**FPGPKGAAGEPGKAGERGVPGPPGAVGPAGKDGEAGAQGPPGPAGPAGERGEQGPAGSPG**

**FQGLPGPAGPPGEAGKPGEQGVPGDLGAPGPSGARGERGFPGERGVQGPPGPAGPRGANG**

**APGNDGAKGDAGAPGAPGSQGAPGLQGMPGERGAAGLPGPKGDRGDAGPKGADGSPGKDG**

**VRGLTGPIGPPGPAGAPGDKGEAGPSGPAGPTGARGAPGDRGEPGPPGPAGFAGPPGADG**

**QPGAKGEPGDAGAKGDAGPPGPAGPTGPPGPIGNVGAPGPKGARGSAGPPGATGFPGAAG**

**RVGPPGPSGNAGPPGPPGPAGKEGGKGPRGETGPAGRPGEVGPPGPPGPAGEKGSPGADG**

**PAGAPGTPGPQGIAGQRGVVGLPGQRGERGFPGLPGPSGEPGKQGPSGASGERGPPGPMG**

**PPGLAGPPGESGREGSPGAEGSPGRDGSPGPKGDRGETGPAGPPGAPGAPGAPGPVGPAG**

**KSGDRGETGPAGPAGPIGPVGARGPAGPQGPRGDKGETGEQGDRGIKGHRGFSGLQGPPG**

**PPGSPGEQGPSGASGPAGPRGPPGSAGSPGKDGLNGLPGPIGPPGPRGRTGDAGPVGPPG**

**PPGPPGPPGPPSGGFDFSFLPQPPQEKAHDGGRYYRARQYDG-KGVGLGPGPMGLMGPRG**

**PPGASGAPGPQGFQGPAGEPGEPGQTGPAGARGPPGPPGKAGEDGHPGKPGRPGERGVVG**

**PQGARGFPGTPGLPGFKGIRGHNGLDGLKGQPGAPGVKGEPGAPGENGTPGQTGARGLPG**

**ERGRVGAPGPAGARGSDGSVGPVGPAGPIGSAGPPGFPGAPGPKGELGPVGNPGPAGPAG**

**PRGEVGLPGVSGPVGPPGNPGANGLTGAKGAAGLPGVAGAPGLPGPRGIPGPVGAAGATG**

**ARGLVGEPGPAGSKGESGNKGEPGSVGPQGPPGPSGEEGKRGPNGEAGSAGPSGPPGLRG**

**SPGSRGLPGADGRAGVMGPPGPRGSTGPAGVRGPNGDSGRPGEPGLMGPRGFPGAPGNVG**

**PAGKEGPMGLPGIDGRPGPIGPAGARGEPGNIGFPGPKGPSGEPGKAGEKGHAGLAGARG**

**APGPDGNNGAQGPPGPQGVQGGKGEQGPAGPPGFQGLPGPAGTAGEVGKPGERGLPGEFG**

**LPGPAGPRGERGPPGESGAAGPSGPIGSRGPSGPPGPDGNKGEPGVLGAPGTAGPSGPGG**

**LPGERGAAGVPGGKGEKGETGLRGEVGNPGRDGARGAPGAVGAPGPAGATGDRGEAGPAG**

**PAGPAGPRGSPGERGEVGPAGPNGFAGPAGAAGQPGAKGERGTKGPKGENGPVGPTGPVG**

**SAGPSGPNGPPGPAGSRGDGGPPGATGFPGAAGRTGPPGPSGITGPPGPPGAAGKEGLRG**

**PRGDQGPVGRTGETGAHGPPGFAGEKGPSGEPGTAGPPGTAGPQGLLGAPGILGLPGSRG**

**ERGLPGVSGSVGEPGPLGIAGPPGARGPPGAVGAPGVNGAPGEAGRDGNPGNDGPPGRDG**

**QPGHKGERGYPGNIGPVGTVGAPGPHGPVGPTGKHGNRGEPGPAGAVGPVGAVGPRGPSG**

**PQGVRGDKGEPGDKGPRGLPGLKGHNGLQGLPGLAGQHGDQGAPGSVGPAGPRGPAGPSG**

**PAGKDGRTGHPGTVGPAGVRGSQGSQGPAGPPGPPGPPGPPGPSGGGYDFGYEGDFYRA**

**>Felis**

**QMSYGYDEKSTGGISVPGPMGPSGPRGLPGPPGAPGPQGFQGPPGEPGEPGASGPMGPRG**

**PPGPPGKNGDDGEAGKPGRPGERGPPGPQGARGLPGTAGLPGMKGHRGFSGLDGAKGDAG**

**PAGPKGEPGSPGENGAPGQMGPRGLPGERGRPGAPGPAGARGNDGATGAAGPPGPTGPAG**

**PPGFPGAVGAKGEAGPQGARGSEGPQGVRGEPGPPGPAGAAGPAGNPGADGQPGAKGANG**

**APGIAGAPGFPGARGPSGPQGPSGPPGPKGNSGEPGAPGNKGDTGAKGEPGPTGIQGPPG**

**PAGEEGKRGARGEPGPTGLPGPPGERGGPGSRGFPGADGVAGPKGPAGERGSPGPAGPKG**

**SPGEAGRPGEAGLPGAKGLTGSPGSPGPDGKTGPPGPAGQDGRPGPPGPPGARGQAGVMG**

**FPGPKGAAGEPGKAGERGVPGPPGAVGPAGKDGEAGAQGPPGPAGPAGERGEQGPAGSPG**

**FQGLPGPAGPPGEAGKPGEQGVPGDLGAPGPSGARGERGFPGERGVQGPPGPAGPRGANG**

**APGNDGAKGDAGAPGAPGSQGAPGLQGMPGERGAAGLPGPKGDRGDAGPKGADGSPGKDG**

**VRGLTGPIGPPGPAGAPGDKGEAGPSGPAGPTGARGAPGDRGEPGPPGPAGFAGPPGADG**

**QPGAKGEPGDAGAKGDAGPPGPAGPTGPPGPIGNVGAPGPKGARGSAGPPGATGFPGAAG**

**RVGPPGPSGNAGPPGPPGPVGKEGGKGPRGETGPAGRPGEVGPPGPPGPAGEKGSPGADG**

**PAGAPGTPGPQGIAGQRGVVGLPGQRGERGFPGLPGPSGEPGKQGPSGPSGERGPPGPMG**

**PPGLAGPPGESGREGSPGAEGSPGRDGSPGPKGDRGETGPAGPPGAPGAPGAPGPVGPAG**

**KSGDRGETGPAGPAGPIGPVGARGPAGPQGPRGDKGETGEQGDRGIKGHRGFSGLQGPPG**

**PPGSPGEQGPSGASGPAGPRGPPGAAGSPGKDGLNGLPGPIGPPGPRGRTGDAGPVGPPG**

**PPGPPGPPGPPSGGFDFSFLPQPPQEKAHDGGRYYRARQYDPGKGVGLGPGPMGLMGPRG**

**PPGASGAPGPQGFQGPAGEPGEPGQTGPAGARGPPGPPGKAGEDGHPGKPGRPGERGVVG**

**PQGARGFPGTPGLPGFKGIRGHNGLDGLKGQPGAPGVKGEPGAPGENGTPGQTGARGLPG**

**ERGRVGAPGPAGARGSDGSVGPVGPAGPIGSAGPPGFPGAPGPKGELGPVGNPGPAGPAG**

**PRGEMGLPGVSGPVGPPGNPGANGLTGAKGAAGLPGVAGAPGLPGPRGIPGPVGAAGATG**

**ARGLVGEPGPAGSKGESGNKGEPGSAGPQGPPGPSGEEGKRGPNGEAGSAGPSGPPGLRG**

**SPGSRGLPGADGRAGVMGPPGPRGATGPAGVRGPNGDAGRPGEPGLMGPRGFPGAPGNVG**

**PAGKEGPMGLPGIDGRPGPIGPAGARGEPGNIGFPGPKGPTGDPGKNGDKGHAGLAGARG**

**APGPDGNNGAQGPPGPQGVQGGKGEQGPAGPPGFQGLPGPAGTAGEVGKPGERGLPGEFG**

**LPGPAGPRGERGPPGESGAAGPSGPIGSRGPSGPPGPDGNKGEPGVLGAPGTAGPSGPSG**

**LPGERGAAGIPGGKGEKGETGLRGEIGNPGRDGARGAPGAVGAPGPAGATGDRGEAGPAG**

**PAGPAGPRGSPGERGEVGPAGPNGFAGPAGAAGQPGAKGERGTKGPKGENGPVGPTGPVG**

**SAGPSGPNGPPGPAGSRGDGGPPGATGFPGAAGRTGPPGPSGITGPPGPPGAAGKEGLRG**

**PRGDQGPVGRTGETGASGPPGFAGEKGPSGEPGTAGPPGTPGPQGLLGAPGILGLPGSRG**

**ERGLPGVSGSVGEPGPLGISGPPGARGPSGAVGAPGVNGAPGEAGRDGNPGNDGPPGRDG**

**QPGHKGERGYPGNIGPVGAVGAPGPHGPVGPTGKHGNRGEPGPAGVVGPVGAVGPRGPTG**

**PQGIRGDKGEPGDKGPRGLPGLKGHNGLQGLPGLAGQHGDQGAPGSVGPAGPRGPAGPSG**

**PMGKDGRTGHPGSVGPAGVRGSQGSQGPAGPPGPPGPPGPPGPSGGGYDFGYEGDFYRA**

**>Ornithorhynchus**

**QMAYGYDEKAGGGMSVPGPMGPSGPRGLPGPPGSPXXXXXXXXXXXXXXXXXXGPMGPRG**

**PAGPPGKNGDDGEAGKPGRPGERGPPGPQGARGLPGTAGLPGMKGHRGFSGLDGAKGDSG**

**PAGPKGEPGSAGENGAPGQMGPRGLPGERGRPGPSGPAGARGNDGAPGAAGPPGPTGPAG**

**PPGFPGAVGAKGEAGAQGSRGSEGPQGARGEPGPPGPAGAAGPSGNPGSDGQPGAKGANG**

**APGIAGAPGFPGARGPSGPQGPSGGPGPKGNSGEPGAPGNKGDPGAKGEPGPVGVQGPPG**

**PSGEEGKRGSRGEPGPTGLPGPAGERGGPGSRGFPGADGVAGPKGPAGERGSPGPAGPKG**

**SPGEAGRPGEAGLPGAKXXXXXXXXXXXXXXXXXXXXXXXXXXXXXXXXXXXXXXXXXXX**

**XXXXXXXXGEPGKPGERGVPGPPGAVGAAGKDGEAGAQGPPGPAGPAGERGEQGPSGSPG**

**FQGLPGPSGPAGESGKPGEQGVPGDAGAPGPSGARGERGFPGERGVQGPAGPQGPRGSNG**

**APGNDGAKGDAGAPGAPGGQGPPGLQGMPGERGAAGLPGAKGDRGDAGPKGGDGAPGKDG**

**IRGLTGPIGPPGPAGNPGDKGESGPSGPAGPTGARGAPGDRGEPGPPGPAGFAGPPGADG**

**QPGAKGETGDSGAKGDAGPPGPAGPTGAPGPAGNVGAPGPKGARGSAGPPGATGFPGAAG**

**RVGPPGPSXXXXXXXXXXXXXXXXXXXXXXXXXXXXXXXXXXXXXXXXXXXXXXXXXXXX**

**XXXXXXXXXXXXXXXXXXXXXXXXXXXXXXXXXXXXXXXXXXXXXXXXXXXXXXXXXXXX**

**XXXXXXXXXXXXXXXXXXXXXXXXXXXXXXXXGDRGETGPAGPPGAPGAPGAPGPVGPAG**

**KNGDRGETGPSGPAGPAGPAGARGPSGPQGPRGDKGETGEQGDRGMKGHRGFSGLQGPPG**

**PPGSPGEQGPSGASGPAGPRXXXXXXXXXXXXXXXXXXXXXXXXXXXXXXXXXXXXXXXX**

**XXXXXXXXXXXXXXXXXXXXXXXXXXXXXXXXXXXXXXQYDGSKAADMGPGPMGLMGPRG**

**PPGASGAPGAQGFQGPPGEPGEPGQSGPAGSRGPAGPPGKSGEDGHPGKPGRSGERGVVG**

**PQGARGFPGTPGLPGFKGIRGHNGLDGQKGQPGTPGVKGEPGAPGENGSPGQSGARGLPG**

**ERGRIGGAGPTGARGSDGSVGPVGPAGPIGSAGPPGFPGAPGPKGELGAVGNTGPAGPAG**

**PRGELGLPGVSGPVGPAGNPGANGLAGAKGAAGLPGVAGAPGLPGPRGIPGPSGPSGPSG**

**PRGLVGEPGPAGSKGESGSKGEPGSAGAQGPPGPNGEEGKRGPNGEPGSTGPTGPPGLRG**

**VPGSRGLPGADGRAGGMGPAGNRGSAGPSGARGPSGDSGRPGEPGLVGPRGLPGFPGNVG**

**PAGKEGPVGLPGSEGRPGPTGPAGARGEPGNIGFPGPKGPNGEPGKSGERGHAGLAGSRG**

**APGPDGNNGAXXXXXXXXXXXXXXXXXXXXXXXXXXXXXXXXXXXXXXXXXXXGLPGEFG**

**LPGPAGPRGERGPPGESGAAGPTGPIGNRGPSGPPGPDGNKGEPGVAGAPGNAGPAGSGG**

**LPGERGAVGVPGGKGEKGEPGLRGEFGNPGRDGARGAPGAVGSPGPSGATGDRGEAGAAG**

**PAGPAGPRGSPGERGEVGPAGPNGFAGPPGAAGQAGAKGERGTKGPKGENGPTGPVGAVG**

**SAGPAGPNGLPGPTGGRGDGGPPGMTGFPGAAGRTGPAGPSGITGPSGPPGASGKEGPRG**

**PRGDQGPVGRTGELGAVGPPGFTGEKGPSGEPGTAGPPGTPGPQGLLGSPGILGLPGSRG**

**ERGLPGVSGGLGEPGPLGISGPSGARGPPGNVGNPGVNGAPGEAGRDXXXXXXXXXXXXX**

**XXXXXXXXXXXXXXXXXXXXXXXXXXXXXXXXXXXXXXXXXXXXXXXXXXXXXXXXXXXX**

**XXXXXXXXXXXXXXXXXXXXXXXXXXXXXXXXXXXXXXXXXXXXXXXXXXXXXXXXXXXX**

**XXXXXXXXXXXXXXXXXXXXXXXXXXXXXXXXXXXXXXXXXXXXXXXXXXXXXXXXXXX**

**>Pongo**

**QLSYGYDEKSTGGISVPGPMGPSGPRGLPGPPGAPGPQGFQGPPGEPGEPGASGPMGPRG**

**PPGPPGKNGDDGEAGKPGRPGERGPPGPQGARGLPGTAGLPGMKGHRGFSGLDGAKGDAG**

**PAGPKGEPGSPGENGAPGQMGPRGLPGERGRPGAPGPAGARGNDGATGAAGPPGPTGPAG**

**PPGFPGAVGAKGEAGPQGPRGSEGPQGVRGEPGPPGPAGAAGPAGNPGADGQPGAKGANG**

**APGIAGAPGFPGARGPSGPQGPGGPPGPKGNSGEPGAPGSKGDTGAKGEPGPVGVQGPPG**

**PAGEEGKRGARGEPGPTGLPGPPGERGGPGSRGFPGADGVAGPKGPAGERGSPGPAGPKG**

**SPGEAGRPGEAGLPGAKGLTGSPGSPGPDGKTGPPGPAGQDGRPGPPGPPGARGQAGVMG**

**FPGPKGAAGEPGKAGERGVPGPPGAVGPAGKDGEAGAQGPPGPAGPAGERGEQGPAGSPG**

**FQGLPGPAGPPGEAGKPGEQGVPGDLGAPGPSGARGERGFPGERGVQGPPGPAGPRGANG**

**APGNDGAKGDAGAPGAPGSQGAPGLQGMPGERGAAGLPGPKGDRGDAGPKGADGSPGKDG**

**VRGLTGPIGPPGPAGAPGDKGETGPSGPAGPTGARGAPGDRGEPGPPGPAGFAGPPGADG**

**QPGAKGEPGDAGAKGDAGPPGPAGPAGPPGPIGNVGAPGAKGARGSAGPPGATGFPGAAG**

**RVGPPGPSGNAGPPGPPGPAGKEGGKGPRGETGPAGRPGEVGPPGPPGPAGEKGSPGADG**

**PAGAPGTPGPQGIAGQRGVVGLPGQRGERGFPGLPGPSGEPGKQGPSGASGERGPPGPMG**

**PPGLAGPPGESGREGAPGAEGSPGRDGSPGAKGDRGETGPAGPPGAPGAPGAPGPVGPAG**

**KSGDRGETGPAGPAGPVGPVGARGPAGPQGPRGDKGETGEQGDRGIKGHRGFSGLQGPPG**

**PPXXXXXXXXXXXXXXXXXXGPPGSAGAPGKDGLNGLPGPIGPPGPRGRTGDAGPVGPPG**

**PPGPPGPPGPPSGGFDFSFLPQPPQEKAHDGGRYYRARQYDG-KGVGLGPGPMGLMXPRG**

**PPGAAGAPGPQGFQGPAGEPGEPGQTGPAGARGPAGPPGKAGEDGHPGKPGRPGERGVVG**

**PQGARGFPGTPGLPGFKGIRGHNGLDGLKGQPGAPGVKGEPGAPGENGTPGQTGARGLPG**

**ERGRVGAPGPAGARGSDGSVGPVGPAGPIGSAGPPGFPGAPGPKGELGAVGNAGPAGPAG**

**PRGEVGLPGLSGPVGPPGNPGANGLTGAKGAAGLPGVAGAPGLPGPRGIPGPVGAAGATG**

**ARGLVGEPGPAGSKGESGNKGEPGSAGPQGPPGPSGEEGKRGPNGEAGSAGPPGPPGLRG**

**SPGSRGLPGADGRAGVMGPPGSRGASGPAGVRGPSGDAGRPGEPGLMGPRGLPGSPGNIG**

**PAGKEGPVGLPGIDGRPGPIGPAGARGEPGNIGFPGPKGPTGDPGKNGDKGHAGLAGARG**

**APGPDGNNGAQGPPGPQGVQGGKGEQGPAGPPGFQGLPGPSGPAGEVGKPGERGLHGEFG**

**LPGPAGPRGERGPPGESGAAGPTGPIGSRGPSGPPGPDGNKGEPGVVGAVGTAGPSGPSG**

**LPGERGAAGIPGGKGEKGEPGLRGEIGNPGRDGARGAPGAVGAPGPAGATGDRGEAGAAG**

**PAGPAGPRGSPGERGEVGPAGPNGFAGPAGAAGQPGAKGERGTKGPKGENGVVGPTGPVG**

**AAGPAGPNGPPGPAGSRGDGGPPGMTGFPGAAGRTGPPGPSGISGPPGPPGPAGKEGLRG**

**PRGDQGPVGRTGEVGAVGPPGFAGEKGPSGEAGTAGPPGTPGPQGLLGAPGILGLPGSRG**

**ERGLPGVAGAVGEPGPLGIAGPPGARGPPGAVGSPGVNGAPGEAGRDGNPGNDGPPGRDG**

**QPGHKGERGYPGNIGPVGAAGAPGPHGPVGPAGKHGNRGETGPSGPVGPVGAVGPRGPSG**

**PQGIRGDKGEPGEKGPRGLPGLKGHNGLQGLPGLAGHHGDQGAPGSVGPAGPRGPAGPSG**

**PAGKDGRTGHPGTVGPAGIRGPQGHQGPAGPPGPPGPPGPPGVSGGGYDFGYDGDFYRA**

**>Gorilla**

**QLSYGYDEKSAGGISVPGPMGPSGPRGLPGPPGAPGPQGFQGPPGEPGEPGASGPMGPRG**

**PPGPPGKNGDDGEAGKPGRPGERGPPGPQGARGLPGTAGLPGMKGHRGFSGLDGAKGDAG**

**PAGPKGEPGSPGENGAPGQMGPRGLPGERGRPGAPGPAGARGNDGATGAAGPPGPTGPAG**

**PPGFPGAVGAKGEAGPQGPRGSEGPQGVRGEPGPPGPAGAAGPAGNPGADGQPGAKGANG**

**APGIAGAPGFPGARGPSGPQGPGGPPGPKGNSGEPGAPGSKGDTGAKGEPGPVGVQGPPG**

**PAGEEGKRGARGEPGPTGLPGPPGERGGPGSRGFPGADGVAGPKGPAGERGSPGPAGPKG**

**SPGEAGRPGEAGLPGAKGLTGSPGSPGPDGKTGPPGPAGQDGRPGPPGPPGARGQAGVMG**

**FPGPKGAAGEPGKAGERGVPGPPGAVGPAGKDGEAGAQGPPGPAGPAGERGEQGPAGSPG**

**FQGLPGPAGPPGEAGKPGEQGVPGDLGAPGPSGARGERGFPGERGVQGPPGPAGPRGANG**

**APGNDGAKGDAGAPGAPGSQGAPGLQGMPGERGAAGLPGPKGDRGDAGPKGADGSPGKDG**

**VRGLTGPIGPPGPAGAPGDKGESGPSGPAGPTGARGAPGDRGEPGPPGPAGFAGPPGADG**

**QPGAKGEPGDAGAKGDAGPPGPAGPAGPPGPIGNVGAPGAKGARGSAGPPGATGFPGAAG**

**RVGPPGPSGNAGPPGPPGPAGKEGGKGPRGETGPAGRPGEVGPPGPPGPAGEKGSPGADG**

**PAGAPGTPGPQGIAGQRGVVGLPGQRGERGFPGLPGPSGEPGKQGPSGASGERGPPGPMG**

**PPGLAGPPGESGREGAPGAEGSPGRDGSPGAKGDRGETGPAGPPGAPGAPGAPGPVGPAG**

**KSGDRGETGPAGPAGPVGPVGARGPAGPQGPRGDKGETGEQGDRGIKGHRGFSGLQGPPG**

**PPGSPGEQGPSGASGPAGPRGPPGSAGAPGKDGLNGLPGPIGPPGPRGRTGDAGPVGPPG**

**PPGPPGPPGPPSGGFDFSFLPQPPQEKAHDGGRYYRARQYDG-KGVGLGPGPMGLMGPRG**

**PPGAAGAPGPQGFQGPAGEPGEPGQTGPAGARGPAGPPGKAGEDGHPGKPGRPGERGVVG**

**PQGARGFPGTPGLPGFKGIRGHNGLDGLKGQPGAPGVKGEPGAPGENGTPGQTGARGLPG**

**ERGRVGAPGPAGARGSDGSVGPVGPAGPIGSAGPPGFPGAPGPKGELGAVGNAGPAGPSG**

**PRGEVGLPGLSGPVGPPGNPGANGLTGAKGAAGLPGVAGAPGLPGPRGIPGPVGAAGATG**

**ARGLVGEPGPAGSKGESGNKGEPGSAGPQGPPGPSGEEGKRGPNGEAGSAGPPGPPGLRG**

**SPGSRGLPGADGRAGVMGPPGSRGASGPAGVRGPNGDAGRPGEPGLMGPRGLPGSPGNIG**

**PAGKEGPVGLPGIDGRPGPIGPAGARGEPGNIGFPGPKGPTGDPGKNGDKGHAGLAGARG**

**APGPDGNNGAQGPPGPQGVQGGKGEQGPAGPPGFQGLPGPSGPAGEVGKPGERGLHGEFG**

**LPGPAGPRGERGPPGESGAAGPTGPIGSRGPSGPPGPDGNKGEPGVVGAVGTAGPSGPSG**

**LPGERGAAGIPGGKGEKGEPGLRGEIGNPGRDGARGAPGAVGAPGPAGATGDRGEAGAAG**

**PAGPAGPRGSPGERGEVGPAGPNGFAGPAGAAGQPGAKGERGAKGPKGENGVVGPTGPVG**

**AAGPAGPNGPPGPAGSRGDGGPPGMTGFPGAAGRTGPPGPSGISGPPGPPGPAGKEGLRG**

**PRGDQGPVGRTGEVGAVGPPGFAGEKGPSGEAGTAGPPGTPGPQGLLGAPGILGLPGSRG**

**ERGLPGVAGAVGESGPLGIAGPPGARGPPGAVGSPGVNGAPGEAGRDGNPGNDGPPGRDG**

**QPGHKGERGYPGNIGPVGAAGAPGPHGPVGPAGKHGNRGETGPSGPVGPAGAVGPRGPSG**

**PQGIRGDKGEPGEKGPRGLPGLKGHNGLQGLPGLAGHHGDQGASGSVGPAGPRGPAGPSG**

**PAGKDGRTGHPGTVGPAGIRGPQGHQGPAGPPGPPGPPGPPGVSGGGYDFGYDGDFYRA**

**>Saimiri**

**QLSYGYDEKSTGGISVPGPMXXXXXXXXXXXXXXXGPQGFQGPPGEPGEPGASGPMGPRG**

**PPGPPGKNGDDGEAGKPGRPGERGPPGPQGARGLPGTAGLPGMKGHRGFSGLDGAKGDAG**

**PAGPKGEPGSPGENGAPGQMGPRGLPGERGRPGAPGPAGARGNDGATGAAGPPGPTGPAG**

**PAGFPGAVGAKGEAGPQGPRGSEGPQGVRGEPGPPGPAGAAGPAGNPGADGQPGAKGANG**

**APGIAGAPGFPGARGPSGPQGPSGPPGPKGNSGEPGAPGSKGDTGAKGEPGPVGVQGPPG**

**PAGEEGKRGARGEPGPTGLPGPPGERGGPGSRGFPGADGVAGPKGPAGERGSPGPAGPKG**

**SPGEAGRPGEAGLPGAKGLTGSPGSPGPDGKTGPPGPAGQDGRPGPPGPPGARGQAGVMG**

**FPGPKGAAGEPGKAGERGVPGPPGAVGPAGKDGEAGAQGPPGPAGPAGERGEQGPAGSPG**

**FQGLPGPAGPPGEAGKPGEQGVPGDLGAPGPSGARGERGFPGERGVQGPPGPAGPRGANG**

**APGNDGAKGDAGAPGAPGSQGAPGLQGMPGERGAAGLPGPKGDRGDAGPKGADGSPGKDG**

**VRGLTGPIGPPGPAGAPGDKGETGPSGPAGPTGARGAPGDRGEPGPPGPAGFAGPPGADG**

**QPGAKGEPGDAGAKGDAGPPGPAGPAGPPGPIGNVGAPGPKGARGSAGPPGATGFPGAAG**

**RVGPPGPSGNAGPPGPPGPAGKEGGKGPRGETGPAGRPGEVGPPGPPGPAGEKGSPGADG**

**PAGAPGTPGPQGIAGQRGVVGLPGQRGERGFPGLPGPSGEPGKQGPSGASGERGPPGPMG**

**PPGLAGPPGESGREGAPGAEGSPGRDGSPGPKGDRGETGPAGPPGAPGAPGAPGPVGPAG**

**KSGDRGETGPAGPAGPVGPVGARGPAGPQGPRGDKGETGEQGDRGIKGHRGFSGLQGPPG**

**PPGSPGEQGPSGASGPAGPRGPPGSAGAPGKDGLNGLPGPIGPPGPRGRTGDAGPVGPPG**

**PPGPPGPPGPPSGGFDFSFLPQPPQEKAHDGGRYYRARXXXX?XX?X?XXXXXXXXXXXX**

**XXXXXXXXGPQGFQGPAGEPGEPGQTXXXXXXXXXXXXXXXXXXGHPGKPGRPGERGVVG**

**PQGARGFPGTPGLPGFKGIRGHNGLDGLKGQPGAPGVKGEPGAPGENGTPGQXGARGLPG**

**ERGRVGAPGPAGARGSDGSVGPVGPAGPIGSAGPPGFPGAPGPKGELGAIGNPGAAGPAG**

**PRGEVGLPGLSGPVGPPGNPGANGLTGAKGAXGLPGVAGAPGLPGPRGIPGPVGAAGATG**

**ARGLVXXXXXXXXXXXXXXXXXXGSAGPQGPPGPSGEEGKRGPNGEAGSAGPPGPPGLRG**

**SPGSRGLPGADGRAGVMGPAGSRGASGPAGVRGPSGDAGRPGEPGLMGPRXXXXXXXXXX**

**XXXXXXXXGLPGIDGRPGPIGPAGARGEPGSIGFPGPKGPXXXXXXXXXXXXXXXXXXXG**

**APGPDGNNGAQGPPGPQGVQGGKGEQGPAGPPGFQXXXXXXXXXXXXXXXXXXXXXXXXX**

**XXXXXXXXGERGPPGESGAAGPAGPIGSRGPSGPPGPDGNKGEPGVVGAAGTAGPSGPGG**

**LPGERGAAGIPGGKGEKGEPGLRGEIGNPGRDGARXXXXXXXXXXXXXXXXXXXXXXXXX**

**XXXXXXXXXXXGERGEVGPAGPNGFAGPAGAAGQPGAKGERGAKGPKGENGVVGPTGPVG**

**AAGPSGPNGPPGPAGSRGDGGPPGMTGFPGAAGRTGPPGPSGISGPPGPPGPAGKEGLRG**

**PRGDQGPVGRSGETGAVGPPGFAGEKGPSGEAGTXGPPGTPGPQGLLGAPGILGLPGSRG**

**ERGLPGVAGALGEPGPLGIAGPPGARGPPGAVGSPGVNGAPGEAGRDGNPGNDGPPGRDG**

**QPGHKGERGYPGNIGPVGAAGAPGPHGPVGPAGKHGNRGEXXXXXXXXXXXXXXXXXXXG**

**PQGIRGDKGEPGDKGPRGLPGLKGHNGLQGLPGLAXXXXXXXXXXXXXXXXPXGPAGPSG**

**PAGKDGRTGHPGTVGPAGIRGPQGHQGPAGPPGPPGPPGPPGVSGGGYDFGYDGDFFRA**

**>Cricetulus**

**QMSYGYDEKSAG-VSVPGPMGPSGPRGLPGPPGAPGPQGFQGPPGEPGEPGASGPMGPRG**

**PPGPPGKNGDDXXXXXXXXXXXXXXXXXQGARGLPGTAGLPGMKGHRGFSGLDGAKGDAG**

**PAGPKGEPGSPGENGAPGQMGPRGLPGERGRPGAPGPAXXXXXXXXXXXXXXXXXXXXXX**

**XXXXXXXXXXXGEAGPQGARGSEGPQGVRGEPGPPGPAGAAGPAGNPGADGQPGAKGANG**

**APGIAGAPGFPGARGPSGPQGPSGAPGPKGNSGEPGAPGNKGDTGAKGEPGPAGVQGPPG**

**PAGEEGKRGARGEPGPTGLPGPPGERGGPGSRGFPGADGVAGPKGPAGERGSPGPAGPKG**

**SPGEAGRPGEAGLPGAKGLTGSPGSPGPDGKTGPPGPAGQDGRPGPPGPPGARGQAGVMG**

**FPGPKGTAGEPGKAGERGVPGPPGPVGPAGKDGEAGAQGAPGPAGPAGERGEQGPAGSPG**

**FQGLPGPAGPPGEAGKPGEQGVPGDLGAPGPSGARGERGFPGERGVQGPPGPAGPRGNNG**

**APGNDGAKGDTGAPGAPGSQGAPGLQGMPGERGAAGLPGPKGDRGDAGPKGADGSPGKDG**

**VRGLTGPIGPPGPAGAPGDKGETGPSGPAGPTGARGAPGDRGEPGPPGPAGFAGPPGADG**

**QPGAKGEPGETGTKGDSGPPGPAGPAGPPGPIXXXXXXXXXXXXXXXXXXGATGFPGAAG**

**RVGPPGPSGNAGPPGPPGPVGKEGGKGPRGETGPAGRPGEVGPPGPPGPAGEKGAPGADG**

**PAGSPGTPGPQGIAGQRGVVGLPGQRGERGFPGLPGPSGEPGKQGPSGSSGERGPPGPMG**

**PPGLAGPPGESGREGSPGAEGSPGRDGAPGPKGDRGETGPAGPPGAPGAPGAPGPVGPAG**

**KSGDRGETGPAGPAGPIGPAGARGPAGPQGPRGDKGETGEQGDRGIKGHRGFSGLQGPPG**

**SPGSPGEQGPSGASGPAGPRGPPGSAGAPGKDGLNGLPGPIGPPGPRGRTGDSGPAGPPG**

**PPGPPGPPGPPSGGYDFSFLPQPPQEKSHDGGRYYRARXXXX?XX?X?XXXXXGLMGPRG**

**PPGAVGAPGPQGFQGPAGEPGEPGQTGPAGSRGPAGPPGKAGEDGHPGKAGRPGERGVVG**

**PQGARGFPGTPGLPGFKGIRGHNGLDGLKGQPGAQGVKGEPGAPGENGTPGQAGARGLPG**

**ERGRVGAPGPAGARGSDGSVGPVGPAGPIGSAGPPGFPGAPGPKGELGPVGNPGPSGPAG**

**PRGEVGLPGLSGPVGPPGNPGANGLTGAKGAXGLPGVAGAPGLPGPXXXXXXXXXXXXXX**

**XXXXXGEPGPAGSKGETGNKGEPGSAGAQGPPGPSGEEGKRGSPGEPGSAGPGGPPGLRG**

**SPGSRGLPGADGRAGVMGPPGNRGSTGPAGGRGPNGDPGRPGEPGLMGPRGLPGSPGNVG**

**PSGKEGPVGLPGIDGRPGPIGPAGARGEAGNIGFPGPKGPSXXXXXXXXXXXXXXXXXXG**

**APGPDGNNGAQGPPGPQGVQGGKGEQGPAGPPGFQGLPGPSGTAGEVGKPGERGLPGEFG**

**LPGPAGPRGERGPPGESGAAGPSGPIGSRGPSGAPGPDGNKGEAGAVGAPGNAGASGPGG**

**LPGERGAAGVPGGKGEKGETGLRGEIGNPGRDGARXXXXXXXXXXXXXXXXXXGEAGAAG**

**PSGPAGPRGSPGERGEVGPAGPNGFAGPAGAAGQPGAKGEKGTKGPKGENGVVGPAGPVG**

**AAGPSXXXXXXXXXXXXXXXXXXGMTGFPGAAGRTGPPGPSGITGPPGPPGAAGKEGTRG**

**PRGDQGPVGRTGETGASGPPGFTGEKGPSGEPGXXGPPGSPGPQGFLGPPGILGLPGSRG**

**ERGLPGVAGALGEPGPLGIAGPPGARGPPGAVGSPGVNGAPGEAGRDGNPGSDGAPGRDG**

**QPGHKGERGYPGNIGPTGAAGAPGPHGSVGPAGKHGNRGEPXXXXXXXXXXXXXXXXXXG**

**PQGIRGDKGEPGDKGARGLPGFKGHNGLQGLPGLAXXXXXXXXXXXXXXXXXXGPAGPSG**

**PVGKDGRSGHPGPVGPAGVRGSQGSQGPXGPPGPPGPPGPPGVSGGGYDFGFEGDFYRA**

**>Heterocephalus**

**QMSYGYDEKSVG-AAVPGPMXXXXXXXXXXXXXXXGPQGFQGPPGEPGEPGASGPMGPRG**

**PPGPPGKNGDDGEAGKPGRPGERGPPGPQGARGLPGTAGLPGMKGHRXXXXXXXXXXXXX**

**XXXXXGEPGSPGENGAPGQMGPRGLPGERGRPGPPGPAXXXXXXXXXXXXXXXXXXXXXX**

**XXXXXXXXXXXGESGPQGARGSEGPQGARGEPGPPGPAGAAGPAGNPGADGQPGAKGANG**

**APGIAGAPGFPGARGPSGPQGPSGAPGPKGNSGEPGAPGNKGDTGAKGEPGPVGVQGPPG**

**PAGEEGKRGARGEPGPAGLPGPPGERGGPGSRGFPGADGVAGPKGPSGERGAPGPAGPKG**

**SPGEAGRPGEAGLPGAKGLTGSPGSPGPDGKTGPPGPAGQDGRPGPPGPPGARGQAGVMG**

**FPGPKGAAGEPGKAGERGVPGPPGAVGPAGKDGEAGAQGAPGPAGPAGERGEQGPAGSPG**

**FQGLPGPAGPPGEAGKPGEQXXXXXXXXXXXXXXXGERGFPGERGVQGPPGPAGPRGNNG**

**APGNDGAKGDAGAPGAPGSQGAPGLQGMPGERGAAGLPGPKGDRGDAGPKGADGTPGKDG**

**PRGLTGPIGPPGPAGASGDKGESGPSGPAGPTGARGAPGDRGEPGPPGPAGFAGPPGADG**

**QPGAKGEPGDAGAKGDAGPPGPAGPAGPPGPIXXXXXXXXXXXXXXXXXXGATGFPGAAG**

**RVGPPGPSGNAGPPGPPGPGGKEGAKGVRGETGPAGRPGEAGPPGPPGPAGEKGSPGADG**

**PAGAPGTPGPQGIAGQRGVVGLPGQRGERGFPGLPGPSGEPGKQGPSGSSGERGPPGPMG**

**PPGLAGPPGESGREGSPGAEGSPGRDGSPGPKGERGETGPAGPPGAPGAPGAPGPVGPAG**

**KSGDRGETGPAGPAGPIGPAGARGPAGPQGPRGDKGETGEQGDRGIKGHRGFSGLQGPPG**

**PPGSPGEQGPSGASGPAGPRGPPGSAGSPGKDGLNGLPGPIGPPGPRGRTGDAGPXGPAG**

**PPGPPGPPGPPSGGYDLSFLPQPSQEKAGDGXXXXXXRXXXX?XX?X?XXXXXGLMGPRG**

**PPGAVGAPGPQGFQGPAGEPGEPGQTXXXXXXXXXXXXXXXXXXGHPGKPGRPGERGVVG**

**PQGARGFPGTPGLPGFKGPRXXXXXXXXXXXXXXXXXXGEPGAPGENGTPGQAXXXXXXX**

**XXXXXXXXXXXGARGSDGSVGPVGPAGPIGAAGPPGFPGAPGPKGELGPVGNTGPSGPAG**

**PRGEVGLPGLSGPVGPPXXXXXXXXXXXXXXXGLPGVAGAPGLPGPRGIPGPPGAAGATG**

**ARGLVGDPGPAGSKGETGNKGEPGSAGPQGPPGPSGEEGKRGSNGEAGSAGPPGPSGLRG**

**SPGSRGLPGADGRAGVMGPPGSRGASGPAGVRGPNGDAGRPGEPGLMGPRGLPGSPGNVG**

**PAGKEGPVGLPGIDGRPGPIGPAGARGEAGNIGFPGPKGPXXXXXXXXXXXXXXXXXXXG**

**APGPDGNNGAQGPPGPQGVQGGKGEQGPAGPPGFQGLPGPSGPTGEVGKPGERGLPGEFG**

**LPGPAGPRGERGPPGESGAVGPSGPIGSRGPSGPPGPDGNKGEPGVAGAPGTAGASGPGG**

**LPGERGAVGIPGGKGEKXXXXXXXXXXXXXXXXXXGAPGAIGAPGPAGATGDRXXXXXXX**

**XXXXXXXXXXXGERGEVGPAGPNGFAGPAGAAGQPGAKGERGTKGPKGENGGVGPTGPVG**

**AAGPSGPNGPPGPAGSRGDGGPPGMTGFPGAAGRTGPPGPXGITGPPGPPGPAGKEGLRG**

**PRGDQGPAGRAGDTGAGGPPGFAGEKGPSGEPGTXGPPGTPGPQGLLGAPGILGLPGSRG**

**ERGLPGIAGTLGEPGPLGIAGPPGARGPPGNVGNPGVSGAPGEAGRDGNPGNDGPPGRDG**

**QPGHKGERGYPGNIGPTGTAGAPGPHGPVGPAGKHGNRGEPGPAGSVGPVGAVGPRGPSG**

**PQGIRGDKGEAGDKGARGLPGMKGHNGLQGLPGLAXXXXXXXXXXXXXXXXXXGPAGPSG**

**PAGKDGHSGQPGAVGPAGVRGSQGSQGPAXXXXXXXXXXXXXXXXXXXXXXXXXXXXXX**

**>Papio**

**QLSYGYDEKSTGGISVPGPMGPSGPRGLPGPPGAPGPQGFQGPPGEPGEPGASGPMGPRG**

**PPGPPGKNGDDGEAGKPGRPGERGPPGPQGARGLPGTAGLPGMKGHRGFSGLDGAKGDAG**

**PAGPKGEPGSPGENGAPGQMGPRGLPGERGRPGAPGPAGARGNDGATGAAGPPGPTGPAG**

**PPGFPGAVGAKGEAGPQGARGSEGPQGVRGEPGPPGPAGAAGPAGNPGADGQPGAKGANG**

**APGIAGAPGFPGARGPSGPQGPGGPPGPKGNSGEPGAPGSKGDTGAKGEPGPVGVQGPPG**

**PAGEEGKRGARGEPGPTGLPGPPGERGGPGSRGFPGADGVAGPKGPAGERGSPGPAGPKG**

**SPGEAGRPGEAGLPGAKGLTGSPGSPGPDGKTGPPGPAGQDGRPGPPGPPGARGQAGVMG**

**FPGPKGAAGEPGKAGERGVPGPPGAVGPAGKDGEAGAQGPPGPAGPAGERGEQGPAGSPG**

**FQGLPGPAGPPGEAGKPGEQGVPGDLGAPGPSGARGERGFPGERGVQGPPGPAGPRGANG**

**APGNDGAKGDAGAPGAPGSQGAPGLQGMPGERGAAGLPGPKGDRGDAGPKGADGSPGKDG**

**VRGLTGPIGPPGPAGAPGDKGETGPSGPAGPTGARGAPGDRGEPGPPGPAGFAGPPGADG**

**QPGAKGEPGDAGAKGDAGPPGPAGPAGPPGPIGNVGAPGPKGARGSAGPPGATGFPGAAG**

**RVGPPGPSGNAGPPGPPGPAGKEGGKGPRGETGPAGRPGEVGPPGPPGPAGEKGSPGADG**

**PAGAPGTPGPQGIAGQRGVVGLPGQRGERGFPGLPGPSGEPGKQGPSGASGERGPPGPMG**

**PPGLAGPPGESGREGAPGAEGSPGRDGSPGPKGDRGETGPAGPPGAPGAPGAPGPVGPAG**

**KSGDRGETXXXXXXXXXXXXXXXXXXXXXXXXXXXXXXXXXXXXXXXXXXXXXXXXXXXX**

**XXGSPGEQGPSGASGPAGPRGPPGSAGTPGKDGLNGLPGPIGPPGPRGRTGDAGPVXXXX**

**XXXXXXXXXXXXXXXXXXXXXXXXXXXXXXXXXXXXXRQYDG-KGVGLGPGPMGLMGPRG**

**PPGAAGAPGPQGFQGPAGEPGEPGQTGPAGSRGPAGPPGKAGEDGHPGKPGRPGERGVVG**

**PQGARGFPGTPGLPGFKGIRGHNGLDGLKGQPGAPGVKGEPGAPGENGTPGQTGARGLPG**

**ERGRVGAPGPAGARGSDGSVGPVGPAGPIGSAGPPGFPGAPGPKGELGAVGNAGPAGPAG**

**PRGEVGLPGLSGPVGPPGNPGANGLTGAKGAAGLPGVAGAPGLPGPRGIPGPVGAAGATG**

**ARGLVGEPGPAGSKGESGNKGEPGSAGPQGPPGPSGEEGKRGPNGEAGSAGPPGPPGLRG**

**GPGSRGLPGADGRAGVMGPPGSRGASGPAGVRGPNGDAGRPGEPGLMGPRGLPGSPGNIG**

**PAGKEGPVGLPGIDGRPGPIGPAGARGEPGNIGFPGPKGPTGDPGKNGDKGHAGLAGARG**

**APGPDGNNGAQGPPGPQGVQGGKGEQGPAGPPGFQGLPGPSGPAGEVGKPGERGLPGDFG**

**LPGPAGARGERGPPGESGAAGPTGPIGSRGPSGPPGPDGNKGEPGVVGAAGTAGPSGPSG**

**LPGERGAAGIPGGKGEKGEPGLRGEIGNPGRDGARGAPGAVGAPGPAGATGDRGEAGAAG**

**PAGPAGPRGSPGERGEVGPAGPNGFAGPAGAAGQPGAKGERGAKGPKGENGVVGPTGPVG**

**AAGPSGPNGPPGPAGSRGDGGPPGMTGFPGAAGRTGPPGPSGISGPPGPPGPAGKEGLRG**

**PRGDQGPVGRTGEVGAVGPPGFAGEKGPSGEAGTXGPPGTPGPQGLLGAPGILGLPGSRG**

**ERGLPGVAGALGEPGPLGIAGPPGARGPPGAVGSPGVNGAPGEAGRDGNPGNDGPPGRDG**

**QPGHKGERGYPGNNGPVGAAGAPGPHGPVGPAGKHGNRGETGPSGPVGPAGAVGPRGPSG**

**PQGIRGDKGEPGDKGPRGLPGLKGHNGLQGLPGLAGHHGDQGAPGSVGPAGPRGPAGPSG**

**PAGKDGRTGHPGTVGPAGIRGPQGHQGPAGPPGPPGPPGPPGVSGGGYDFGYDGDFYRA**

**>Microcebus**

**QMSYGYDEKSTG-ISVPGPMGPSGPRGLPGPPGAPGPQGFQGPPGEPGEPGASGPMGPRG**

**PPGPPGKNGDDGEAGKPGRPGERGPPGPQGARGLPGTAGLPGMKGHRXXXXXXXXXXXXX**

**XXXXXXXXXXXXXXXXXXXXGPRGLPGERGRPGASGPAGARGNDGATGAAGPPGPTGPAG**

**PPGFPGAVGAKGEAGPQGARGSEGPQGVRGEPGPPGPAGAAGPAGNPGADGQPGAKGANG**

**APGIAGAPGFPGARGPSGPQGPSGPPGPKGNSGEPGAPGNKGDAGAKGEPGPAGVQGPPG**

**PAGEEGKRGARXXXXXXXXXXXXXXXXXXXXXXXXXXXXXXXXXGPAGERGSPGPAGPKG**

**APGEAGRPGEAGLPGAKGLTGSPGSPGPDGKTGPPGPAGQDGRPGPPGPPGARGQAGVMG**

**FPGPKGAAGEPGKAGERGVPGPPGAVGPAGKDGEAGAQGAPGPAGPAGERGEQGPAGSPG**

**FQGLPGPAGPPGESGKPGEQGVPGDLGAPGPSGARGERGFPGERGVQGPPGPAGPRGSNG**

**APGNDGAKGDAGAPGAPGSQGAPGLQGMPGERGAAGLPGPKGDRGDAGPKGADGSPGKDG**

**VRGLTGPIGPPGPAGAPGDKGETGPSGPAGPTGARGAPGDRGEPGPPGPAGFAGPPXXXX**

**XXXXXXXXXXXXXXXXXXXXXXXXXXXXXXXXXXXXXXXXXXXXXXXXXXXXXXXXXXXX**

**XXXXXXXXXXXXXXXXXXXXXXXXXXXXXXXXXXXXXXXXXXXXXXXXXXXXXXXXXXXX**

**XXXXXXXXXXXXXXXXXXXXXXXXXXXXXXXXXXXXXXXXXXXXXXXXXXXXXXXXXXXX**

**XXXXXXXXXXXXXXXXXXXXXXXXXXXXXXXXXXXXXXXXXXXXXXXXXXXXXXXXXXXX**

**XXXXXXXXXXXXXXXXXXXXGARGPAGPQGPRGDKGETGEQGDRGIKGHRGFSGLQGPPG**

**PPGSPGEQGPSGASGPAGPRGPPGSAGAAGKDGLNGLPGPIGPPGPRGRTGDAGPVXXXX**

**XXXXXXXXXXXXXXXXXXXXXXXXXXXXXXXXXXXXXRQYDS-KGVGLGPGPMGLMGPRG**

**PPGAAGAPGPQGFQGPAGEPGEPGQTGPAGSRGPAGPPGKAGEDGHPGKPGRSGERGVVG**

**PQXXXXXXXXXXXXXXXXXXGHNGPDGLKGQPGAPGVKGEPGSPGENGTPGQTGARGLPG**

**ERGRVGAPGPAGARGSDGSVGPVGPAGPIGSAGPPGFPGAPGPKGELGPVGNPGPAGPAG**

**PRGEVGLPGLSGPVGPPGNPGANGLTGAKGAAGLPGVAGAPGLPGPRGIPGPAGAAGATG**

**ARGLVGEPGPAGSKGEGGNKGEPGSAGPQGPPGPSGEEGKRGPNGEPGSAGPAGPPGLRG**

**TPGSRGLPGADGRAGVMGPPGNRGASGPAGGRGPSGDSGRPGEPGLMGPRGLPGSPGNVG**

**PAGKEGPVGLPGIDGRPGPIGPAGARGEPGNIGFPGPKGPTGDPGKAGDRGHAGLAGARG**

**APGPDGNNGAQGPPGPQGVQGGKGEQGPAGPPGFQXXXXXXXXXXXXXXXXXXXXXXXXX**

**XXXXXXXXGERGPPGESGAAGPTGPIGSRGPSGPPGPDGNKGEPGAVGAPGTAGASGPGG**

**LPGERGAAGIPGGKGEKGESGLRGEIGNPGRDGARGAPGAVGAPGPAGATGDRGEAGAAG**

**PAGPAGPRGSPGERGEVGPAGPNGFAGPAGAAGQAGAKGERGAKGPKGENGGVGATGPAG**

**PAGPSGPNGPXGPAGGRGDGGPPGVTGFPGAAGRTGPPGPSGITGPPGPPGAAGKEGLRG**

**PRGDQGPVGRTGETGASGPPGFAGEKGPSGESGTAGPPGTPGPQGLLGAPGILGLPGSRG**

**ERGLPGVAGSVXXXXXXXXXXXXXXXXXXXXXXXXXXXXXXXEAGRDGNPGNDGPPGRDG**

**QAGHKGERGYPGNIGPVGAAGAPGPHGSVGPAGKHGNRGEPGPAGSVGPVGAVGPRGPSG**

**PQGVRGDKGEAGDKGPRGLPGLKGHAGLQGLPGLAGHHGDQGAPGSVGPAGPRGPAGPSG**

**PVGKDGRSGHPGTVGPAGIRGPQGHQGPAGPPGPPGPPGPPGAGGGGYDFGFDGDFYRA**

**>Dipodomys**

**XXXXGYDEKSAG-VSVPGPMGPSGPRGLPGPPGAPGPQGFQGPPGEPGEPGASGPMGPRG**

**PPGPPGKNGDDGEAGKPGRPGERGPSGPQGARGLPGTAGLPGMKGHRGFSGLDGAKGDAG**

**PAGPKGEPGSPGENGAPGQMGPRGLPGERGRPGAPGPAGARGNDGATGAAGPPGPTGPAG**

**PPGFPGAVGAKGEAGPQGARGSEGPQGVRGEPGPPGPAGAAGPAGNPGADGQPGAKGANG**

**APGIAGAPGFPGARGPSGPQGPSGAPGPKGNSGEPGAPGNKGDTGAKGEPGPAGVQGPPG**

**PAGEEGKRGARGEPGPAGLPGPPGERGGPGSRGFPGADGVAGPKXXXXXXGSPGPAGPKG**

**SPGEAGRPGEAGLPGAKGLTGSPGSPGPDGKTGPPGPAGQDGRPGPPGPPGARGQAGVMG**

**FPGPKGAAGEPGKAGERGVPGPPGAVGPAGKDGEAGAQGPPGPSGPAGERGEQGPAGSPG**

**FQGLPGPAGPPGEAGKPGDQGVPGDLGAPGPSGARGERGFPGERGVQGPPGPAGPRGSNG**

**APGNDGAKGDTGAPGAPGSQGAPGLQGMPGERGAAGLPGPKGDRGDAGPKGADGSPGKDG**

**VRGLTGPIGPPGPAGAPGDKGESGPSGPAGPTGARGAPGDRGEPGPPGPAGFAGPPGADG**

**QPGAKGEPGDSGAKGDAGPPGPAGPAGPPGPIGNVGAPGPKGARGSAGPPGATGFPGAAG**

**RVGPPGPSGNAGPPGPPGPVGKEGGKGPRGETGPAGRPGEVGPPGPPGPAGEKGSPGADG**

**PAGSPGTPGPQGIAGQRGVVGLPGQRGERGFPGLPGPSGEPGKQGPSGASGERGPPGPMG**

**PPGLAGPPGESGREGSPGAEGXPGRDGSPGPKGDRGETGPAGPPGAPGAPGAPGPVGPAG**

**KSGDRGETGPAGPAGPIGPVGARGPAGPQGPRGDKGETGETGERGIKGHRGFSGLQGPPG**

**PPGSPGEQGPSGASGPAGPRGPPGSAGAAGKDGLNGLPGPIGPPGPRGRTGDAGPVGPPG**

**PPGPPGPPGPPSGGFDFSFMPQPPQEKA?DGGRYYRARQYDG-KGASLGPGPMGLMGPRG**

**PPGASGAPGPQGFXGPAGEPGEPXQTGPAGARGPPGAPGKAGEDGHPGKPGRPGERGVVG**

**PQGARGFPGTPGLPGFKGIRGHNGLDGLKGQPGAPGIKGEPGAPGENGTPGQSGARGLPG**

**ERGRVGAPGPAGARGSDGSVGPVGPAGPIGSAGPPGFPGAPGPKGELGPVGSPGASGPAG**

**PRGEVGLPGLSGPVGPPGNPGANGLTGSKGAAGLPGVAGAPGLPGPRGIPGPVGAAGATG**

**PRGLVGEPGPAGSKGETGNKGEPGAAGPQGLPGPSGEEGKRGSNGEPGSAGPAGPPGLRG**

**NPGSRGLPGADGRAGVMGPPGNRGSSGPAGVRGPNGDSGRPGEPGLMGPRGLPGSPGSVG**

**PTGKEGPVGLPGIDGRPGPIGPAGARGEAGNIGFPGPKGPTGEPGKHGDKGHPGLAGARG**

**APGPDGNNGAQGPPGPQGVQGGKGEQGPAGPPGFQGLPGPSGSAGEVGKPGERGLPGEFG**

**LPGPAGPRGERGPPGESGAAGPSGPIGSRGPSGPPGPDGNKXXXXXXXXXXXXXXXXXXX**

**XXXXXXXXXXPGGKGEKGETGLRGEIGTPGRDGARXAPAAVGAPAPAGATXXXGEAGAAG**

**PAGPAGPRGSPGERGEVGPAGPNGFAGPAGAAGQPGAKGERGTKGPKGENGVVGPSGPVG**

**AAGPSGPNGPPGPVGGRGDGGPPGMTGFPGAAGRTGPPGPSXXXXXXXXXXXXXXXXXXX**

**XXXXXXXXXXXXXXXXXXXXXXXXXXXXXXXXXXXGPPGTPGPQGLLGAPGILGLPGSRG**

**ERGLPGISGALGEPGPLGIAGPPGARGPPGAVGSPGVNGAPGEAGRDGNPGSDGPPGRDG**

**QPGHKGERGYPGNIGPTGAAGAPGPQGSVGPAGKYGNRGEPGPAGSIGPVGAVGPRGPSG**

**PQGIRGEKGEVGDKGHRGLPGLKGHNGLQGLPGLAGPHGDQGSPGTVGPAGPRGPAGPTG**

**PVGKDGRSGQPGAVGPAGVRGTQGSQGPAGPPGPPGPPGPPGISGGGYDF??DGDFYRA**

**>Cavia**

**QMSYGYDEKSVG-AAVPGPMGPSGPRGLPGPPGAPGPQGFQGPPGEPGEPGASGPMGPRG**

**PPGAPGKNGDDGEPGKPGRPGERGPPGPQGARGLPGTAGLPGMKGHRGFSGLDGAKGDAG**

**PAGPKGEPGSPGENGAPGQMGPRGLPGERGRPGPPGPAGARGNDGATGAAGPPGPTGPAG**

**PPGFPGAVGAKGESGPQGARGSEGPQGARGEPGPPGPAGAAGPAXXXXXXXXXXXXXXXG**

**APGIAGAPGFPGARGPSGPQGPSGPPGPKGNSGEPGAPGSKGDTGAKGEPGPVGIQGPPG**

**PAGEEGKRGARGEPGPAGLPGPPGERGGPGSRGFPGADGVAGPKGPAGERGSPGPAGPKG**

**SPGEAGRPGEAGLPGAKGLTGSPGSPGPDGKTGPPGPAGQDGRPGPAGPPGARGQAGVMG**

**FPGPKGAAGEPGKAGERGVPGPPGAVGPAGKDGEAGAQGPPGPAGPAGERGEQGPAGSPG**

**FQGLPGPAGPPGEAGKPGEQGVPGDLGAPGPSGARGERGFPGERGVQGPPGPAGPRGSNG**

**APGNDGAKGDAGAPGAPGSQGAPGLQGMPGERGAAGLPGPKGDRGDAGPKGADGTPGKDG**

**PRGLTGPIGPPGPAGASGDKGETGPSGPPGPTGARGAPGDRGEPGPPGPAGFAGPPGADG**

**QPGAKGEPGDAGAKGDAGPPGPAGPAGPPGPIGNVGAPGSKGARGSPGPPGATGFPGAAG**

**RVGPPGPSGNAGPPGPPGPAGKEGAKGVRGETGPAGRPGEAGPPGPPGPAGEKGSPGADG**

**PAGAPGTPGPQGIAGQRGVVGLPGQRGERGFPGLPGPSGEPGKQGPSGASGERGPPGPAG**

**PPGLAGPPGESGREXXXXXXXXXXXXXXXXXXGDRGETGPAGPPGAPGAPGAPGPVGPAG**

**KNGDRGETGPAGPAGPIGPAGARGPAGPQGPRGDKGETGEQGDRGIKGHRGFSGLQGPPG**

**PPGSPGEQGPSGASGPAGPRGPPGSAGSPGKDGLNGLPGPIGPPGPRGRTGDAGPAGPPG**

**PPGPPGPPGPPSGGYDLSFLPQPPQEKSGDGGRYYRARQYDG-KGVGLGPGPMGLMGPRG**

**PPGAVGAPGPQGFQGPAGEPGEPGQTGPAGSRGPAGPPGKAGEDGHPGKPGRPGERGVVG**

**PQGARGFPGTPGLPGFKGPRGHNGMDGLKGQAGAPGVKGEPGAPGENGTPGQAGARGLPG**

**ERGRVGAPGPTGARGSDGSVGPVGPAGPIGAAGPPGFPGAPGAKGELGPVGNPGPSGPAG**

**PRGEVGLPGLSGPVGPPGNPGANGLPGSKGATGLPGVAGAPGLPGPRGIPGPVGAAGATG**

**ARGLVGDPGPAGSKGESGNKGEPGSAGAQGPPGPSGEEGKRGPNGEVGSAGPPGPPGLRG**

**SPGSRGLPGADGRSGVMGPPGSRGATGPAGVRGPNGDTGRPGEPGLMGPRGLPGSPGNAG**

**PAGKEGPMGLPGIDGRPGPIGPAGPRGEAGNIGFPGPKGPTGDPGKSGDKGHPGLAGARG**

**APGPDGNNGAQGPPGPQGVQGGKGEQGPAGPPGFQGLPGPSGPAGEVGKPGERGLPGEFG**

**LPGPAGARGERGPPGESGAVGPAGPIGNRGPSGPPGPDGNKGEPGVVGAPGTAGASGPGG**

**LPGERGAAGIPGGKGEKGETGHRGEPGNTGRDGARGAPGAIGAPGPAGATGDRXXXXXXX**

**XXXXXXXRGSPGERGEVGPAGPNGFAGPAGAAGQPGAKGERGAKGPKGENGVVGPTGPVG**

**AAGPSGPNGPPGPAGSRGDGGPPGMTGFPGAAGRTGPPGPSGITGPPGPPGPAGKEGLRG**

**PRGDQGPVGRTGDTGAGGPPGFAGEKGPSGEPGTAGPPGTPGPQGLLGAPGILGLPGSRG**

**ERGLPGIAGASGEPGPLGIAGPPGARGPPGNVGSPGVNGPPGEAGRDGNPGNDGPPGRDG**

**QPGHKGERGYPGNIGPVGAAGAPGPHGPVGPTGKHGNRGEPGPAGSVGPVGAVGPRGPSG**

**PQGIRGDKGEVGDKGPRGLPGLKGHNGLQGLPGLAGQHGDQGSPGPVGPAGPRGPAGPSG**

**PAGKDGHAGQPGPVGPAGVRGSQGSQGPAGPPGPPGPPGPAGASGGGYDFGFDGDFYRA**

**>Oryctolagus**

**QMSYGYDEKSAG-VSVPGPMGPSGPRGLPGPPGSPGPQGFQGPPGEPGEPGASGPMGPRG**

**PPGAPGKNGDDGEAGKPGRPGERGPPGPQGARGLPGTAGLPGMKGHRGFSGLDGAKGDAG**

**PAGPKGEPGSPGENGAPGQMGPRGLPGERGRPGAPGPAGARGNDGATGAAGPPGPTGPAG**

**PPGFPGAVGAKGEAGPQGARGSEGPQGVRGEPGPPGPAGAAGPAGNPGADGQPGAKGANG**

**APGIAGAPGFPGARGPSGPQGPSGPPGPKGNSGEPGAPGNKGDTGAKGEPGPTGVQGPPG**

**PAGEEGKRGARGEPGPTGLPGPPGERGGPGSRGFPGADGVAGPKGPAGERGAPGPAGPKG**

**SPGEAGRPGEAGLPGAKGLTGSPGSPGPDGKTGPPGPAGQDGRPGPPGPPGARGQAGVMG**

**FPGPKGAAGEPGKAGERGVPGPPGAVGPAGKDGEAGAQGPPGPAGPAGERGEQGPAGSPG**

**FQGLPGPAGPPGEAGKPGEQGVPGDLGAPGPSGARGERGFPGERGVQGPPGPAGPRGSNG**

**APGNDGAKGDAGAPGAPGSQGAPGLQGMPGERGAAGLPGPKGDRGDAGPKGADGSPGKDG**

**VRGLTGPIGPPGPAGAPGDKGETGPSGPAGPTGARGAPGDRGEPGPPGPAGFAGPPGADG**

**QPGAKGEPGDAGAKGDAGPAGPAGPAGPPGPIGNVGAPGPKGARGSPGPPGATGFPGAAG**

**RVGPPGPSGNAGPPGPPGPVGKEGGKGPRGETGPAGRPGEVGPPGPPGPAGEKGSPGADG**

**PAGAPGTPGPQGIAGQRGVVGLPGQRGERGFPGLPGPSGEPGKQGPSGASGERGPPGPMG**

**PPGLAGPPGESGREGSPGAEGSPGRDGAPGPKGDRGETGPAGPPGAPGAPGAPGPVGPAG**

**KSGDRGETGPAGPAGPIGPAGARGPAGPQGPRGDKGETGEQGDRGIKGHRGFSGLQGPPG**

**PPGSPGEQGPSGASGPAGPRGPPGSAGAPGKDGLNGLPGPIGPSGPRGRTGDAGPVXXXX**

**XXXXXXXXXXXXXXXXXXXXXXXXXXXXXXXXXXXXXRQFDG-KG-G-GPGPMGLMGPRG**

**PPGAAGAPGPQGFQGPAGEPGEPGQTGPAGARGPPGPPGKAGEDGHPGKPGRPGERGVMG**

**PQGARGFPGTPGLPGFKGIRGHNGLDGLKGQPGAPGVKGEPGAPGENGTPGQTGARGLPG**

**ERGRVGAPGPAGARGSDGSVGPVGPAGPIGSAGPPGFPGAPGPKGELGPVGNPGPSGPAG**

**PRGEVGLPGVSGPVGPPGNPGANGLTGAKGAAGLPGVAGAPGLPGPRGIPGPVGAAGATG**

**ARGLVGEPGPAGTKGESGNKGEPGSAGPQGPPGPSGEEGKRGSPGEPGSAGPAGPPGLRG**

**SPGSRGLPGADGRAGVMGPPGSRGSTGPAGVRGPNGDSGRPGEPGLMGPRGLPGSPGNVG**

**PAGKEGPVGLPGIDGRPGPIGPAGARGEPGNIGFPGPKGPTGDPGKNGDKGHPGLAGARG**

**APGPDGNNGAQGPPGPQGVQGGKGEQGPAGPPGFQGLPGPSGTAGEVGKPGERGLPGEFG**

**LPGPAGPRGERGAPGESGAAGPPGPIGSRGPSGPPGPDGNKGEPGVVGAPGTAGASGPGG**

**LPGERGAAGIPGGKGEKGETGLRGEIGNPGRDGARGAPGAVGAPGPAGATGDRGEAGAAG**

**PAGPAGPRGSPGERGEVGPAGPNGFAGPAGAAGQPGAKGEKGTKGPKGENGVVGPAGPVG**

**AAGPSGPNGPPGPAGGRGDGGPPGMTGFPGAAGRTGPPGPSGITGPPGPPGAAGKEGLRG**

**PRGDQGPVGRTGETGASGPPGFPGEKGPSGEAGTAGPPGTPGPQGLLGAPGILGLPGSRG**

**ERGLPGVAGALGEPGPLGIAGPPGARGPPGAVGSPGVNGAPGEAGRDGNPGSDGPPGRDG**

**QPGHKGERGYPGNAGPVGAAGAPGPQGSVGPTGKHGNRGEPGPAGSIGPVGAAGPRGPSG**

**PQGIRGDKGEPGDKGPRGLPGLKGHNGLQGLPGLAGQHGDQGAPGAVGPAGPRGPAGPTG**

**PAGKDGRSGHPGTVGPAGIRGSQGSQGPAGPPGPPGPPGPPGASGGGYDFGYDGDFYRA**

**>Ictidomys**

**QMSYGYDEKSAG-VSVPGPMGPSGPRGLPGPPGAPGPQGFQGPPGEPGEPGASGPMGPRG**

**PPGAPGKNGDDGEAGKPGRPGDRGPPGPQGARGLPGTAGLPGMKGHRGFSGLDGAKGDAG**

**PAGPKGEPGSPGENGAPGQMGPRGLPGERGRPGAPGPAGARGNDGATGAAGPPGPTGPAG**

**PPGFPGAVGAKGEAGPQGARGSEGPQGVRGEPGPPGPAGAAGPAGNPGADGQPGAKGANG**

**APGIAGAPGFPGARGPSGPQGPSGPPGPKGNSGEPGAPGNKGDPGAKGEPGPTGVQGPPG**

**PAGEEGKRGARGEPGPAGLPGPPGERGGPGSRGFPGADGVAGPKGPAGERGSPGPAGPKG**

**SPGEAGRPGEAGLPGAKGLTGSPGSPGPDGKTGPPGPAGQDGRPGPPGPPGARGQAGVMG**

**FPGPKGAAGEPGKTGERGVPGPPGAVGPAGKDGEAGAQGPPGPAGPAGERGEQGPAGSPG**

**FQGLPGPAGPPGEAGKPGEQGVPGDLGAPGPSGARGERGFPGERGVQGPPGPAGPRGSNG**

**APGNDGAKGDAGAPGAPGSQGAPGLQGMPGERGAAGLPGPKGDRGDAGPKGADGSPGKDG**

**VRGLTGPIGPPGPAGAPGDKGETGPSGPAGPTGARGAPGDRGEAGPPGPAGFAGPPGADG**

**QPGAKGEPGDAGAKGDAGPPGPAGPAGPPGPIGNVGAPGPKGARGSAGPPGATGFPGAAG**

**RVGPPGPSGNAGPPGPPGPAGKEGGKGPRGETGPAGRVGEVGPPGPPGPAGEKGSPGADG**

**PAGAPGTPGPQGIAGQRGVVGLPGQRGERGFPGLPGPSGEPGKQGPSGASGERGPPGPMG**

**PPGLAGPPGESGREGSPGAEGSPGRDGSPGPKGDRGETGPAGPPGAPGAPGAPGPVGPAG**

**KSGDRGETGPAGPAGPIGPAGARGPAGPQGPRGDKGETGEQGDRGIKGHRGFSGLQGPPG**

**PPGSPGEQGPSGASGPAGPRGPPGSAGSPGKDGLNGLPGPIGPPGPRGRTGDAGPVGPPG**

**PPGPPGPPGPPSGGFDFSFMPQPPQEKAGDG-RYYRARQYDG-KGVGMGPGPMGLMGPRG**

**PPGAAGAPGPQGFQGPAGEPGEPGQTGPAGARGPPGAPGKAGEDGHPGKPGRPGERGVVG**

**PQGARGFPGTPGLPGFKGIRGHNGLDGLKGQPGAQGVKGEPGAPGENGTPGQAGARGLPG**

**ERGRVGAPGPAGARGSDGSVGPVGPAGPIGSAGPPGFPGAPGPKGELGPVGNPGPSGPAG**

**PRGEVGLPGLSGPVGPPGNPGANGLTGAKGAAGLPGVAGAPGLPGPRGIPGPVGAAGATG**

**ARGLVGEPGPAGSKGESGNKGEPGSAGPQGPPGPSGEEGKRGPNGEPGSAGPAGPPGLRG**

**NPGSRGLPGADGRAGVMGPPGNRGATGPAGVRGPNGDSGRPGEPGLMGPRGLPGSPGNVG**

**PAGKEGPVGLPGIDGRPGPIGPAGARGEAGNIGFPGPKGPTGDPGKSGDKGHPGLAGARG**

**APGPDGNNGAQGPPGPQGVQGGKGEQGPAGPPGFQGLPGPSGTAGEVGKPGERGLPGEFG**

**LPGPAGPRGERGPPGESGAVGPAGPIGSRGPSGPPGPDGNKGEPGVVGAPGTAGASGPGG**

**LPGERGAAGIPGGKGEKGEPGLRGEIGNPGRDGARGAPGAVGAPGPAGATGDRGEAGAAG**

**PAGPPGPRGSPGERGEVGPAGPNGFAGPAGAAGQPGAKGERGTKGPKGENGVVGPAGPVG**

**AAGPSGPNGPPGPAGGRGDGGPPGMTGFPGAAGRTGPPGPSGITGPPGPPGAAGKEGLRG**

**PRGDQGPVGRTGETGASGPPGFAGEKGPAGEPGTAGPPGTPGPQGLLGAPGILGLPGSRG**

**ERGLPGIAGALGEPGPLGIAGPPGARGPPGAVGSPGVNGAPGEAGRDGNPGSDGPPGRDG**

**QPGHKGERGYPGNIGPAGAAGAPGPHGTVGPAGKHGNRGEPGPAGSVGPVGAVGPRGPSG**

**PQGVRGDKGEPGDKGPRGLPGLKGHNGLQGLPGLAGQHGDQGSPGPVGPAGPRGPAGPSG**

**PVGKDGRSGHPGSVGPAGVRGSQGSQGPAGPPGPPGPPGPPGVSGGGYDFGYEGDFYRA**

**>Monodelphis**

**QMSYGYDEKSGGGMSVPGPMGPSGPRGLPGPPGNPGPQGFQGPPGEPGEPGASGPMGPRG**

**PAGPPGKNGDDGEAGKPGRPGERGPPGPQGARGLPGTAGLPGMKGHRGFSGLDGAKGDSG**

**PAGPKGEPGSPGENGAPGQMGPRGLPGERGRPGPPGPAGARGNDGATGAAGPPGPTGPAG**

**PPGFPGAVGAKGEAGPQGSRGSEGPQGVRGEPGPPGPAGAAGPSGNPGADGQPGAKGANG**

**APGIAGAPGFPGARGPSGPQGPSGAPGPKGNSGEPGAPGNKGDPGAKGEPGPVGVQGPPG**

**PAGEEGKRGSRGEPGPSGLPGPAGERGGPGSRGFPGADGVAGPKGAPGERGAPGPAGPKG**

**SPGEAGRPGEAGLPGAKGLTGSPGSPGPDGKTGPPGPAGQDGRPGPPGPPGARGQAGVMG**

**FPGPKGAAGEPGKAGERGVPGPPGAVGAAGKDGEAGAQGPPGPAGPAGERGEQGPAGSPG**

**FQGLPGPAGPPGEAGKPGEQGVPGDAGAPGPSGARGERGFPGERGVQGPPGPQGPRGSNG**

**APGNDGAKGDAGAPGAPGGQGPPGLQGMPGERGAAGLPGAKGDRGDAGPKGADGAAGKDG**

**VRGLTGPIGPPGPAGPTGDKGESGPSGPVGPTGARGAPGERGEPGPPGPAGFAGPPGADG**

**QPGAKGEPGDAGAKGDAGPPGPAGPTGAPGPAGNVGAPGPKGARGNAGPPGATGFPGAAG**

**RVGPPGPSGNAGPPGPPGPAGKEGGKGPRGETGPIGRPGEVGPPGPPGPSGEKGSPGADG**

**PAGAPGTPGPQGIAGQRGVVGLPGQRGERGFPGLPGPSGEPGKQGPSGISGERGPPGPAG**

**PPGLAGPPGESGREGSPGAEGSPGRDGSPGPKGDRGETGPAGPPGAPGAPGAPGPVGPAG**

**KSGDRGETGPAGPAGPVGPTGARGPSGPQGPRGDKGETGEQGDRGMKGHRGFSGLQGPPG**

**PPGSPGEQGPSGASGPAGPRGPPGSAGASGKDGLNGLPGPIGPPGPRGRTGDAGPAGPPG**

**PPGPAGPPGPPSGGFDFSFLPQPPQEKAHDSGRYYRARXXXX?XX?X?XXXXXXXXXXXX**

**XXXXXXXXXXXXXXXXXXXXXXXXXXXXXXXXXXXXXXXXXXXXXXXXXXXXXXXXXXXX**

**XXXXXXXXXXXXXXXXXXXXXXXXXXXXXXXXXXXXXXXXXXXXXXXXXXXXXXXXXXXX**

**XXXXXXXXXXXXXXXXXXXXXXXXXXXPIGSAGPPGFPGAPGPKGELGPVGNPGPAGPAG**

**PRGELGLPGMTGPVGPAGNPGANGLTGAKGAAGLPGVAGAPGLPGPRGIPGPAGAAGASG**

**PRGLAGEPGPAGSKGESGNKGEPGSAGPQGPPGPNGEEGKRGPNGEPGSSGPAGPPGLRG**

**VPGSRGLPGADGRAGGMGPPGNRGSSGPAGVRGPNGDAGRPGEPGLMGPRGLPGSPGNSG**

**PTGKEGPAGLPGADGRPGPTGPAGNRGEPGNIGFPGPKGPTGDPGKSGEKGHAGLAGARG**

**APGPDGNNGAQGPPGPAGVQGGKGEQGPAGPPGFQGLPGPSGPAGEGGKVGERGLAGEFG**

**LPGPAGPRGERGPPGESGAVGPTGSIGSRGPSGPPGPDGNKGEPGVVGAPGNAGPAGSGG**

**VPGERGAAGVPGGKGDKGETGPRGEFGNPGRDGARGAPXXXXXXXXXXXXXXXXXXXXXX**

**XXXXXXXXXXXXXXXXXXXXXXXXXXXXXXXXXXXXXXXXXXXXXXXXXXXXXXXXXXXX**

**XXXXXXXXXXXXXXXXXXXXXXXXXXXXXXXXXXXXXXXXXXXXXXXXXXXXXXXXXXXX**

**XXXXXXXXXXXXXXXXXXXXXXXXXXXXXXXXXXXGPPGSSGPQGLLGAPGILGLPGSRG**

**ERGLPGVSGSLGEPGPLGIAGPPGARGPPGAVGSPGVNGAPGEAGRDGNPGNDGPPGRDG**

**LSGHKGERGYPGNPGAVGNAGAPGPHGTVGPAGKAGNRGEPXXXXXXXXXXXXXXXXXXX**

**XXXXXXXXXXXXXXXXXXXXXXXXXXXXXXXXXXXXXXXXXXXXXXXXXXXXXXXXXXXX**

**XXXXXXXXXXXXXXXXXXXXXXXXXXXXXXXXXXXXXXXXXXXXXXXXXXXXXXXXXXX**

**>Trichechus**

**XXXXXXXXXXXX?XXXXXPXGPSGPRGLPGPPGAPGPQGFQGPPGEPGEPGASXXXXXXX**

**XXXXXXXXXXXGEAGKPGRPGERGPPGPQGARGLPGTAGLPGMKGHRGFSGLDGAKGDAG**

**PAGPKGEPGSPGENGAPGQMGPRGLPGERGRPGXXXXXGARGNDGATGAAGPPGPTGPAG**

**PPGFPGAVGAKGEAGPQGSRGSEGPQGVRGEPGPPGPAGAAGPAGNPGADGQPGAKGANG**

**APGIAGAPGFPGARGPSGPQGPSGAPGPKGNSGEPGAPGSKGDAGAKGEPGPTGIQGPPG**

**PAGEEGKRGARGEPGPTGLPGPPGERGGPGSRGFPGADGVAGPKGPAGERGSPGPAGPKG**

**SPGEAGRPGEAGLPGAKGLTGSPGSPGPDGKTGPPGPAGQDGRPGPPGSPGARGQAGVMG**

**FPGPKGAAGEPGKAGERGVPGPAGAVXXXXXXXXXXXXXXXXXXGPAGERGEQGPAGSPG**

**FQGLPGPAGPPGEAGKPGEQGVPGDLGAPGPSGARGERGFPGERGVQGPPGPAGPRGSNG**

**APGNDGAKGDAGAPGAPGSQGAPGLQGMPGERGAAGLPGPKGDRGDAGPKGADGSPGKDG**

**ARGLTGPIGPPGPAGAPGDKXXXXXXXXXXXXXXXXXXGDRGEPGPPGPAGFAGPPGADG**

**QPGAKGEPGDAGAKGDAGPPGPAGPTGAPGPIGNVGAPGTKGARGSAGPPGATGFPGAAG**

**RVGPPGPSGNAGPPGPPGPAGKEGSKGPRGETGPAGRPGEVGPPGPPGVTGEKGSPGADG**

**PAGAPGTPGPQGIGGQRGVVGLPGQRGERGFPGLPGPSGEPGKQGPSGPGGERGPPGPVG**

**PPGLAGPPGESGREGSPGAEGSPGRDGSPGPKGDRGESGPAGPPGAPGAPGAPGPVGPAG**

**KSGDRGETGPAGPAGPVGPAGVRGPAGPQGPRGDKGETGEQGDRGIKGHRGFSGLQGPPG**

**PPGSPGEQGPSGASGPAGPRGPPGSAGSPGKDGLSGLPGPIGPPGPRGRTGDAGPVGPPG**

**PPGPPGPPGPPSAGFDFSFLPQPPQEKAHDGGRYYRARXXXX?XX?X?XXXXXGLMGPRG**

**PPGATGPPXXXXXXXXXXXXXXXXXXGPAGSRGPPGPPGKAGEDGHPGKPGRPGERGVVG**

**PQGARGFPGTPGLPGFKGIRGHNGLDGLKGQPGAPGVKXXXXXXXXXXXXXXXGARGLPG**

**ERGRVGGPGPAGARGSDGSVGPVGPAXXXXXXXXXXXXXXXXXXGELGPVGNPGPAGPAG**

**PRGEVGLSGVSGPVGPPGNPGANGLAGAKGAAGLPGVAGAPGLPGPRGIPGPPGSAGATG**

**ARGLVXXXXXXXXXXXXXXXXXXGSAGPQGPPGPSGEEGKRGPNGEAGSTGPAGPPGLRV**

**GXXXXXXXXXXXXXXXXGLPGSRGATGPAGVRGPSGDAGRPGEPGVMGPRGLPGSPGNVG**

**PAGKEGPAGLPGIDGRLGPAGPTGARGEPGNIGFPGPKGPXGDPGKNGEKGHAGLAGPRG**

**APGPDGNNGAQGPPGPQGVQGGKGEQGPAGPPGFQGLPGPAGTAGEAGKPGERXXXXXXX**

**XXXXXXXXGERGPPGPSGATGPAGPTGSRGPSGPPGPDGNKGEPGVVGAPGTAGPSGPSG**

**LPGERGASGIPGGKGEKXXXXXXXXXXXXXXXXXXGPPGAVGAPGPAGATGDRXXXXXXX**

**XXXXXXXXXXXGERGEVGPAGPNGFAGPAGAAGQPGAKGERGTKGPKGENGPVGPTGPVG**

**AVGPAGPNGPPGPAGSRGDGGPPGATGFPGAAGRTGPPGPXGITGPPGPPGPAGKEGLRG**

**PRGDQGPVGRTGETGASGPLGFTGEKGPPGEPGAAGPPGTPGPQGLLGPPGILGLPGTRG**

**ERGLPGVAGAVGEPGPLGIAGPAGARGPPGAVGSPGVNGAPGEAGRDGNPGSDGPPGRDG**

**LPGHKGDRGYPGNAGPVGTAGAPGPHGSVGPAGKQGSRGEPGPAGSVGPVGAVGPRGPXG**

**PQGIRGDKGEPGEKGPRGLPGLKGHNGLQGLPGLAGQHGDQGSPGTVGPAGPRGPAGPSG**

**PVGKDGRPGHSGPVGPAGVRGSQGSQGPXXXXXXXXXXXXXXXXXXXXXXXXXXXXXXX**

**>Loxodonta**

**QLSYGYDEKSAGGISVPGPMGPSGPRGLPGPPGAPGPQGFQGPPGEPGEPGASGPMGPRG**

**PPGPPGKNGDDGEAGKPGRPGERGPPGPQGARGLPGTAGLPGMKGHRGFSGLDGAKGDAG**

**PAGPKGEPGSPGENGAPGQMGPRGLPGERGRPGAPGPAGARGNDGATGAAGPPGPTGPAG**

**PPGFPGAVGAKGEAGPQGARGSEGPQGVRGEPGPPGPAGAAGPAGNPGADGQPGAKGANG**

**APGIAGAPGFPGARGPAGPQGPSGAPGPKGNSGEPGAPGSKGDAGAKGEPGPVGIQGPPG**

**PAGEEGKRGARGEPGPTGLPGPPGERGGPGSRGFPGADGVAGPKGPAGERGSPGPAGPKG**

**SPGEAGRPGEAGLPGAKGLTGSPGSPGPDGKTGPPGPAGQDGRPGPPGPPGARGQAGVMG**

**FPGPKGAAGEPGKAGERGVPGPPGAVGAAGKDGEAGAQGPPGPAGPAGERGEQGPAGSPG**

**FQGLPGPAGPPGEAGKPGEQGVPGDLGAPGPSGARGERGFPGERGVQGPPGPAGPRGSNG**

**APGNDGAKGDAGAPGAPGSQGAPGLQGMPGERGAAGLPGPKGDRGDAGPKGADGSPGKDG**

**PRGLTGPIGPPGPAGAPGDKGEAGPSGPAGPTGARGAPGDRGEPGPPGPAGFAGPPGADG**

**QPGAKGEPGDAGAKGDAGPPGPAGPTGAPGPIGNVGAPGPKGARGSAGPPGATGFPGAAG**

**RVGPPGPSGNAGPPGPPGPAGKEGGKGPRGETGPAGRPGEVGPPGPPGPAGEKGSPGADG**

**PAGAPGTPGPQGIGGQRGVVGLPGQRGERGFPGLPGPSGEPGKQGPSGSSGERGPPGPAG**

**PPGLAGPPGESGREGAPGAEGSPGRDGSPGPKGDRGETGPSGPPGAPGAPGAPGPVGPAG**

**KSGDRGETGPAGPAGPAGPAGVRGPAGPQGPRGDKGETGEQGDRGIKGHRGFSGLQGPPG**

**PPGSPGEQGPSGASGPAGPRGPPGSAGAPGKDGLNGLPGPIGPPGPRGRTGDAGPVGPPG**

**PPGPPGPPGPPSGAFDFSFLPQPPQEKAHDGGRYYRARQYDA-KGIGLGPGPMGLMGPRG**

**PPGATGPPGSPGFQGPPGEPGEPGQTGPAGSRGPAGPPGKAGEDGHPGKPGRPGERGVVG**

**PQGARGFPGTPGLPGFKGIRGHNGLDGLKGQPGAPGVKGEPGAPGENGTPGQIGARGLPG**

**ERGRVGGPGPAGARGSDGSVGPVGPAGPIGSAGPPGFPGAPGPKGELGPVGNPGPSGPAG**

**PRGEAGLPGVSGPVGPPGNPGANGLAGAKGAAGLPGVAGAPGLPGPRGIPGPVGAAGATG**

**ARGLVGEPGPAGSKGESGSKGEPGSAGPQGPPGPSGEEGKRGSSGEAGSAGPAGPPGLRG**

**GPGSRGLPGADGRAGVMGPPGSRGASGPAGVRGPSGDSGRPGEPGVMGPRGLPGSPGNVG**

**PAGKEGPAGLPGIDGRPGPIGPAGARGEPGNIGFPGPKGPAGDPGKNGDKGHAGLAGPRG**

**APGPDGNNGAQGPPGLQGVQGGKGEQGPAGPPGFQGLPGPSGTAGEAGKPGERGLPGEFG**

**LPGPAGPRGERGPPGQSGAAGPTGPIGSRGPSGPPGPDGNKGEPGVVGAPGTAGPSGPGG**

**LPGERGAAGIPGGKGEKGETGLRGDTGNTGRDGARGAPGAVGAPGPAGATGDRGEAGPAG**

**SAGPAGPRGSPGERGEVGPAGPNGFAGPAGAAGQAGAKGERGTKGPKGENGPVGPTGPVG**

**AAGPAGPNGPPGPAGSRGDGGPPGATGFPGAAGRTGPPGPAGITGPPGPPGAAGKEGLRG**

**PRGDQGPVGRTGETGASGPPGFAGEKGSSGEPGTAGPPGTPGPQGLLGPPGILGLPGSRG**

**ERGLPGVAGAVGEPGPLGIAGPPGARGPPGAVGSPGVNGAPGEAGRDGNPGSDGPPGRDG**

**LPGHKGERGYPGNAGPVGTAGAPGPQGPLGPAGKHGNRGEPGPAGSVGPVGAVGPRGPSG**

**PQGARGDKGEAGDKGPRGLPGFKGHNGLQGLPGLAGQHGDQGSPGSVGPAGPRGPAGPSG**

**PVGKDGRPGHAGAVGPAGVRGSQGSQGPSGPPGPPGPPGPPGPSGGGYDFGYDGDFYRA**

**>Callithrix**

**QLSYGYDEKSTGGISVPGPMGPSGPRGLPGPPGSPGPQGFQGPPGEPGEPGASGPMGPRG**

**PPGPPGKNGDDGEAGKPGRPGERGPPGPQGARGLPGTAGLPGMKGHRGFSGLDGAKGDAG**

**PAGPKGEPGSPGENGAPGQMGPRGLPGERGRPGPPGPAGARGNDGATGAAGPPGPTGPAG**

**PAGFPGAVGAKGEAGPQGPRGSEGPQGVRGEPGPPGPAGAAGPAGNPGADGQPGAKGANG**

**APGIAGAPGFPGARGPSGPQGPSGPPGPKGNSGEPGAPGSKGDTGAKGEPGPVGVQGPPG**

**PAGEEGKRGARGEPGPTGLPGPPGERGGPGSRGFPGADGVAGPKGPAGERGSPGPAGPKG**

**SPGEAGRPGEAGLPGAKGLTGSPGSPGPDGKTGPPGPAGQDGRPGPPGPPGARGQAGVMG**

**FPGPKGAAGEPGKAGERGVPGPPGAVGPAGKDGEAGAQGPPGPAGPAGERGEQGPAGSPG**

**FQGLPGPAGPPGEAGKPGEQGVPGDLGAPGPSGARGERGFPGERGVQGPPGPAGPRGANG**

**APGNDGAKGDAGAPGAPGSQGAPGLQGMPGERGAAGLPGPKGDRGDAGPKGADGSPGKDG**

**VRGLTGPIGPPGPAGAPGDKGETGPSGPAGPTGARGAPGDRGEPGPPGPAGFAGPPGADG**

**QPGAKGEPGDAGAKGDAGPPGPAGPAGPPGPIGNVGAPGPKGARGSAGPPGATGFPGAAG**

**RVGPPGPSGNAGPPGPPGPAGKEGGKGPRGETGPAGRPGEVGPPGPPGPAGEKGSPGADG**

**PAGAPGTPGPQGIAGQRGVVGLPGQRGERGFPGLPGPSGEPGKQGPSGTSGERGPPGPMG**

**PPGLAGPPGESGREGAPGAEGSPGRDGSPGPKGDRGETGPAGPPGAPGAPGAPGPVGPAG**

**KSGDRGETGPAGPAGPIGPVGSRGPAGPQGPRGDKGETGEQGDRGIKGHRGFSGLQGPPG**

**PPGSPGEQGPSGASGPAGPRGPPGSAGAPGKDGLNGLPGPIGPPGPRGRTGDAGPVGPPG**

**PPGPPGPPGPPSGGLDFSFLPQPXXXXAHDGGRYYRARQYDG-KGVGLGPGPMGLMGPRG**

**PPGAAGAPGPQGFQGPAGEPGEPGQTGPAGARGPPGPPGKAGEDGHPGKPGRPGERGVVG**

**PQGARGFPGTPGLPGFKGIRGHNGLDGLKGQPGAPGVKGEPGAPGENGTPGQTGARGLPG**

**ERGRVGAPGPAGARGSDGSVGPVGPAGPIGSAGPPGFPGAPGPKGELGAIGNPGIAGPAG**

**PRGEVGLPGLSGPVGPPGNPGANGLTGAKGAAGLPGVAGAPGLPGPRGIPGPVGAAGATG**

**ARGLVGEPGPAGSKGESGNKGEPGSAGPQGPPGPSGEEGKRGPNGEAGSAGPPGPPGLRG**

**SPGSRGLPGADGRAGVMGPAGSRGATGPAGVRGPNGDAGRPGEPGLMGPRGLPGSPGNIG**

**PAGKEGPVGLPGIDGRPGPIGPAGARGEPGSIGFPGPKGPTGDPGKNGDKGHAGLAGARG**

**APGPDGNNGAQGPPGPQGVQGGKGEQGPAGPPGFQGLPGPSGPAGELGKPGERGLPGEFG**

**LPGPAGPRGERGPPGESGAAGPTGPIGSRGPSGPPGPDGNKGEPGVVGAAGTAGPSGPSG**

**LPGERGAAGIPGGKGEKGEPGLRGEIGNPGRDGARGAPGAVGAPGPAGATGDRGEAGAAG**

**PAGPAGPRGSPGERGEVGPAGPNGFAGPAGAAGQPGAKGERGAKGPKGENGVVGPTGPVG**

**AAGPXXXXXPPGPAGSRGDGGPPGMTGFPGAAGRTGPPGPSGISGPPGPPGPAGKEGLRG**

**PRGDQGPVGRTGETGAVGPPGFAGEKGPSGEAGTAGPPGTPGPQGLLGAPGILGLPGSRG**

**ERGLPGVAGAVGEPGPLGIAGPPGARGPPGAVGSPGVNGAPGEAGRDGNPGNDGPPGRDG**

**QPGHKGERGYPGNIGPVGAAGAPGPHGPVGPAGKHGNRGETGPSGPVGPAGAVGPRGPSG**

**PQGIRGDKGEPGDKGPRGLPGLKGHNGLQGLPGLAGHHGDQGAPGSVGPAGPRGPAGPSG**

**PAGKDGRTGHPGTVGPAGIRGPQGHQGPAGPPGPPGPPGPPGVSGGGYDFGYDGDFFRA**

**>Macropus**

**QMSYGYDEKSGG-ISVPGPMXXXXXXXXXXXXXXXXXXXXXXXXXXXXXXXXXXXXXXXX**

**XXXXXXXXXXXGEAGKPGRPGERGPPGPQGARGLPGTAGLPGMKGHRGFSGLDGAKGDSG**

**PAGPKGEPGSPGENGAPGQMGPRGLPGERGRPGPPGPAGARGNDGATGAAGPPGPTGPAG**

**PPGFPGAVGAKGEAGPQGARGSEGPQGVRGEPGPPGPAGAAGPSGNPGADGQPGAKGANG**

**APGIAGAPGFPGARGPSGPQGPSGAPGPKGNSGEPGAPGNKGDAGAKGEPGPVGVQGPPG**

**PAGEEGKRGSRGEPGPTGLPGPAGERGGPGSRGFPGADGVAGPKGAPGERGAPGPAGPKG**

**SPGESGRPGEAGLPGAKGLTGSPGSPGPDGKTGPPGPAGQDGRPGPPGPPGARGQAGVMG**

**FPGPKGAAGEPGKAGERGVPGPPGAVGAAGKDGEAGAQGPPGPAGPAGERGEQGPAGSPG**

**FQGLPGPAGPPGEAGKPGEQGVPGDAGAPGPSGARGERGFPGERGVQGPPGPQGPRGANG**

**APGNDGAKGDAGAPGAPGSQGPPGLQGMPGERGAAGLPGAKGDRGDAGPKGADGAPGKDG**

**VRGLTGPIGPPGPAGPSGDKGESGPSGPVGPTGARGAPGERGEPGPPGPAGFAGPPGADG**

**QPGAKGEPGDAGAKGDAGPPGPAGPTGAPGPAGNVGAPGPKGARGSAGPPGATGFPGAAG**

**RVGPPGPSGNTGPPGPPGPAGKEGGKGPRGETGPVGRPGEVGPPGPPGPSGEKGSPGADG**

**PAPVPXXXXXXXXXXXXXXXXXXXXXXXXXXXXXXXXXXXXXXXXXSGVSGERGPPGPAG**

**PPGLAGPPGESGREGAPGAEGSPGRDGAPGAKGDRGETGPAGPPGAPGAPGAPGPVGPAG**

**KAGDRGETXXXXXXXXXXXXXXXXXXGPQGPRGDKGETGEQGDRGIKGHRGFSGLQGPPG**

**PPGSPGEQGPSGASGPAGPRXXXXXXXXXXXXXXXXXXXXXXXXXXXXXXXXXXXXGPPG**

**PPGPPGPPGPPSGGFDFSFLPQPPQEKAHDSGRYYRARQYDASKGIDMGPGPMGLMGPRG**

**PRGASGPPGAQGFQGPAGEPGEPGQTGPAGARGPPGPPGKSGEDGHPGKPGRPGERGIVG**

**PQXXXXXXXXXXXXXXXXXXXXXXXXXXXXXXXXXXXXGEPGAPGENGTPGQAGARGLPG**

**ERGRIGGAGPAGARGSDGSVGPVGPAGPIGSAGPPGFPGAPGPKGELGPVGNPGPAGPAG**

**PRGELGLPGMTGPVGPAGNPGANGLTGAKGAAGLPGVAGAPGLPGPRGIPGPAGAAGASG**

**PRGLAGEPGPAGAKGESGNKGEPGAAGPQGPPGPSGEEGKRGPNGEPGSTGPTGPPGLRG**

**VPGSRGLPGADGRAGGMGPPGNRGSSGPAGARGPNGDAGRPGEPGLMGPRGLPGSPGNPG**

**PTGKEGPAGLPGPDGRPGPTGPAGNRGEPGNIGFPGPKGPNGEPGKSGEKGHAGLAGARG**

**APGPDGNNGAQGPPGPAGVQGGKGEQGPAGPPGFQGLPGPSGPAGEGGKVGERGLPGEFG**

**LPGPAGPRXXXXXXXXXXXXXXXXXXXXXXXXXXXXXXXXXGEPGVVGAPGSAGPAGSGG**

**VPGERGAAGVPGGKGEKGETGLRGDFGNPGRDGARGAPGAMGAPGPAGATGERGEAGPAG**

**PVGPTGARGAPGDRGEAGPAGPNGFAGPPGAAGQAGAKGERGTKGPKGENGVVGPTGPVG**

**AAGPAGPNGPPGPVGGRGDGGPPGATGFPGAAGRTGAPGPAGITGPPGPPGASGKEGPRG**

**PRGDQGPLGRAGETGAVGPPGFAGEKGPPGEAGATGPPGSSGPQGLLGAPGILGLPGSRG**

**ERGLPGVSGALGEPGPLGIAGPPGARGPPGAVGNPGVNGAPGEAGRDGNPGNDGPPGRDG**

**LAGHKGERGYPGNAGAVGNAGAPGPHGTVGPAGKPGNRGEPGPVGSVGPAGPFGARGPSG**

**PQGPRGDKGEVGDKGPRGLNGLKGHNGFQGLPGLAGQHGDQGAPGSIGPAGPRGPAGPSG**

**PAGKDGRPGQAGAVGPAGIRGSQGSQGPAGPPGPPGLPGPPGPSGGGYDFGYDGDFYRA**

**>Procavia**

**QLSYGYDEKSAGGISVPGPMGPSGPRGLPGPPGAPGPQGFQGPPGEPGEPGASGPMGPRG**

**PPGPPGKNGDDGEAGKPGRPGERGPPGPQGARGLPGTAGLPGMKGHRGFSGLDGAKGDAG**

**PAGPKGEPGSPGENGAPGQMGPRGLPGERGRPGPPGPAGARGNDGAAGAAGPPGPTGPAG**

**PPGFPGAVGAKGEGGPQGPRGSEGPQGVRGEPGPPGPAGAAGPAGNPGADGQPGAKGANG**

**APGIAGAPGFPGARGPSGPQGPSGAPGPKGNSGEPGAPGSKGDAGAKGEPXXXXXXXXXX**

**XXXXXXXXXXXXXXXXXXXXXXXXXXXXXXXXXXXXXXXXXXXXXXXXXXXXXXXXXXXX**

**XXXXXXXXXXXXXXXXXXXXXXXXXXXXXXXXXXXXXXXXXXXXXXXXXXXXXXXXXXXX**

**XXXXXXXXGEPGKAGERGVPGPAGAVGAPGKDGEAGAQGPPGPAGPAGERGEQGPAGSPG**

**FQGLPGPAGPPGEAGKPGEQGVPGDLGAPGPSGARGERGFPGERGVQGPPGPAGPRGSNG**

**APGNDGAKGDAGAPGAPGSQGAPGLQGMPGERGAAGLPGPKGDRGDAGPKGADGXXXXXX**

**XXXXXXXXXXXXXXXXXXXXXXXXXXXXXXXXXXXXXXXXXXXXXXXXXXXXXXXXXXXX**

**XXXXXXXXXXXXXXXXXXXXXXXXXXXXXXXXGNVGAPGPKGARGSAGPPGATGFPGAAG**

**RVGPPGPSGNAGPPGPPGPAGKEGGKGPRGETGPAGRPGEVGPPGPPGPAGEKGSPGADG**

**PAGAPGTPGPQGIGGQRGVVGLPGQRGERGFPGLPGPSGEPGKQGPSGPNGERGPPGPMG**

**PPGLAGPPGESGREXXXXXXXXXXXXXXXXXXXXXXXXXXXXXXXXXXXXXXXXXXXXXX**

**XXXXXXXXGPAGPAGPVGPAGARGPAGPQGPRGDKGETGEQGDRGIKGHRGFSGLQGPPG**

**PPGSPGEQGPSGASGPAGPRGPPGSAGSPGKDGLNGLPGPIGXXXXXXXXXXXXXXXXXX**

**XXXXXXXXXXXXXXXXXXXXXXXXXXXXXXXXXXXXXRQYDG-KG-A-GPGPMGLMGPRG**

**PPGASGPPGPPGFQGPAGEPGEPGQTGPAGSRGPPGPPGKAGEDGHPGKPGRPGERGVVG**

**PQGARGFPGTPGLPGFKGIRGHNGLDGLKGQPGAPGVKGEPGAPGENGTPGQTGARGLPG**

**ERGRVGAAGPSGARGSDGSVGPVGPAGPIGAAGPPGFPGAPGPKGELGPVGNPGPTGPAG**

**PRGEVGLPGVSGPVGPPGNPGANGLAGAKGAAGLPGVAGAPGLPGPRGIPGPVGAAGATG**

**ARGLVGEPGPPGSKGESGSKGEPGSAGAQGPPGPSGEEGKRGPNGEGGATGPPGPPGLRG**

**SPGSRGLPGADGRAGVMGPPGSRGASGPAGVRGPSGDAGRPGEPGLMGPRGLPGSPGNVG**

**PAGKEGPAGLPGIDGRPGPIGPAGARGEPGNIGFPGPKGPTGDPGKAGEKGHAGLAGPRG**

**APGPDGNNGAQGPPGPQGVQGGKGEQGPAGPPGFQGLPGPAGPAGEAGKPGERXXXXXXX**

**XXXXXXXXGERGPPGQSGAAGPTGPIGSRGPSGPPGPDGNKGEPGVVGAPGTAGPSGPSG**

**LPGERGAAGIPGGKGEKGETGLRGDAGNTGRDGARGAPGAVGAPGPAGATGDRGEAGPAG**

**PAGPAGPRGSPGERGEVGPAGPNGFAGPAGAAGQPGAKGERGTKGPKGENGPVGPPGPVG**

**AAGPAGPNGPPGPAGGRGDGGPPGATGFPGAAGRTGPPGPSXXXXXXXXXXXXXXXXXXX**

**XXXXXGPSGRTGETGASGPPGFAGEKGPPGEPGTAGPPGSPGPQGLLGAPGILGLPGSRG**

**ERGLPGVAGAVGEPGPLGIAGPAGARGPPGNVGSPGVNGAPGEAGRDGNPGNDGPPGRDG**

**LPGHKGERGYPGNIGPVGAAGAPGPQGAVGPAGKHGNRGEPGPVGSVGPVGPVGPRGPSG**

**TQGIRGDKGEPGDKGPRGLPGLKGHNGLQGLPGLAGQHGDQGAPGSVGPAGPRGPAGPTG**

**PAGKDGRSGHPGPVGPAGVRGSQGSQGPSGPPGPPGPPGPPGASGGGYDLGYDGDFYRA**

**>Dasypus**

**QFSYGYDEKSAGGVSVPGPMGPSGPRGLPGPPGSPGPQGFQGPPGEPGEPGSSGPMGPRG**

**PPGPPGKNGDDGEAGKPGRPGERGPPGPQGARGLPGTAGLPGMKGHRGFSGLDGAKGDAG**

**PAGPKGEPGSPGENGAPGQMXXXXXXXXXXXXXXXXXXXXXXXXXXXXXXXXXXXXXXXX**

**XXXXXXXXXXXGEAGPQGARGSEGPQGVRGEPGPPGPAGAAGPAGNPGADGQPGAKGANG**

**APGIAGAPGFPGARGPSGPQGPSGAPGPKGNSGEPGAPGNKGDTGAKGEPGPTGIQGPPG**

**PAGEEGKRGARGEPGPTGLPGAPGERGGPGSRGFPGADGIAGPKGPAGERGSPGPAGPKG**

**SPGEAGRPGEAGLPGAKGLTGSPGSPGPDGKTGPPGPAGQDGRPGPAGPPGARGQAGVMG**

**FPGPKGAAXXXXXXXXXXXXXXXXXXXXXXXXXXXXXXXXXXXXXXXXXXXXXXXXXXXX**

**XXGLPGPAGPPGEAGKPGEQGVPGDLGAPGPSGARGERGFPGERGVQGPPGPAGPRGANG**

**APGNDGAKGDAGAPGAPGSQGAPGLQGMPGERGAAGLPGPKGDRXXXXXXXXXXXXXXXX**

**XXXXXXXXXXXXXXXXXXXXGETGPSGPAGPTGARGAPGDRGEPGPPGPAGFAGPPGADG**

**QPGAKGEPGDAGAKGDAGPPGPAGAAGPPGPIGNVGAPGPKGARGSAGPPGATGFPGAAG**

**RVGPPGPSGNAGPPGPPGPVGKEGGKGPRGETGPAGRPGEVGPPGPPGPSGEKGSPGADG**

**PAGAPGTPGPQGIAGQRGVVGLPGQRGERGFPGLPGPSGEPGKQGPSGSSGERGPPGPMG**

**PPGLAGPPGEAGREGSPGAEGSPGRDGSPGPKGDRGETGPAGPPGAPGAPGAPGPVGPAG**

**KSGDRGETGPSGPAGPAGPAGARGPSGPQGPRGDKGETGEQGDRGIKGHRGFSGLQGPAG**

**PPXXXXXXXXXXXXXXXXXXGPPGSAGTPGKDGLNGLPGPIGPPGPRGRTGDAGPVGPPG**

**PPGPPGPPGPPSGGFDFSFLPQPPQEKGHDGGRYYRARQYDG-KGVGLGPGPMGLMGPRG**

**PPGASGAPGPQGFQGPAGEPGEPGQTGPAGARGPAGPPGKAGEDGHPGKPGRPGERGVVG**

**PQGARGFPGTPGLPGFKGIRGHNGLDGLKGQAGAPGVKGEPGAPGENGTPGQTGARGLPG**

**ERGRVGAPGPAGARGSDGSVGXXGPAGPIGSAGPPGFPGAPGPKGELGPVGNPGPAGPAG**

**PRGEQGLPGVSGPVGPPXXXXXXXXXXXXXXXGLPGVAGAPGLPGPRGIPGPVGAVGATG**

**ARGLVGEPGPAGSKGESGNKGEPGSAGPQGPPGPSGEEGKRGANGEAGSTGPSGPPGLRG**

**GPGSRGLPGADGRAGVMGPAGSRGASGPAGVRGPNGDPGRPGEPGLMGPRGLPGSPGNVG**

**PAGKEGPVGLPGIDGRPGPVGPAGPRGEAGNIGFPGPKGPTGDPGKVGEKGHAGLAGNRG**

**APGPDGNNGAQGPPGLQGVQGGKGEQGPAGPPGFQGLPGPAGTTGEVGKPGERGLHGEFG**

**LPGPAGPRGERGPPGESGAAGPVGSIGSRGPSGPPGPDGNKGEPGVVGAPGTAGPSGSGG**

**LPGERGGAGIPGGKGEKGETGLRGEVGTTGRDGARGAPGAIGAPGPAGATGDRGEAGAAG**

**PAGPSGPRGTPGERGEVGPAGPNGFAGPAGAAGQPGAKGERGTKGPKGENGIAGPTGPVG**

**AAGPSGPNGAPGPAGGRGDGGPPGVTGFPGAAGRTGPPGPSGITGPPGPPGAAGKEGLRG**

**PRGDQGPVGRTGETGAGGPPGFAGEKGPSGEPGTAGPPGTAGPQGLLGAPGILGLPGSRG**

**ERGLPGVAGAVGEPGPLGISGPPGARGPSGAVGSPGVNGAPGETGRDGNPGNDGPPGRDG**

**LPGHKGERGYAGNAGPVGAAGAPGPHGSVGPAGKHGNRGEPGPVGPVGPVGAVGPRGPSG**

**PQGVRGDKGEPGEKGPRGLPGLKGHNGLQGLPGLAGQHGDQGSPGPVGPAGPRGPAGPSG**

**PAGKDGRTGHPGAVGPAGIRGSQGSQGPSGPAGPPGPPGPPGASGGGYDFGYEGDFYRA**

**>Mustela**

**QMSYGYDEKSTGGISVPGPMGPSGPRGLPGPPGAPGPQGFQGPPGEPGEPGASGPMGPRG**

**PPGPPGKNGDDGEAGKPGRPGERGPPGPQGARGLPGTAGLPGMKGHRGFSGLDGAKGDAG**

**PAGPKGEPGSPGENGAPGQMGPRGLPGERGRPGAPGPAGARGNDGATGAAGPPGPTGPAG**

**PPGFPGAVGAKGEAGPQGARGSEGPQGVRGEPGPPGPAGAAGPAGNPGADGQPGAKGANG**

**APGIAGAPGFPGARGPSGPQGPSGPPGPKGNSGEPGAPGNKGDTGAKGEPGPTGIQGPPG**

**PAGEEGKRGARGEPGPTGLPGPPGERGGPGSRGFPGADGVAGPKGPAGERGSPGPAGPKG**

**SPGEAGRPGEAGLPGAKGLTGSPGSPGPDGKTGPPGPAGQDGRPGPPGPPGARGQAGVMG**

**FPGPKGAAGEPGKAGERGVPGPPGAVGPAGKDGEAGAQGAPGPAGPAGERGEQGPAGSPG**

**FQGLPGPAGPPGEAGKPGEQGVPGDLGAPGPSGARGERGFPGERGVQGPPGPAGPRGANG**

**APGNDGAKGDAGAPGAPGSQGAPGLQGMPGERGAAGLPGPKGDRGDAGPKGADGSPGKDG**

**VRGLTGPIGPPGPAGAPGDKGEAGPSGPAGPTGARGAPGDRGEPGPPGPAGFAGPPGADG**

**QPGAKGEPGDAGAKGDAGPPGPAGPTGPPGPIGNVGAPGPKGARGSAGPPGATGFPGAAG**

**RVGPPGPSGNAGPPGPPGPAGKEGGKGPRGETGPAGRPGEVGPPGPPGPAGEKGSPGADG**

**PAGAPGTPGPQGIAGQRGVVGLPGQRGERGFPGLPGPSGEPGKQGPSGASGERGPPGPMG**

**PPGLAGPPGESGREGSPGAEGSPGRDGSPGPKGDRGETGPAGPPGAPGAPGAPGPVGPAG**

**KSGDRGETGPAGPAGPIGPVGARGPTGPQGPRGDKGETGEQGDRGIKGHRGFSGLQGPPG**

**PPGSPGEQGPSGASGPAGPRGPPGSAGSPGKDGLNGLPGPIGPPGPRGRTGDAGPVGPPG**

**PPGPPGPPGPPSGGFDFSFLPQPPQEKAHDGGRYYRARQYDG-KGVGLGPGPMGLMGPRG**

**PPGASGAPGPQGFQGPAGEPGEPGQTGPAGARGPPGPPGKAGEDGHPGKPGRPGERGVVG**

**PQGARGFPGTPGLPGFKGIRGHNGLDGLKGQPGAPGVKGEPGAPGENGTPGQTGARGLPG**

**ERGRVGAPGPAGARGSDGSVGPVGPAGPIGSAGPPGFPGAPGPKGELGPVGNPGPAGPAG**

**PRGEVGLPGVSGPVGPPGNPGANGLTGAKGAAGLPGVAGAPGLPGPRGIPGPVGAAGATG**

**ARGLVGEPGPAGSKGESGNKGEPGSAGPQGPPGPSGEEGKRGPNGEAGSAGPSGPPGLRG**

**SPGSRGLPGADGRAGVMGPPGPRGATGPAGVRGPNGDSGRPGEPGLMGPRGFPGAPGNIG**

**PAGKEGPMGLPGIDGRPGPIGPAGARGEPGNIGFPGPKGPTGDPGKPGEKGHAGLAGARG**

**APGPDGNNGAQGPPGPQGVQGGKGEQGPAGPPGFQGLPGPAGTAGEVGKPGERGLPGEFG**

**LPGPAGPRGERGPPGESGAAGPSGPIGSRGPSGPPGPDGNKGEPGVLGAPGTAGPSGPGG**

**LPGERGAAGVPGGKGEKGETGLRGEVGNPGRDGARGAPGAVGAPGPAGATGDRGEAGPAG**

**PAGPAGPRGSPGERGEVGPAGPNGFAGPAGAAGQPGAKGERGTKGPKGENGPVGPTGPVG**

**SAGPSGPNGPPGPAGSRGDGGPPGATGFPGAAGRTGPPGPSGITGPPGPPGAAGKEGLRG**

**PRGDQGPVGRTGETGAHGPPGFAGEKGPSGEPGTAGPPGTPGPQGLLGAPGILGLPGSRG**

**ERGLPGVSGSVGEPGPLGIAGPPGARGPPGAVGAPGVNGAPGEAGRDGNPGNDGPPGRDG**

**QPGHKGERGYPGNIGPVGAVGAPGPHGPVGPTGKHGNRGEPGPAGSVGPVGAAGPRGPSG**

**PQGVRGDKGEPGDKGPRGLPGLKGHNGLQGLPGLAGQHGDQGAPGSVGPAGPRGPAGPSG**

**PAGKDGRTGHPGTVGPAGIRGSQGSQGPAGPPGPPGPPGPPGPSGGGYDFGYEGDFYRA**

**>Pan**

**QLSYGYDEKSTGGISVPGPMGPSGPRGLPGPPGAPGPQGFQGPPGEPGEPGASGPMGPRG**

**PPGPPGKNGDDGEAGKPGRPGERGPPGPQGARGLPGTAGLPGMKGHRGFSGLDGAKGDAG**

**PAGPKGEPGSPGENGAPGQMGPRGLPGERGRPGAPGPAGARGNDGATGAAGPPGPTGPAG**

**PPGFPGAVGAKGEAGPQGPRGSEGPQGVRGEPGPPGPAGAAGPAGNPGADGQPGAKGANG**

**APGIAGAPGFPGARGPSGPQGPGGPPGPKGNSGEPGAPGSKGDTGAKGEPGPVGVQGPPG**

**PAGEEGKRGARGEPGPTGLPGPPGERGGPGSRGFPGADGVAGPKGPAGERGSPGPAGPKG**

**SPGEAGRPGEAGLPGAKGLTGSPGSPGPDGKTGPPGPAGQDGRPGPPGPPGARGQAGVMG**

**FPGPKGAAGEPGKAGERGVPGPPGAVGPAGKDGEAGAQGPPGPAGPAGERGEQGPAGSPG**

**FQGLPGPAGPPGEAGKPGEQGVPGDLGAPGPSGARGERGFPGERGVQGPPGPAGPRGANG**

**APGNDGAKGDAGAPGAPGSQGAPGLQGMPGERGAAGLPGPKGDRGDAGPKGADGSPGKDG**

**VRGLTGPIGPPGPAGAPGDKGESGPSGPAGPTGARGAPGDRGEPGPPGPAGFAGPPGADG**

**QPGAKGEPGDAGAKGDAGPPGPAGPAGPPGPIGNVGAPGAKGARGSAGPPGATGFPGAAG**

**RVGPPGPSGNAGPPGPPGPAGKEGGKGPRGETGPAGRPGEVGPPGPPGPAGEKGSPGADG**

**PAGAPGTPGPQGIAGQRGVVGLPGQRGERGFPGLPGPSGEPGKQGPSGASGERGPPGPMG**

**PPGLAGPPGESGREGAPGAEGSPGRDGSPGAKGDRGETGPAGPPGAPGAPGAPGPVGPAG**

**KSGDRGETGPAGPAGPVGPVGARGPAGPQGPRGDKGETGEQGDRGIKGHRGFSGLQGPPG**

**PPGSPGEQGPSGASGPAGPRGPPGSAGAPGKDGLNGLPGPIGPPGPRGRTGDAGPVGPPG**

**PPGPPGPPGPPSAGFDFSFLPQPPQEKAHDGGRYYRARQYDG-KGVGLGPGPMGLMGPRG**

**PPGAAGAPGPQGFQGPAGEPGEPGQTGPAGARGPAGPPGKAGEDGHPGKPGRPGERGVVG**

**PQGARGFPGTPGLPGFKGIRGHNGLDGLKGQPGAPGVKGEPGAPGENGTPGQTGARGLPG**

**ERGRVGAPGPAGARGSDGSVGPVGPAGPIGSAGPPGFPGAPGPKGELGAVGNAGPAGPAG**

**PRGEVGLPGLSGPVGPPGNPGANGLTGAKGAAGLPGVAGAPGLPGPRGIPGPVGAAGATG**

**ARGLVGEPGPAGSKGESGNKGEPGSAGPQGPPGPSGEEGKRGPNGEAGSAGPPGPPGLRG**

**SPGSRGLPGADGRAGVMGPAGSRGASGPAGVRGPNGDAGRPGEPGLMGPRGLPGSPGNIG**

**PAGKEGPVGLPGIDGRPGPIGPAGARGEPGNIGFPGPKGPTGDPGKNGDKGHAGLAGARG**

**APGPDGNNGAQGPPGPQGVQGGKGEQGPAGPPGFQGLPGPSGPTGEVGKPGERGLHGEFG**

**LPGPAGPRGERGPPGESGAAGPTGPIGSRGPSGPPGPDGNKGEPGVVGAVGTAGPSGPSG**

**LPGERGAAGIPGGKGEKGEPGLRGEIGNPGRDGARGAPGAVGAPGPAGATGDRGEAGAAG**

**PAGPAGPRGSPGERGEVGPAGPNGFAGPAGAAGQPGAKGERGAKGPKGENGVVGPTGPVG**

**AAGPAGPNGPPGPAGSRGDGGPPGMTGFPGAAGRTGPPGPSGISGPPGPPGPAGKEGLRG**

**PRGDQGPVGRTGEVGAVGPPGFAGEKGPSGEAGTAGPPGTPGPQGLLGAPGILGLPGSRG**

**ERGLPGVAGAVGEPGPLGIAGPPGARGPPGAVGSPGVNGAPGEAGRDGNPGNDGPPGRDG**

**QPGHKGERGYPGNIGPVGAAGAPGPHGPVGPAGKHGNRGETGPSGPVGPAGAVGPRGPSG**

**PQGIRGDKGEPGEKGPRGLPGLKGHNGLQGLPGLAGHHGDQGAPGSVGPAGPRGPAGPSG**

**PAGKDGRTGHPGTVGPAGIRGPQGHQGPAGPPGPPGPPGPPGVSGGGYDFGYDGDFYRA**

**>Nomascus**

**QLSYGYDEKSAGGISVPGPMGPSGPRGLPGPPGAPGPQGFQGPPGEPGEPGASGPMGPRG**

**PPGPPGKNGDDGEAGKPGRPGERGPPGPQGARGLPGTAGLPGMKGHRGFSGLDGAKGDAG**

**PAGPKGEPGSPGENGAPGQMGPRGLPGERGRPGAPGPAGARGNDGATGAAGPPGPTGPAG**

**PPGFPGAVGAKGEAGPQGPRGSEGPQGVRGEPGPPGPAGAAGPAGNPGADGQPGAKGANG**

**APGIAGAPGFPGARGPSGPQGPGGPPGPKGNSGEPGAPGSKGDTGAKGEPGPVGVQGPPG**

**PAGEEGKRGARGEPGPTGLPGPPGERGGPGSRGFPGADGVAGPKGPAGERGSPGPAGPKG**

**SPGEAGRPGEAGLPGAKGLTGSPGSPGPDGKTGPPGPAGQDGRPGPPGPPGARGQAGVMG**

**FPGPKGAAGEPGKAGERGVPGPPGAVGPAGKDGEAGAQGPPGPAGPAGERGEQGPAGSPG**

**FQGLPGPAGPPGEAGKPGEQGVPGDLGAPGPSGARGERGFPGERGVQGPPGPAGPRGANG**

**APGNDGAKGDAGAPGAPGSQGAPGLQGMPGERGAAGLPGPKGDRGDAGPKGADGSPGKDG**

**VRGLTGPIGPPGPAGAPGDKGETGPSGPAGPTGARGAPGDRGEPGPPGPAGFAGPPGADG**

**QPGAKGEPGDAGAKGDAGPPGPAGPAGPPGPIGNVGAPGAKGARGSAGPPGATGFPGAAG**

**RVGPPGPSGNAGPPGPPGPAGKEGGKGPRGETGPAGRPGEVGPPGPPGPAGEKGSPGADG**

**PAGAPGTPGPQGIAGQRGVVGLPGQRGERGFPGLPGPSGEPGKQGPSGASGERGPPGPMG**

**PPGLAGPPGESGREGAPGAEGSPGRDGSPGPKGDRGETGPAGPPGAPGAPGAPGPVGPAG**

**KSGDRGETGPAGPAGPVGPVGARGPAGPQGPRGDKGETGEQGDRGIKGHRGFSGLQGPPG**

**PPGSPGEQGPSGASGPAGPRGPPGSAGAPGKDGLNGLPGPIGPPGPRGRTGDAGPVGPPG**

**PPGPPGPPGPPSAGFDFSFLPQPPQEKAHDGGRYYRARQYDG-KGVGLGPGPMGLMGPRG**

**PPGAAGAPGPQGFQGPAGEPGEPGQTGPAGARGPAGPPGKAGEDGHPGKPGRPGERGVVG**

**PQGARGFPGTPGLPGFKGIRGHNGLDGLKGQPGAPGVKGEPGAPGENGTPGQTGARGLPG**

**ERGRVGAPGPAGARGSDGSVGPVGPAGPIGSAGPPGFPGAPGPKGELGAVGNAGPAGPAG**

**PRGEVGLPGLSGPVGPPGNPGANGLTGAKGAAGLPGVAGAPGLPGPRGIPGPVGAAGATG**

**ARGLVGEPGPAGSKGESGNKGEPGSAGPQGPPGPSGEEGKRGPNGEAGSAGPPGPPGLRG**

**SPGSRGLPGADGRAGVMGPPGSRGASGPAGVRGPNGDAGRPGEPGLMGPRGLPGSPGNIG**

**PAGKEGPVGLPGIDGRPGPIGPAGARGEAGNIGFPGPKGPTGDPGKSGDKGHAGLAGARG**

**APGPDGNNGAQGPPGPQGVQGGKGEQGPAGPPGFQGLPGPSGPAGEVGKPGERGLHGEFG**

**LPGPAGPRGERGPPGESGAAGPTGPIGSRGPSGPPGPDGNKGEPGVVGAVGTAGPSGPSG**

**LPGERGAAGIPGGKGEKGEPGLRGEIGNPGRDGARGAPGAVGAPGPAGATGDRGEAGAAG**

**PAGPAGPRGSPGERGEVGPAGPNGFAGPAGAAGQPGAKGERGAKGPKGENGVVGPTGPVG**

**AAGPAGPNGPPGPAGSRGDGGPPGMTGFPGAAGRTGPPGPSGISGPPGPPGPAGKEGLRG**

**PRGDQGPVGRTGEVGAVGPPGFAGEKGPSGEAGTAGPPGTPGPQGLLGAPGILGLPGSRG**

**ERGLPGVAGAVGEPGPLGIAGPPGARGPPGAVGSPGVNGAPGEAGRDGNPGNDGPPGRDG**

**QPGHKGERGYPGNIGPVGAAGAPGPHGPVGPAGKHGNRGETGPSGPVGPAGAVGPRGPSG**

**PQGIRGDKGEPGDKGPRGLPGLKGHNGLQGLPGLAGHHGDQGAPGSVGPAGPRGPAGPSG**

**PAGKDGRTGHPGTVGPAGIRGPQGHQGPAGPPGPPGPPGPPGVSGGGYDFGYDGDFYRA**

**>Macaca**

**QLSYGYDEKSTGGISVPGPMGPSGPRGLPGPPGAPGPQGFQGPPGEPGEPGASGPMGPRG**

**PPGPPGKNGDDGEAGKPGRPGERGPPGPQGARGLPGTAGLPGMKGHRGFSGLDGAKGDAG**

**PAGPKGEPGSPGENGAPGQMGPRGLPGERGRPGAPGPAGARGNDGATGAAGPPGPTGPAG**

**PPGFPGAVGAKGEAGPQGPRGSEGPQGVRGEPGPPGPAGAAGPAGNPGADGQPGAKGANG**

**APGIAGAPGFPGARGPSGPQGPGGPPGPKGNSGEPGAPGSKGDTGAKGEPGPVGVQGPPG**

**PAGEEGKRGARGEPGPTGLPGPPGERGGPGSRGFPGADGVAGPKGPAGERGSPGPAGPKG**

**SPGEAGRPGEAGLPGAKGLTGSPGSPGPDGKTGPPGPAGQDGRPGPPGPPGARGQAGVMG**

**FPGPKGAAGEPGKAGERGVPGPPGAVGPAGKDGEAGAQGPPGPAGPAGERGEQGPAGSPG**

**FQGLPGPAGPPGEAGKPGEQGVPGDLGAPGPSGARGERGFPGERGVQGPPGPAGPRGANG**

**APGNDGAKGDAGAPGAPGSQGAPGLQGMPGERGAAGLPGPKGDRGDAGPKGADGSPGKDG**

**VRGLTGPIGPPGPAGAPGDKGETGPSGPAGPTGARGAPGDRGEPGPPGPAGFAGPPGADG**

**QPGAKGEPGDAGAKGDAGPPGPAGPAGPPGPIGNVGAPGPKGARGSAGPPGATGFPGAAG**

**RVGPPGPSGNAGPPGPPGPAGKEGGKGPRGETGPAGRPGEVGPPGPPGPAGEKGSPGADG**

**PAGAPGTPGPQGIAGQRGVVGLPGQRGERGFPGLPGPSGEPGKQGPSGASGERGPPGPMG**

**PPGLAGPPGESGREGAPGAEGSPGRDGSPGAKGDRGETGPAGPPGAPGAPGAPGPVGPAG**

**KSGDRGETGPAGPAGPVGPVGARGPAGPQGPRGDKGETGEQGDRGIKGHRGFSGLQGPPG**

**PPGSPGEQGPSGASGPAGPRGPPGSAGTPGKDGLNGLPGPIGPPGPRGRTGDAGPVGPPG**

**PPGPPGPPGPPSGGFDFSFLPQPPQEKAHDGGRYYRARQYDG-KGVGLGPGPMGLMGPRG**

**PPGAAGAPGPQGFQGPAGEPGEPGQTGPAGSRGPAGPPGKAGEDGHPGKPGRPGERGVVG**

**PQGARGFPGTPGLPGFKGIRGHNGLDGLKGQPGAPGVKGEPGAPGENGTPGQTGARGLPG**

**ERGRVGAPGPAGARGSDGSVGPVGPAGPIGSAGPPGFPGAPGPKGELGAVGNAGPAGPAG**

**PRGEVGLPGLSGPVGPPGNPGANGLTGAKGAAGLPGVAGAPGLPGPRGIPGPVGAAGATG**

**ARGLVGEPGPAGSKGESGNKGEPGSAGPQGPPGPSGEEGKRGPNGEVGSAGPPGPPGLRG**

**SPGSRGLPGADGRAGVMGPPGSRGASGPAGVRGPNGDAGRPGEPGLMGPRGLPGSPGNIG**

**PAGKEGPVGLPGIDGRPGPIGPAGARGEPGNIGFPGPKGPTGDPGKNGDKGHAGLAGARG**

**APGPDGNNGAQGPPGPQGVQGGKGEQGPAGPPGFQGLPGPSGPAGEVGKPGERGLPGEFG**

**LPGPAGARGERGPPGESGAAGPTGPIGSRGPSGPPGPDGNKGEPGVVGAAGTAGPSGPSG**

**LPGERGAAGIPGGKGEKGEPGLRGEIGNPGRDGARGAPGAVGAPGPAGATGDRGEAGAAG**

**PAGPAGPRGSPGERGEVGPAGPNGFAGPAGAAGQPGAKGERGAKGPKGENGVVGPTGPVG**

**AAGPSGPNGPPGPAGSRGDGGPPGMTGFPGAAGRTGPPGPSGISGPPGPPGPSGKEGLRG**

**PRGDQGPVGRTGEVGAVGPPGFAGEKGPSGEAGTAGPPGTPGPQGLLGAPGILGLPGSRG**

**ERGLPGVAGVVGEPGPLGIAGPPGARGPPGAVGSPGVNGAPGEAGRDGNPGNDGPPGRDG**

**QPGHKGERGYPGNNGPVGAAGAPGPHGPVGPAGKHGNRGETGPSGPVGPAGAVGPRGPSG**

**PQGIRGDKGEPGDKGPRGLPGLKGHNGLQGLPGLAGHHGDQGAPGSVGPAGPRGPAGPSG**

**PAGKDGRTGHPGTVGPAGIRGPQGHQGPAGPPGPPGPPGPPGVSGGGYDFGYDGDFYRA**

**>Otolemur**

**QMSYGYDEKSAG-VSVPGPMGPSGPRGLPGPPGAPGPQGFQGPPGEPGEPGSAGPMGPRG**

**PPGPPGKNGDDGEAGKPGRPGERGPPGPQGARGLPGTAGLPGMKGHRGFSGLDGAKGDAG**

**APGPKGEPGSPGENGAPGQMGPRGLPGERGRPGPSGPAGARGNDGATGAAGPPGPTGPAG**

**PPGFPGAAGAKGEAGPQGARGSEGPQGVRGEPGPPGPAGAAGPAGNPGADGQPGAKGANG**

**APGIAGAPGFPGARGPSGPQGPSGPPGPKGNSGEPGAPGNKGDTGAKGEPGPAGVQGPPG**

**PAGEEGKRGARGEPGPTGLPGPPGERGGPGSRGFPGADGVAGPKGPAGERGSPGPAGPKG**

**SPGEAGRPGEAGLPGAKGLTGSPGSPGPDGKTGPPGPAGQDGRPGPPGPPGARGQAGVMG**

**FPGPKGAAGEPGKAGERGVPGPTGAVGAPGKDGEAGAQGPPGPAGPAGERGEQGPAGSPG**

**FQGLPGPAGPPGEAGKPGEQGVPGDLGAPGPSGARGERGFPGERGVQGPPGPAGPRGGNG**

**APGNDGAKGDAGAPGAPGSQGAPGLQGMPGERGAAGLPGPKGDRGDAGPKGADGSPGKDG**

**ARGLTGPIGPPGPAGAPGDKGESGPSGPAGPTGARGAPGDRGEPGPPGPAGFAGPPGADG**

**QPGAKGEPGDAGAKGDAGPAGPAGPAGPPGPVGNVGAPGPKGARGSAGPPGATGFPGAAG**

**RVGPPGPSGNAGPPGPPGPAGKEGSKGPRGETGPAGRTGEVGPPGPPGPAGEKGSPGADG**

**PAGAPGTPGPQGIAGQRGVVGLPGQRGERGFPGLPGPSGEPGKQGPSGASGERGPPGPMG**

**PPGLAGPPGESGREGAPGAEGSPGRDGAPGPKGDRGETGPAGPPGAPGAPGAPGPVGPAG**

**KSGDRGETGPSGPAGPVGPAGARGPAGPQGPRGDKGETGEQGERGIKGHRGFSGLQGPPG**

**PPGSPGEQGPSGASGPAGPRGPPGSAGAAGKDGLNGLPGPIGPPGPRGRTGDAGPVGPAG**

**PPGPPGPPGPPSGGFDFSFLPQPPQEKAQDSGRYYRARQYDG-KAAGLGPGPMGLMGPRG**

**PPGASGAPGPQGFQGPAGEPGEPGQTGPAGARGPAGAPGKAGEDGHPGKPGRPGERGVVG**

**PQGARGFPGTPGLPGFKGIRGHSGPDGLKGQAGLPGAKGEPGSPGENGTPGQTGARGLPG**

**ERGRVGAPGPSGARGSDGSVGPVGPAGPVGSAGPPGFPGAPGPKGELGPVGNPGPAGPAG**

**PRGEVGLPGLSGPVGPPGNPGANGLTGAKGAAGLPGVAGAPGLPGPRGIPGPVGAAGATG**

**ARGLVGEPGPAGSKGESGNKGEPGSAGPQGPPGPSGEEGKRGSNGEPGSAGPSGPPGLRG**

**SPGSRGLPGADGRGGVMGPPGNRGQSGPAGVRGPSGDSGRPGEPGLMGPRGLPGSPGNVG**

**PAGKEGPAGLPGVDGRPGPVGPAGARGEPGNIGFPGPKGPSGDPGKAGDKGHPGLAGARG**

**APGPDGNNGAQGPPGPQGVQGGKGEQGPAGPPGFQGLPGPSGPAGEVGKPGERGLHGEFG**

**LPGPAGPRGERGPPGESGAAGPSGPIGSRGPSGPPGPDGNKGEPGVVGAPGTAGPSGPSG**

**LPGERGAAGMPGGKGEKGETGPRGEMGTTGRDGARGAPGAVGAPGPAGATGDRGEAGAAG**

**PAGPAGPRGSPGERGEVGPAGPNGFAGPAGAAGQPGAKGERGAKGPKGENGAVGPAGAVG**

**PAGPSGPNGPPGPAGGRGDGGPPGMTGFPGAAGRTGPPGPSGMSGPPGPPGPSGKEGLRG**

**PRGDQGPVGRSGETGPSGPPGFAGEKGPSGEAGAAGPPGTPGPQGLLGAPGILGLPGSRG**

**ERGLPGVAGAVGEPGPLGVAGPPGARGPSGGVGNPGVNGAPGEAGRDGNPGNDGPPGRDG**

**QPGHKGERGYPGNVGPAGAVGAPGSHGPVGPAGKHGNRGEPGAVGPVGPTGAVGPRGPSG**

**AQGVRGDKGEPGDKGPRGLPGLKGHGGLQGLPGLAGHHGDQGAPGSVGPAGPRGPAGPSG**

**PVGKDGRNGHPGTVGPAGVRGPQGHQGPAGPPGPPGPPGPPGASGGGYDFGYDGDFYRA**

**>Rattus**

**QMSYGYDEKSAG-VSVPGPMGPSGPRGLPGPPGAPGPQGFQGPPGEPGEPGASGPMGPRG**

**PPGPPGKNGDDGEAGKPGRPGERGPPGPQGARGLPGTAGLPGMKGHRGFSGLDGAKGDTG**

**PAGPKGEPGSPGENGAPGQMGPRGLPGERGRPGPPGSAGARGNDGAVGAAGPPGPTGPTG**

**PPGFPGAAGAKGEAGPQGARGSEGPQGVRGEPGPPGPAGAAGPAGNPGADGQPGAKGANG**

**APGIAGAPGFPGARGPSGPQGPSGAPGPKGNSGEPGAPGNKGDTGAKGEPGPAGVQGPPG**

**PAGEEGKRGARGEPGPSGLPGPPGERGGPGSRGFPGADGVAGPKGPAGERGSPGPAGPKG**

**SPGEAGRPGEAGLPGAKGLTGSPGSPGPDGKTGPPGPAGQDGRPGPAGPPGARGQAGVMG**

**FPGPKGTAGEPGKAGERGVPGPPGAVGPAGKDGEAGAQGAPGPAGPAGERGEQGPAGSPG**

**FQGLPGPAGPPGEAGKPGEQGVPGDLGAPGPSGARGERGFPGERGVQGPPGPAGPRGNNG**

**APGNDGAKGDTGAPGAPGSQGAPGLQGMPGERGAAGLPGPKGDRGDAGPKGADGSPGKDG**

**VRGLTGPIGPPGPAGAPGDKGETGPSGPAGPTGARGAPGDRGEPGPPGPAGFAGPPGADG**

**QPGAKGEPGDTGVKGDAGPPGPAGPAGPPGPIGNVGAPGPKGSRGAAGPPGATGFPGAAG**

**RVGPPGPSGNAGPPGPPGPVGKEGGKGPRGETGPAGRPGEVGPPGPPGPAGEKGSPGADG**

**PAGSPGTPGPQGIAGQRGVVGLPGQRGERGFPGLPGPSGEPGKQGPSGASGERGPPGPMG**

**PPGLAGPPGESGREGSPGAEGSPGRDGAPGAKGDRGETGPAGPPGAPGAPGAPGPVGPAG**

**KNGDRGETGPAGPAGPIGPAGARGPAGPQGPRGDKGETGEQGDRGIKGHRGFSGLQGPPG**

**SPGSPGEQGPSGASGPAGPRGPPGSAGSPGKDGLNGLPGPIGPPGPRGRTGDSGPAGPPG**

**PPGPPGPPGPPSGGYDFSFLPQPPQEKSQDGGRYYRARQYSD-KGVSAGPGPMGLMGPRG**

**PPGAVGAPGPQGFQGPAGEPGEPGQTGPAGSRGPAGPPGKAGEDGHPGKPGRPGERGVVG**

**PQGARGFPGTPGLPGFKGIRGHNGLDGLKGQPGAQGVKGEPGAPGENGTPGQAGARGLPG**

**ERGRVGAPGPAGARGSDGSVGPVGPAGPIGSAGPPGFPGAPGPKGELGPVGNPGPAGPAG**

**PRGEAGLPGLSGPVGPPGNPGANGLTGAKGATGLPGVAGAPGLPGPRGIPGPVGAAGATG**

**PRGLVGEPGPAGSKGETGNKGEPGSAGAQGPPGPSGEEGKRGSPGEPGSAGPAGPPGLRG**

**SPGSRGLPGADGRAGVMGPPGNRGSTGPAGVRGPNGDAGRPGEPGLMGPRGLPGSPGNVG**

**PAGKEGPVGLPGIDGRPGPIGPAGPRGEAGNIGFPGPKGPSGDPGKPGEKGHPGLAGARG**

**APGPDGNNGAQGPPGPQGVQGGKGEQGPAGPPGFQGLPGPSGTAGEVGKPGERGLPGEFG**

**LPGPAGPRGERGPPGESGAAGPSGPIGIRGPSGAPGPDGNKGEAGAVGAPGSAGASGPGG**

**LPGERGAAGIPGGKGEKGETGLRGEIGNPGRDGARGAPGAIGAPGPAGASGDRGEAGAAG**

**PSGPAGPRGSPGERGEVGPAGPNGFAGPAGSAGQPGAKGEKGTKGPKGENGIVGPTGPVG**

**AAGPSGPNGPPGPAGSRGDGGPPGMTGFPGAAGRTGPPGPSGITGPPGPPGAAGKEGLRG**

**PRGDQGPVGRTGEIGASGPPGFAGEKGPSGEPGTTGPPGTAGPQGLLGAPGILGLPGSRG**

**ERGQPGIAGALGEPGPLGIAGPPGARGPPGAVGSPGVNGAPGEAGRDGNPGSDGPPGRDG**

**QPGHKGERGYPGNIGPTGAAGAPGPHGSVGPAGKHGNRGEPGPAGSVGPVGAVGPRGPSG**

**PQGIRGDKGEPGDKGARGLPGLKGHNGLQGLPGLAGLHGDQGAPGPVGPAGPRGPAGPSG**

**PIGKDGRSGHPGPVGPAGVRGSQGSQGPAGPPGPPGPPGPPGVSGGGYDFGFEGGFYRA**

**>Mus**

**QMSYGYDEKSAG-VSVPGPMGPSGPRGLPGPPGAPGPQGFQGPPGEPGEPGGSGPMGPRG**

**PPGPPGKNGDDGEAGKPGRPGERGPPGPQGARGLPGTAGLPGMKGHRGFSGLDGAKGDAG**

**PAGPKGEPGSPGENGAPGQMGPRGLPGERGRPGPPGTAGARGNDGAVGAAGPPGPTGPTG**

**PPGFPGAVGAKGEAGPQGARGSEGPQGVRGEPGPPGPAGAAGPAGNPGADGQPGAKGANG**

**APGIAGAPGFPGARGPSGPQGPSGPPGPKGNSGEPGAPGNKGDTGAKGEPGATGVQGPPG**

**PAGEEGKRGARGEPGPSGLPGPPGERGGPGSRGFPGADGVAGPKGPSGERGAPGPAGPKG**

**SPGEAGRPGEAGLPGAKGLTGSPGSPGPDGKTGPPGPAGQDGRPGPAGPPGARGQAGVMG**

**FPGPKGTAGEPGKAGERGLPGPPGAVGPAGKDGEAGAQGAPGPAGPAGERGEQGPAGSPG**

**FQGLPGPAGPPGEAGKPGEQGVPGDLGAPGPSGARGERGFPGERGVQGPPGPAGPRGNNG**

**APGNDGAKGDTGAPGAPGSQGAPGLQGMPGERGAAGLPGPKGDRGDAGPKGADGSPGKDG**

**ARGLTGPIGPPGPAGAPGDKGEAGPSGPPGPTGARGAPGDRGEAGPPGPAGFAGPPGADG**

**QPGAKGEPGDTGVKGDAGPPGPAGPAGPPGPIGNVGAPGPKGPRGAAGPPGATGFPGAAG**

**RVGPPGPSGNAGPPGPPGPVGKEGGKGPRGETGPAGRPGEVGPPGPPGPAGEKGSPGADG**

**PAGSPGTPGPQGIAGQRGVVGLPGQRGERGFPGLPGPSGEPGKQGPSGSSGERGPPGPMG**

**PPGLAGPPGESGREGSPGAEGSPGRDGAPGAKGDRGETGPAGPPGAPGAPGAPGPVGPAG**

**KNGDRGETGPAGPAGPIGPAGARGPAGPQGPRGDKGETGEQGDRGIKGHRGFSGLQGPPG**

**SPGSPGEQGPSGASGPAGPRGPPGSAGSPGKDGLNGLPGPIGPPGPRGRTGDSGPAGPPG**

**PPGPPGPPGPPSGGYDFSFLPQPPQEKSQDGGRYYRARQYSD-KGVSSGPGPMGLMGPRG**

**PPGAVGAPGPQGFQGPAGEPGEPGQTGPAGPRGPAGSPGKAGEDGHPGKPGRPGERGVVG**

**PQGARGFPGTPGLPGFKGVRGHSGMDGLKGQPGAQGVKGEPGAPGENGTPGQAGARGLPG**

**ERGRVGAPGPAGARGSDGSVGPVGPAGPIGSAGPPGFPGAPGPKGELGPVGNPGPAGPAG**

**PRGEVGLPGLSGPVGPPGNPGTNGLTGAKGATGLPGVAGAPGLPGPRGIPGPAGAAGATG**

**ARGLVGEPGPAGSKGESGNKGEPGSVGAQGPPGPSGEEGKRGSPGEAGSAGPAGPPGLRG**

**SPGSRGLPGADGRAGVMGPPGNRGSTGPAGIRGPNGDAGRPGEPGLMGPRGLPGSPGNVG**

**PSGKEGPVGLPGIDGRPGPIGPAGPRGEAGNIGFPGPKGPSGDPGKPGERGHPGLAGARG**

**APGPDGNNGAQGPPGPQGVQGGKGEQGPAGPPGFQGLPGPSGTTGEVGKPGERGLPGEFG**

**LPGPAGPRGERGTPGESGAAGPSGPIGSRGPSGAPGPDGNKGEAGAVGAPGSAGASGPGG**

**LPGERGAAGIPGGKGEKGETGLRGDTGNTGRDGARGIPGAVGAPGPAGASGDRGEAGAAG**

**PSGPAGPRGSPGERGEVGPAGPNGFAGPAGAAGQPGAKGEKGTKGPKGENGIVGPTGSVG**

**AAGPSGPNGPPGPVGSRGDGGPPGMTGFPGAAGRTGPPGPSGIAGPPGPPGAAGKEGLRG**

**PRGDQGPVGRTGETGASGPPGFVGEKGPSGEPGTAGAPGTAGPQGLLGAPGILGLPGSRG**

**ERGLPGIAGALGEPGPLGISGPPGARGPPGAVGSPGVNGAPGEAGRDGNPGSDGPPGRDG**

**QPGHKGERGYPGSIGPTGAAGAPGPHGSVGPAGKHGNRGEPGPAGSVGPVGAVGPRGPSG**

**PQGIRGDKGEPGDKGHRGLPGLKGYSGLQGLPGLAGLHGDQGAPGPVGPAGPRGPAGPSG**

**PVGKDGRSGQPGPVGPAGVRGSQGSQGPAGPPGPPGPPGPPGVSGGGYDFGFEGDFYRA**

**>Homo**

**QLSYGYDEKSTGGISVPGPMGPSGPRGLPGPPGAPGPQGFQGPPGEPGEPGASGPMGPRG**

**PPGPPGKNGDDGEAGKPGRPGERGPPGPQGARGLPGTAGLPGMKGHRGFSGLDGAKGDAG**

**PAGPKGEPGSPGENGAPGQMGPRGLPGERGRPGAPGPAGARGNDGATGAAGPPGPTGPAG**

**PPGFPGAVGAKGEAGPQGPRGSEGPQGVRGEPGPPGPAGAAGPAGNPGADGQPGAKGANG**

**APGIAGAPGFPGARGPSGPQGPGGPPGPKGNSGEPGAPGSKGDTGAKGEPGPVGVQGPPG**

**PAGEEGKRGARGEPGPTGLPGPPGERGGPGSRGFPGADGVAGPKGPAGERGSPGPAGPKG**

**SPGEAGRPGEAGLPGAKGLTGSPGSPGPDGKTGPPGPAGQDGRPGPPGPPGARGQAGVMG**

**FPGPKGAAGEPGKAGERGVPGPPGAVGPAGKDGEAGAQGPPGPAGPAGERGEQGPAGSPG**

**FQGLPGPAGPPGEAGKPGEQGVPGDLGAPGPSGARGERGFPGERGVQGPPGPAGPRGANG**

**APGNDGAKGDAGAPGAPGSQGAPGLQGMPGERGAAGLPGPKGDRGDAGPKGADGSPGKDG**

**VRGLTGPIGPPGPAGAPGDKGESGPSGPAGPTGARGAPGDRGEPGPPGPAGFAGPPGADG**

**QPGAKGEPGDAGAKGDAGPPGPAGPAGPPGPIGNVGAPGAKGARGSAGPPGATGFPGAAG**

**RVGPPGPSGNAGPPGPPGPAGKEGGKGPRGETGPAGRPGEVGPPGPPGPAGEKGSPGADG**

**PAGAPGTPGPQGIAGQRGVVGLPGQRGERGFPGLPGPSGEPGKQGPSGASGERGPPGPMG**

**PPGLAGPPGESGREGAPGAEGSPGRDGSPGAKGDRGETGPAGPPGAPGAPGAPGPVGPAG**

**KSGDRGETGPAGPAGPVGPVGARGPAGPQGPRGDKGETGEQGDRGIKGHRGFSGLQGPPG**

**PPGSPGEQGPSGASGPAGPRGPPGSAGAPGKDGLNGLPGPIGPPGPRGRTGDAGPVGPPG**

**PPGPPGPPGPPSAGFDFSFLPQPPQEKAHDGGRYYRARQYDG-KGVGLGPGPMGLMGPRG**

**PPGAAGPPGPQGFQGPAGEPGEPGQTGPAGARGPAGPPGKAGEDGHPGKPGRPGERGVVG**

**PQGARGFPGTPGLPGFKGIRGHNGLDGLKGQPGAPGVKGEPGAPGENGTPGQTGARGLPG**

**ERGRVGAPGPAGARGSDGSVGPVGPAGPIGSAGPPGFPGAPGPKGELGAVGNAGPAGPAG**

**PRGEVGLPGLSGPVGPPGNPGANGLTGAKGAAGLPGVAGAPGLPGPRGIPGPVGAAGATG**

**ARGLVGEPGPAGSKGESGNKGEPGSAGPQGPPGPSGEEGKRGPNGEAGSAGPPGPPGLRG**

**SPGSRGLPGADGRAGVMGPPGSRGASGPAGVRGPNGDAGRPGEPGLMGPRGLPGSPGNIG**

**PAGKEGPVGLPGIDGRPGPIGPAGARGEPGNIGFPGPKGPTGDPGKNGDKGHAGLAGARG**

**APGPDGNNGAQGPPGPQGVQGGKGEQGPPGPPGFQGLPGPSGPAGEVGKPGERGLHGEFG**

**LPGPAGPRGERGPPGESGAAGPTGPIGSRGPSGPPGPDGNKGEPGVVGAVGTAGPSGPSG**

**LPGERGAAGIPGGKGEKGEPGLRGEIGNPGRDGARGAPGAVGAPGPAGATGDRGEAGAAG**

**PAGPAGPRGSPGERGEVGPAGPNGFAGPAGAAGQPGAKGERGAKGPKGENGVVGPTGPVG**

**AAGPAGPNGPPGPAGSRGDGGPPGMTGFPGAAGRTGPPGPSGISGPPGPPGPAGKEGLRG**

**PRGDQGPVGRTGEVGAVGPPGFAGEKGPSGEAGTAGPPGTPGPQGLLGAPGILGLPGSRG**

**ERGLPGVAGAVGEPGPLGIAGPPGARGPPGAVGSPGVNGAPGEAGRDGNPGNDGPPGRDG**

**QPGHKGERGYPGNIGPVGAAGAPGPHGPVGPAGKHGNRGETGPSGPVGPAGAVGPRGPSG**

**PQGIRGDKGEPGEKGPRGLPGLKGHNGLQGLPGLAGHHGDQGAPGSVGPAGPRGPAGPSG**

**PAGKDGRTGHPGTVGPAGIRGPQGHQGPAGPPGPPGPPGPPGVSGGGYDFGYDGDFYRA**

**>Ochotona**

**QMSYGYDEKSAG-VSVPGPMGPSGPRGLPGPPGSPGPQGFQGPPGEPGEPGASGPMGPRG**

**PPGPPGKNGDDGEAGKPGRPGERGPPGPQGARGLTGTAGLPGMKGHRGFSGLDGAKGDAG**

**PAGPKGEPGSPGENGAPGQMGPRGLPGERGRPGPPGTAGARGNDGATGAAGPPGPTGPAG**

**PPGFPGAVGAKGEAGPQGARGSEGPQGIRGEPGPPGPAGAAGPAGNPGADGQPGAKGANG**

**APGIAGAPGFPGARGPSGPQGPSGPPGPKGNSGEPGAPGNKGDTGAKGEPGPAGVQGPPG**

**PAGEEGKRGARGEPGPAGLPGPPGERGGPGSRGFPGADGVAGPKGPAGERGAPGPAGPKG**

**SPGEAGRPGEAGLPGAKGLTGSPGSPGPDGKTGPPGPAGQDGRPGPPGPPGARGQAGVMG**

**FPGPKGAAGEPGKAGERGVPGPPGAVGAPGKDGEAGAQGPPGPAGPAGERGEQGPAGSPG**

**FQGLPGPAGPPGEAGKPGEQGVPGDLGAPGPSGARGERGFPGERGVQGPPGPAGPRGSNG**

**APGNDGAKGDAGAPGAPGSQGAPGLQGMPGERGAAGLPGPKGDRGDAGPKGADGSPGKDG**

**VRGLTGPIGPPGPAGAPGDKGETGPSGPAGPTGARGAPGDRGEPGPPGPAGFAGPPGADG**

**QPGAKGEPGDAGAKGDAGPPGPAGPAGPPGPIGNVGAPGPKGARGSAGPPGATGFPGAAG**

**RVGPPGPSGNAGPPGPPGPAGKEGGKGPRGETGPAGRPGEVGPPGPPGPAGEKGSPGADG**

**PAGAPGTPGPQGITGQRGVVGLPGQRGERGFPGLPGPSGEPGKQGPSGASGERGPPGPMG**

**PPGLAGPPGESGREGSPGAEGSPGRDGSPGPKGDRGETGPAGPPGAPGAPGAPGPVGPAG**

**KSGDRGETGPAGPAGPIGPAGARGPAGPQGPRGDKGETGEQGDRGIKGHRGFSGLQGPPG**

**PPGSPGEQGPSGASGPAGPRGPPGSAGAPGKDGLNGLPGPIGPPGXXXXXXXXXXXXXXX**

**XXXXXXXXXXXXXXXXXXXXPQPPQEKAHDGGRYYRARXXXX?XX?X?XXXXMGLMGPRG**

**PPGAAGAPGPQGFQGPAGEPGEPGQTGPAGARGPPGAPGKAGEDGHPGKPGRPGERGIMG**

**PQGARGFPGTPGLPGFKGIRGHNGLDGLKGQPGAPGVKGEPGAPGENGTPGQTGARGLPG**

**ERGRVGAPGPAGARGSDGSVGPVGPAGPIGSAGPPGFPGAPGPKGELGPVGNPGPSGPAG**

**PRGEVGLPGVSGPVGPPGNPGTNGLTGAKGAAGLPGVAGAPGLPGPRGLPGPVGAAGATG**

**ARGLVGEPGPAGSKGESGNKGEPGSAGPQGPPGPSGEEGKRGSTGEPGSAGPPGPPGLRG**

**SPGSRGLPGADGRAGVMGPPGSRGSTGPAGVRGPNGDSGRPGEPGLVGPRGLPGSPGNVG**

**PAGKEGPVGLPGIDGRPGPIGPAGARGEPGNIGFPGPKGPSGDAGKSGDKGHPGLAGARG**

**APGPDGNNGAQGPPGPQGVQGGKGEQGPAGPPGFQGLPGPSGPAGEVGKPGERGLPGEFG**

**LPGPAGARGERGPPGESGAAGPPGPIGSRGPSGPPGPDGNKGEPGAVGAPGNAGASGPGG**

**LPGERGAAGIPGGKGEKGETGLRGEVGNPGRDGARGAPGAVGAPGPAGATGDRGEAGAAG**

**PAGPAGPRGSPGERGEVGPAGPNGFAGPAGAAGQPGAKGERGTKGPKGENGVVGPTGPVG**

**AAGPSGPNGPPGPVGGRGDGGPPGMTGFPGAAGRTGPPGPSGITGPPGPPGAAGKEGLRG**

**PRGDQGPVGRTGEPGAAGPPGFAGEKGPSGEAGTAGPPGTPGPQGLLGPPGILGLPGTRG**

**ERGLPGVAGALGEPGPLGVAGPPGARGPPGAVGSPGVNGAPGEAGRDGNPGSDGPPGRDG**

**QPGHKGERGYPGNAGPAGAAGAPGPQGSVGPTGKHGNRGEPGPAGSVGPVGAVGPRGPSG**

**PQGIRGDKGEPGDKGPRGLPGLKGHNGLQGLPGLAGQHGDQGAPGAVGPAGPRGPAGPTG**

**PAGKDGRSGHPGTVGPAGVRGSQGSQGPAGPPGPPGPPGPPGASGGGYDFGYDGDFYRA**

**>Sarcophilus**

**QMSYGYDEKSGGGMSVPGPMGPSGPRGLPGPPGSPGPQGFQGPPGEPGEPGASGPMGPRG**

**PAGPPGKNGDDGEAGKPGRPGERGPPGPQGARGLPGTAGLPGMKGHRGFSGLDGAKGDSG**

**PAGPKGEPGSPGENGAPGQMGPRGLPGERGRPGPPGPAGARGNDGATGAAGPPGPTGPAG**

**PPGFPGAVGAKGEAGPQGSRGSEGPQGVRGEPGPPGPAGSPGPSGNPGADGQPGAKGANG**

**APGIAGAPGFPGARGPSGPQGPSGAPGPKGNSGEPGTPGNKGDPGAKGEPGPVGVQGPPG**

**PAGEEGKRGSRGEPGPAGLPGPAGERGGPGSRGFPGADGVAGPKGAPGERGAPGPAGPKG**

**SPGESGRPGEAGLPGAKGLTGSPGSPGPDGKTGPPGPAGQDGRPGPPGPPGARGQAGVMG**

**FPGPKGAAGEPGKAGERGVPGPPGAVGPAGKDGEAGAQGAPGPAGPAGERGEQGPAGSPG**

**FQGLPGPAGPPGEAGKPGEQGVPGDAGAPGPSGARGERGFPGERGVQGPPGPQGPRGANG**

**APGNDGAKGDAGAPGAPGGQGPPGLQGMPGERGAAGLPGAKGDRGDAGPKGADGAPGKDG**

**VRGLTGPIGPPGPAGPSGDKGESGPSGPAGPTGARGAPGERGEPGPPGPAGFAGPPGADG**

**QPGAKGEPGDAGAKGDAGPPGPAGPTGAPGPAGNVGAPGPKGARGSAGPPGATGFPGAAG**

**RVGPPGPSGNAGPPGPPGPAGKEGGKGPRGETGPIGRPGEVGPPGPPGPSGEKGSPGADG**

**PAGAPGTPGPQGIAGQRGVVGLPGQRGERGFPGLPGPSGEPGKQGPSGVSGERGPPGPAG**

**PPGLAGPPGESGREGSPGAEGSPGRDGAPGPKGDRGETGPAGPPGAPGAPGAPGPVGPAG**

**KAGDRGETGPSGPAGPAGPTGARGPAGPQGPRGDKGETGEQGDRGMKGHRGFSGLQGPPG**

**PPGSPGEQGPSGASGPAGPRGPPGSAGAAGKDGLNGLPGPIGPPGPRGRTGDAGPAGPPG**

**PPGPPGPPGPPSGGFDFSFLPQPPQEKAHDSGRYYRARQYDA-KGIDMGPGPMGLMGPRG**

**PPGASGPPGAQGFQGPAGEPGEPGQTGPAGARGPPGPPGKSGEDGHPGKPGRPGERGIVG**

**PQGARGFPGTPGLPGFKGIRGHNGLDGLKGQAGAPGVKGEPGAPGENGTPGQAGARGLPG**

**ERGRIGGPGPAGARGSDGSVGPVGPAXXXXXXGPPGFPGAPGPKGELGPVGNPGPAGPAG**

**PRGELGLPGMTGPVGPAGNPGANGLTGAKGAAGLPGVAGAPGLPGPRGIPGPAGAAGASG**

**PRGLAGEPGPAGSKGESGNKGEPGSAGPQGPPGPNGEEGKRGPNGEPGSTGPMGPPGLRG**

**VPGSRGLPGADGRAGGMGPPGNRGPSGPAGARGPNGDAGRPGEPGLMGPRGLPGSPGNVG**

**PTGKEGPAGLPGIDGRPGPTGPAGNRGEPGNIGFPGPKGPNGDPGKAGEKGHAGLAGARG**

**APGPDGNNGAQGPPGPTGVQGGKGEQGPAGPPGFQGLPGPSGPAGEGGKVGERGLPGEFG**

**LPGPAGPRGERGPPGESGAVGPTGSIGSRGPSGPPGPDGNKGEPGVVGAPGNAGPAGSGG**

**VPGERGAAGVPGGKGEKGETGPRGEFGNPGRDGARGAPGAMGAPGPAGATGERGEAGPAG**

**PVGPTGNRGAPGDRGEAGPAGPNGFAGPPGAAGQAGAKGERGTKGPKGENGIVGPTGPVG**

**AAGPAGPNGPPGPVGGRGDGGPPXXXXXXXXXXXXXXXXXXXXXGMTGFPGAAGKEGPRG**

**PRGDQGPLGRAGETGAVGPPGFAGEKGPPGEAGASGPPGSSGPQGLLGAPGILGLPGSRG**

**ERGLPGVSGSLGEPGPLGISGPPGARGPPGAVGSPGVNGAPGEAGRDGNPGNDGPPGRDG**

**LAGHKGERGYPGNPGAVGNAGAPGPHGTVGPAGKPGNRGEPGPVGSVGPVGPFGARGPSG**

**PQGPRGDKGEVGDKGPRGMNGFKGHNGFQGLPGLSGQHGDQGAPGSTGPAGPRGPAGPSG**

**PPGKDGRPGHAGAVGPAGIRGSQGSQGPAGPPGPPGLPGPPGPSGGGYDFGYEGDFYRA**
