# Supplementary material for: Ancient collagen reveals evolutionary history of the endemic South American ‘ungulates’
Source: Proc Biol Sci. 2015 May 7;282(1806):20142671. doi: 10.1098/rspb.2014.2671 (PMC4426609; doi:10.1098/rspb.2014.2671)
Supplement: ESM5 [file rspb20142671supp5.zip › ESM5.pdf]

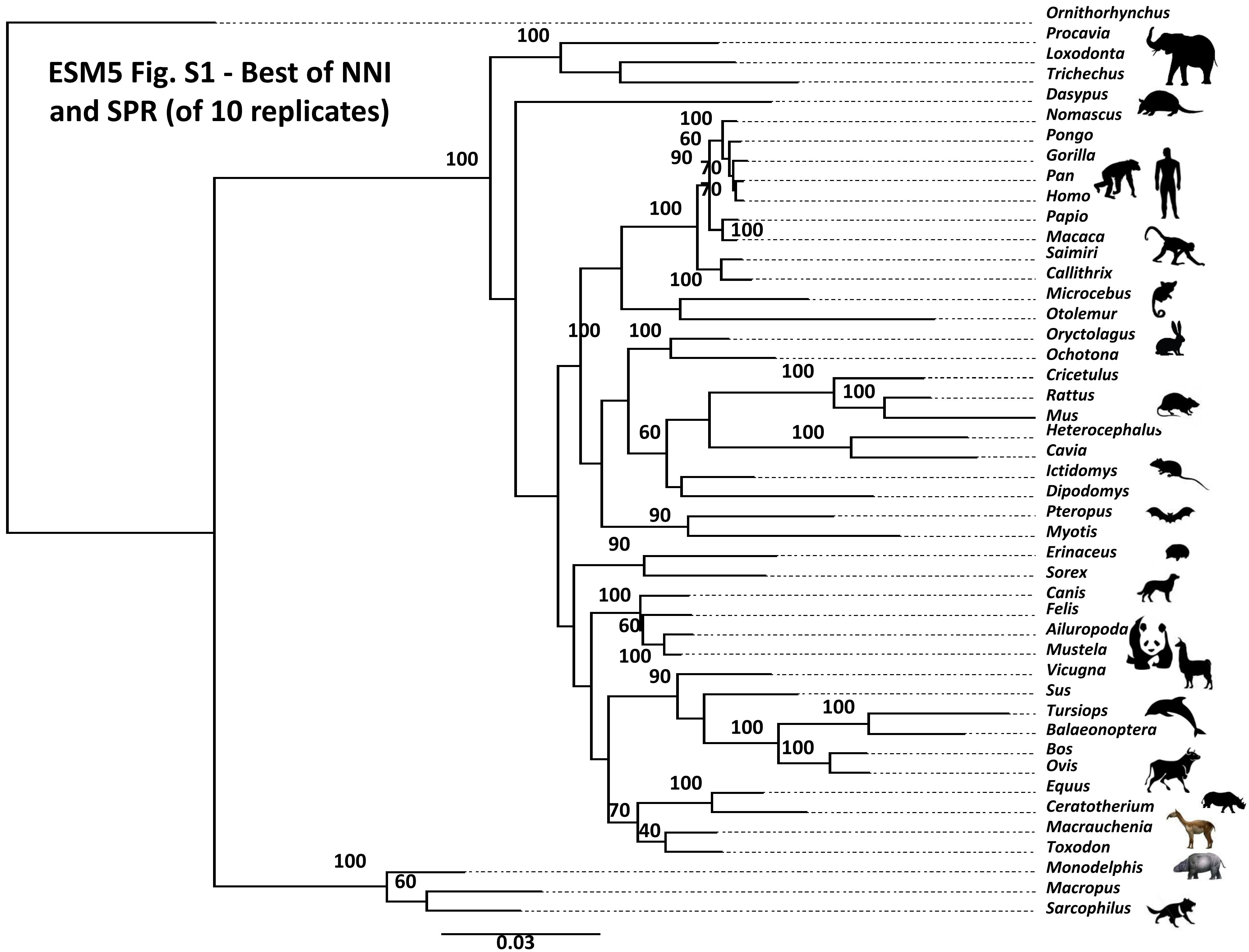
ESM5 Fig. S1 - Best of NNI and SPR (of 10 replicates)
Ornithorhynchus
Procavia
Loxodonta
Trichechus
Dasypus
Nomascus
Pongo
Gorilla
Pan
Homo
Papio
Macaca
Saimiri
Callithrix
Microcebus
Otolemur
Oryctolagus
Ochotona
Cricetulus
Rattus
Mus
Heterocephalus
Cavia
Ictidomys
Dipodomys
Pteropus
Myotis
Erinaceus
Sorex
Canis
Felis
Ailuropoda
Mustela
Vicugna
Sus
Tursiops
Balaeonoptera
Bos
Ovis
Equus
Ceratotherium
Macrauchenia
Toxodon
Monodelphis
Macropus
Sarcophilus
0.03

# ESM5 Fig. S2 - Maximum Likelihood ‘pseudoextinction’ analysis

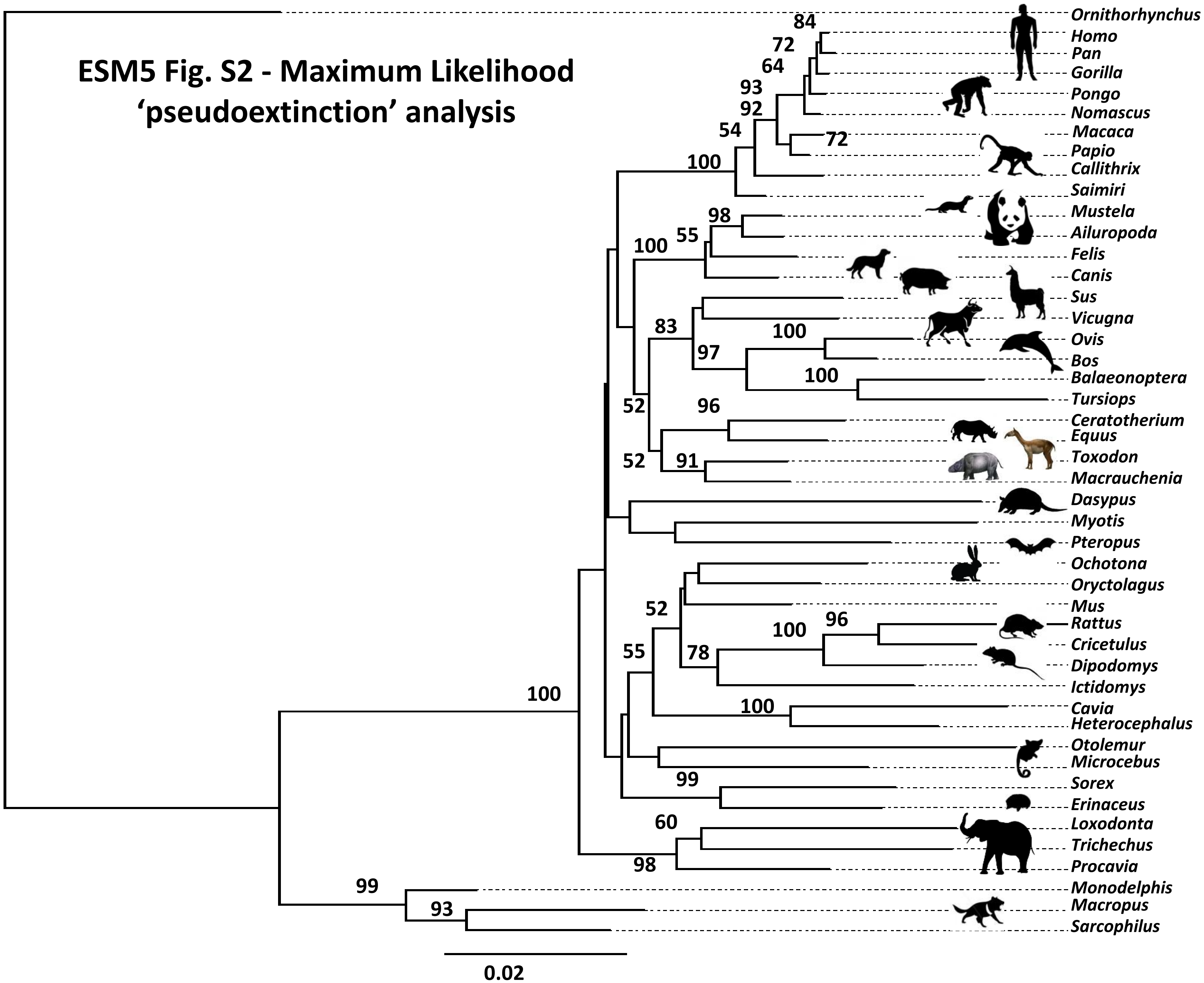

# ESM5 Fig. S3 - Maximum Likelihood PMF analysis

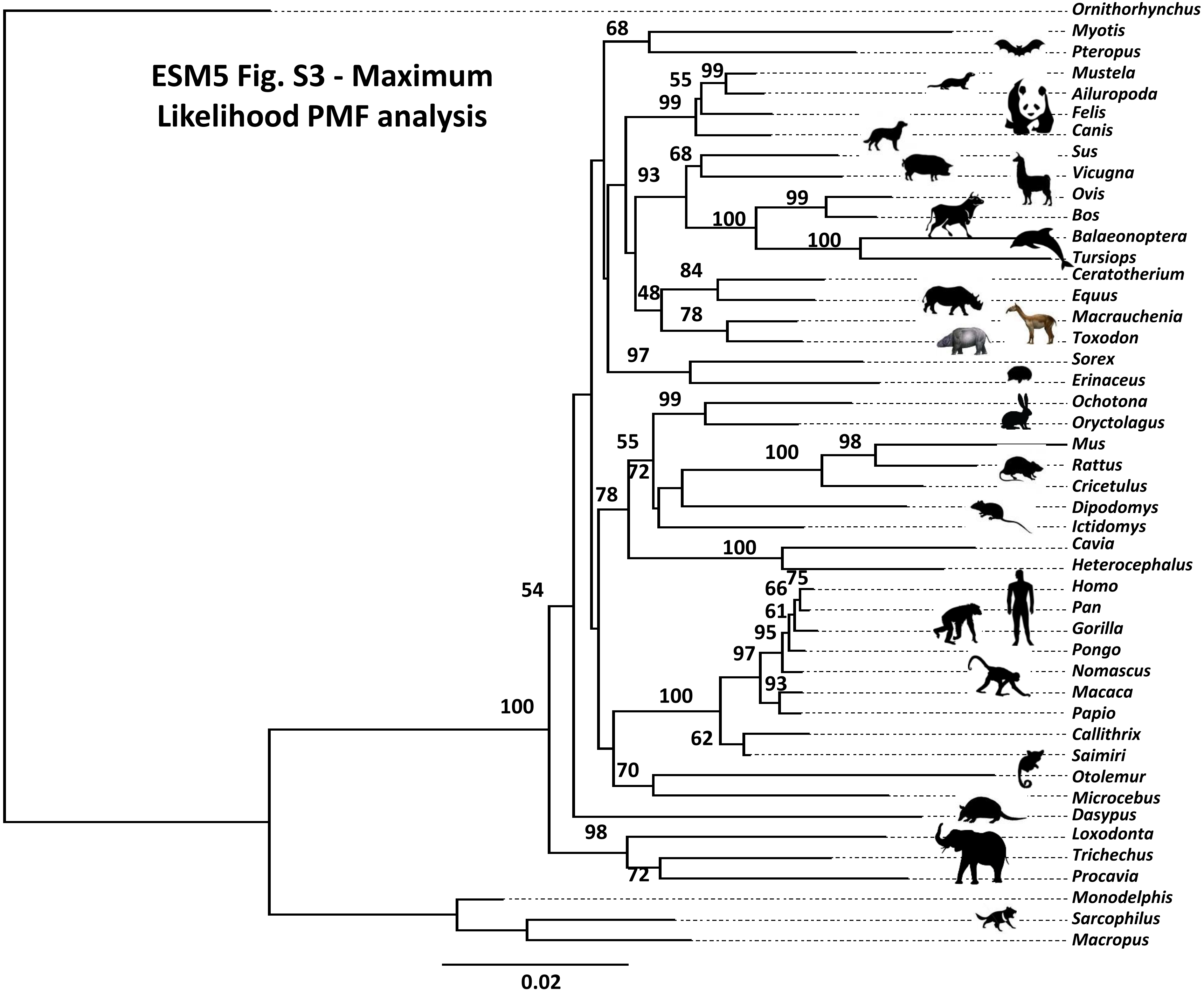

# ESM5 Fig. S4 – Parsimony analysis

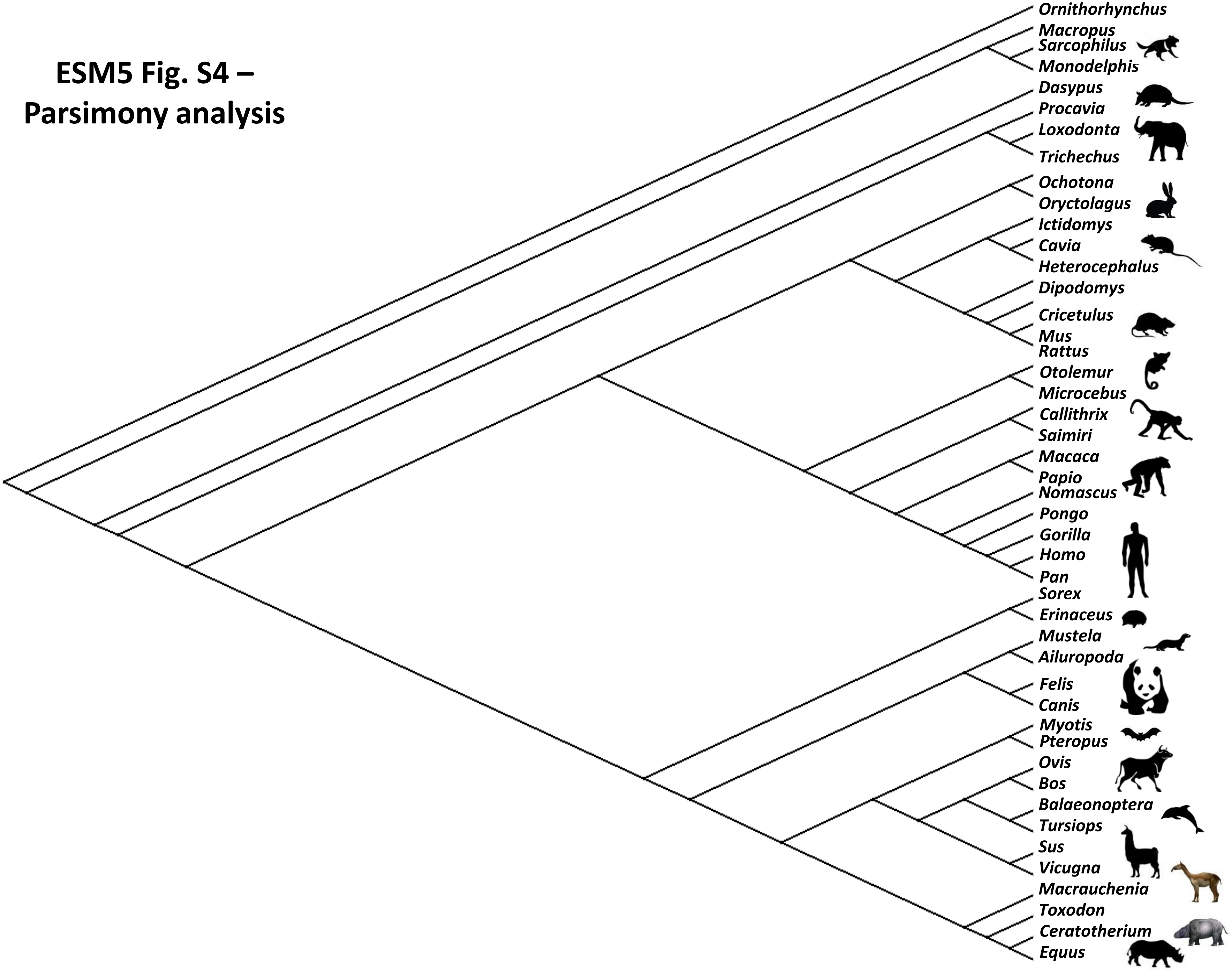

ESM5 Fig. S5 – Bayesian analysis (see Fig. S6 for trace)

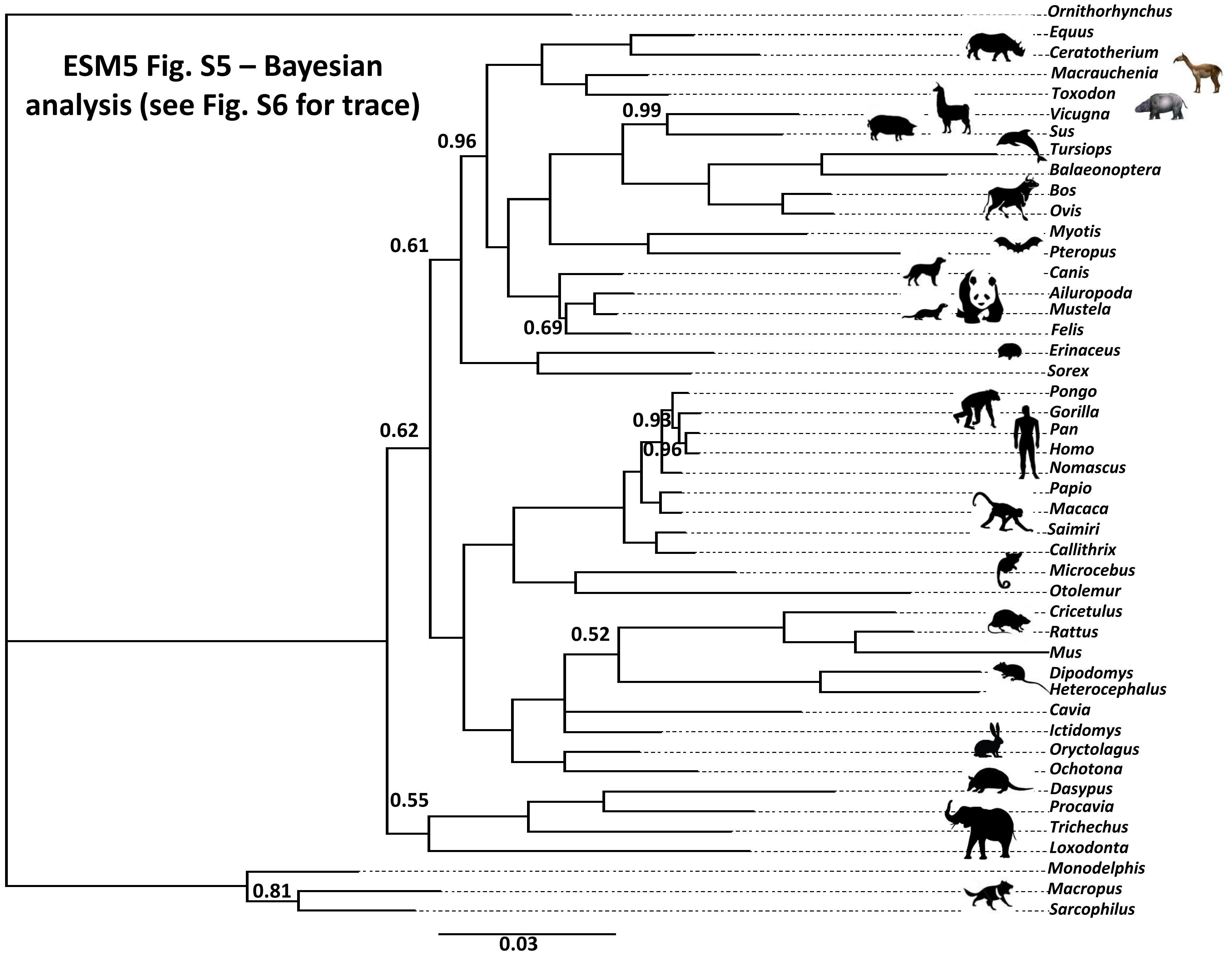

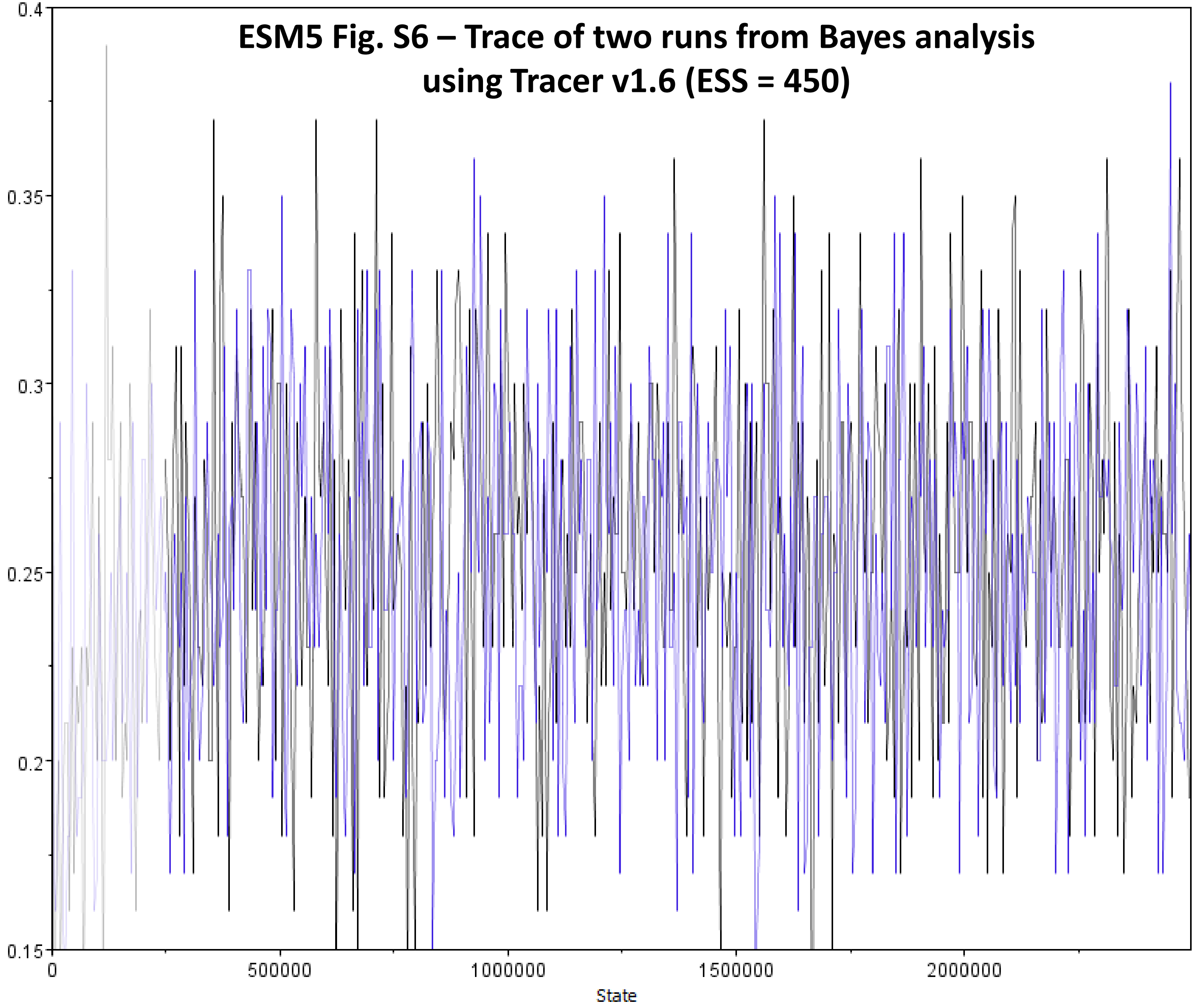


**ESM5 Fig. S6 – Trace of two runs from Bayes analysis using Tracer v1.6 (ESS = 450)**
