# Supplementary material for: Ancient collagen reveals evolutionary history of the endemic South American ‘ungulates’
Source: Proc Biol Sci. 2015 May 7;282(1806):20142671. doi: 10.1098/rspb.2014.2671 (PMC4426609; doi:10.1098/rspb.2014.2671)

**ESM5 Fig. S1 - Best of NNI and SPR (of 100 replicates; only scores >50 shown)**

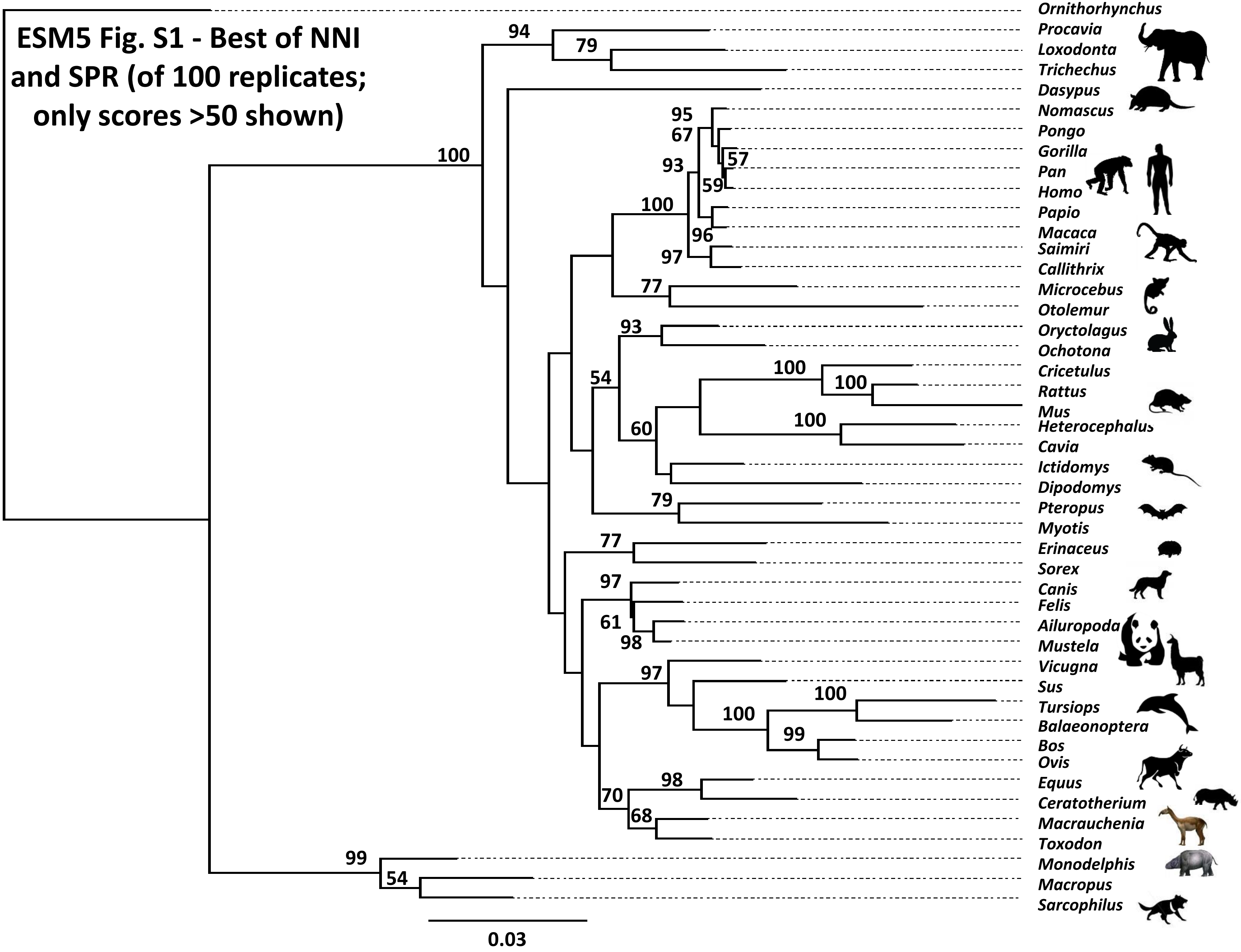

# ESM5 Fig. S2 - Maximum Likelihood 'pseudoextinction' analysis with NNI

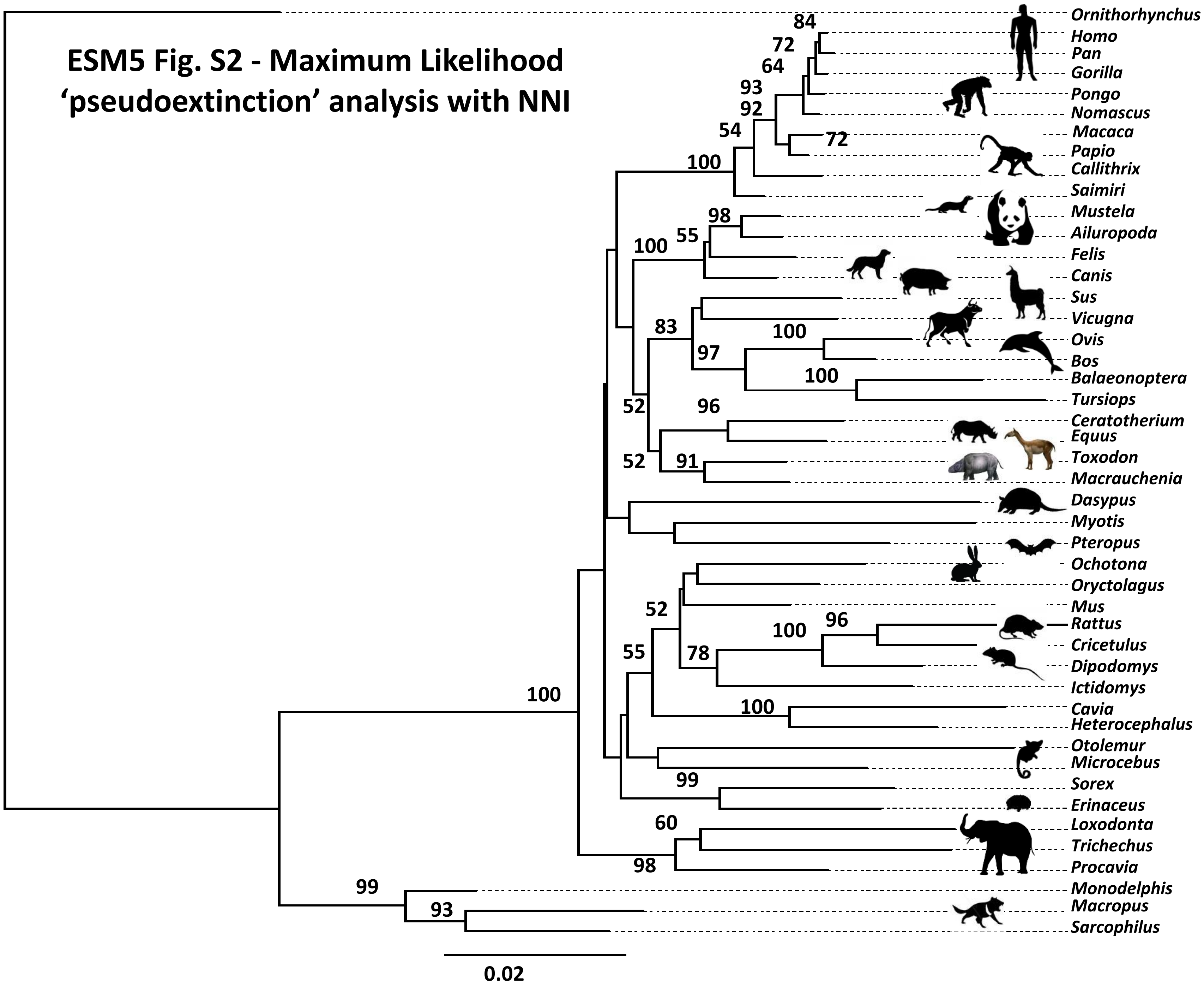

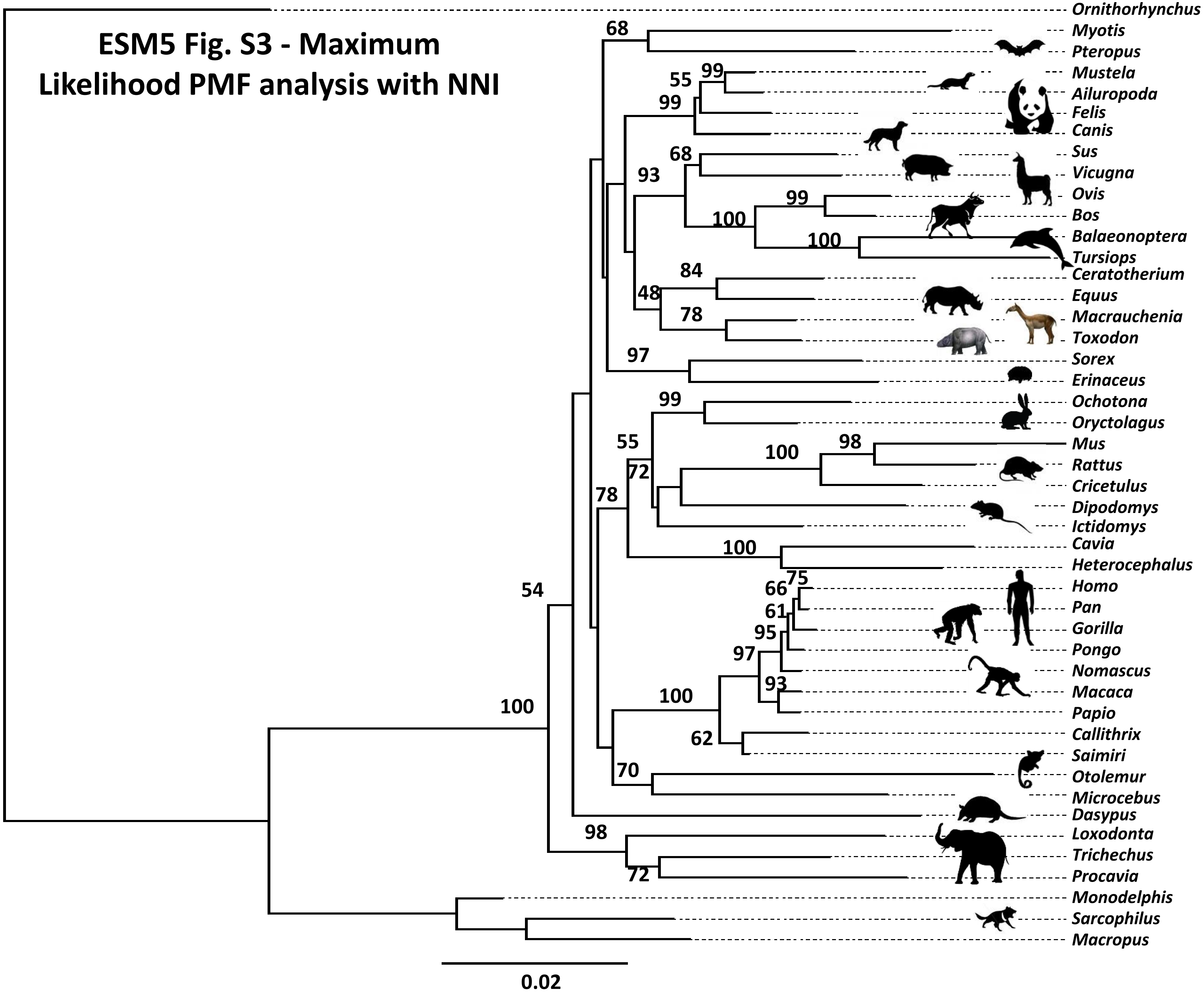

ESM5 Fig. S3 - Maximum Likelihood PMF analysis with NNI
Ornithorhynchus
Myotis
Pteropus
Mustela
Ailuropoda
Felis
Canis
Sus
Vicugna
Ovis
Bos
Balaenoptera
Tursiops
Ceratotherium
Equus
Macrauchenia
Toxodon
Sorex
Erinaceus
Ochotona
Oryctolagus
Mus
Rattus
Cricetulus
Dipodomys
Ictidomys
Cavia
Heterocephalus
Homo
Pan
Gorilla
Pongo
Nomascus
Macaca
Papio
Callithrix
Saimiri
Otolemur
Microcebus
Dasypus
Loxodonta
Trichechus
Procavia
Monodelphis
Sarcophilus
Macropus
68
55
99
99
68
93
99
100
100
84
48
78
97
99
55
72
78
100
98
100
66
75
61
95
97
93
100
62
70
54
100
98
72
0.02

# ESM5 Fig. S4 – Parsimony analysis

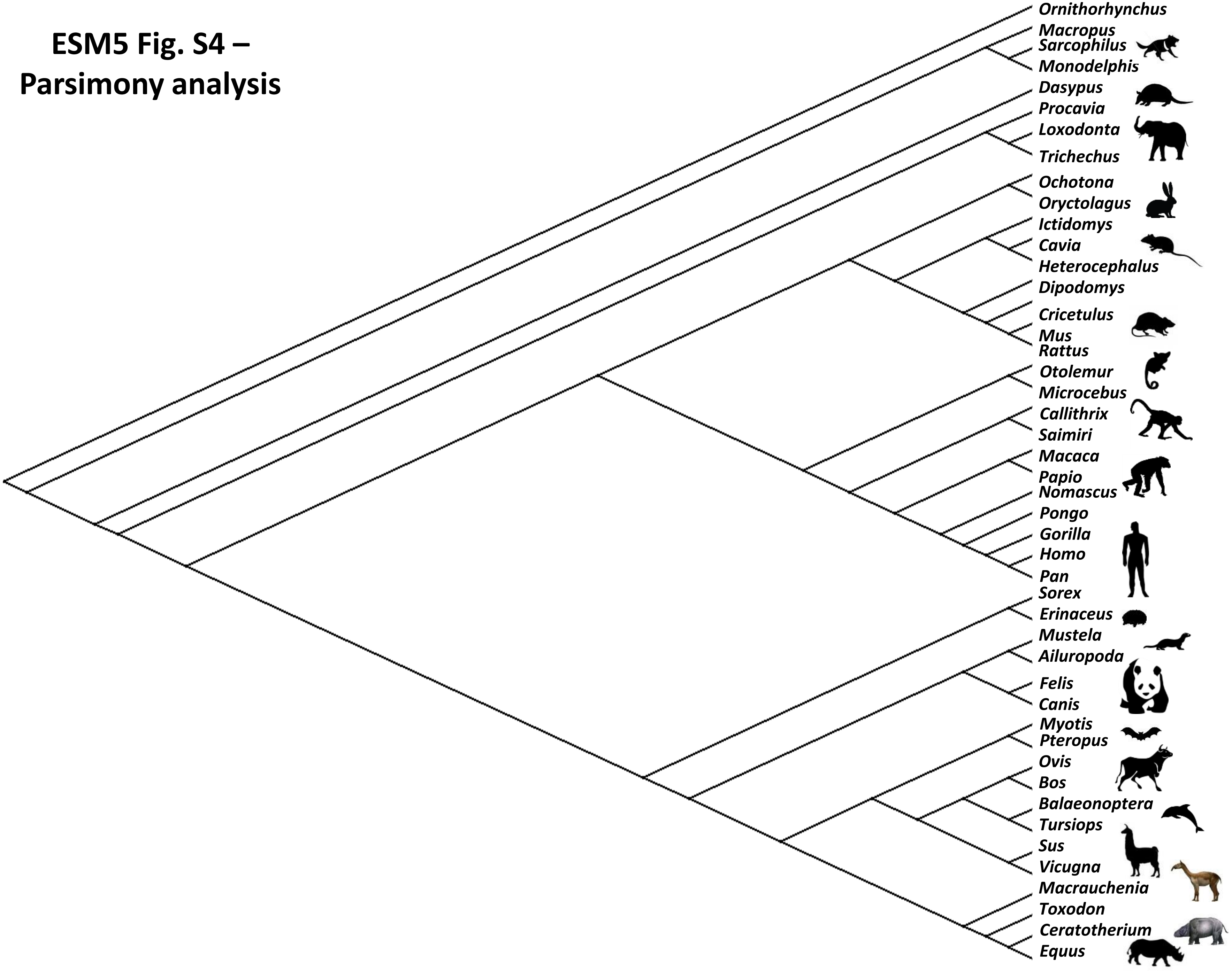

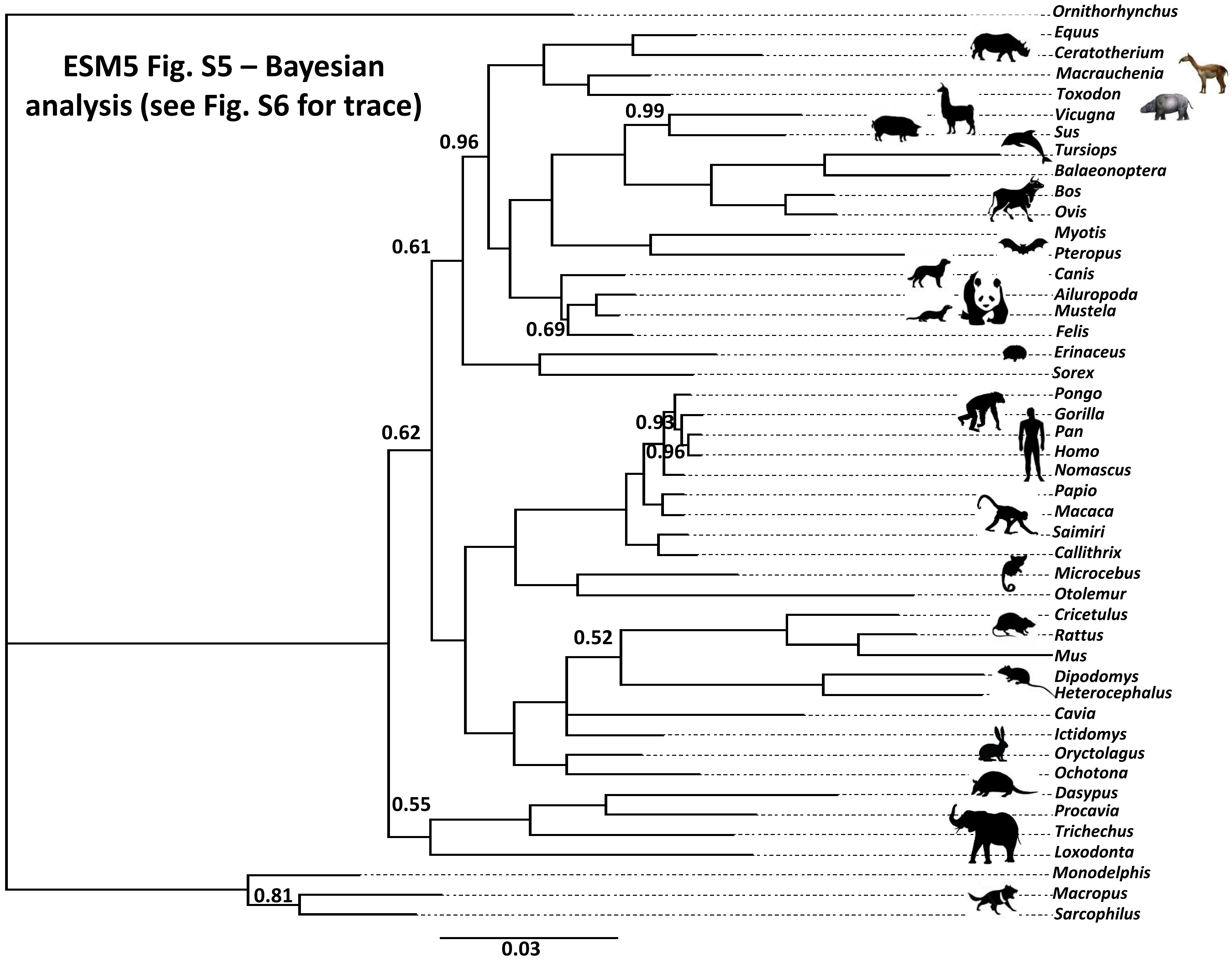

ESM5 Fig. S5 – Bayesian analysis (see Fig. S6 for trace)
Ornithorhynchus
Equus
Ceratotherium
Macrauchenia
Toxodon
Vicugna
Sus
Tursiops
Balaeonoptera
Bos
Ovis
Myotis
Pteropus
Canis
Ailuropoda
Mustela
Felis
Erinaceus
Sorex
Pongo
Gorilla
Pan
Homo
Nomascus
Papio
Macaca
Saimiri
Callithrix
Microcebus
Otolemur
Cricetulus
Rattus
Mus
Dipodomys
Heterocephalus
Cavia
Ictidomys
Oryctolagus
Ochotona
Dasypus
Procavia
Trichechus
Loxodonta
Monodelphis
Macropus
Sarcophilus
0.99
0.96
0.61
0.69
0.93
0.96
0.62
0.52
0.55
0.81
0.03

**ESM5 Fig. S6 – Trace of two runs from Bayes analysis using Tracer v1.6 (ESS = 450)**

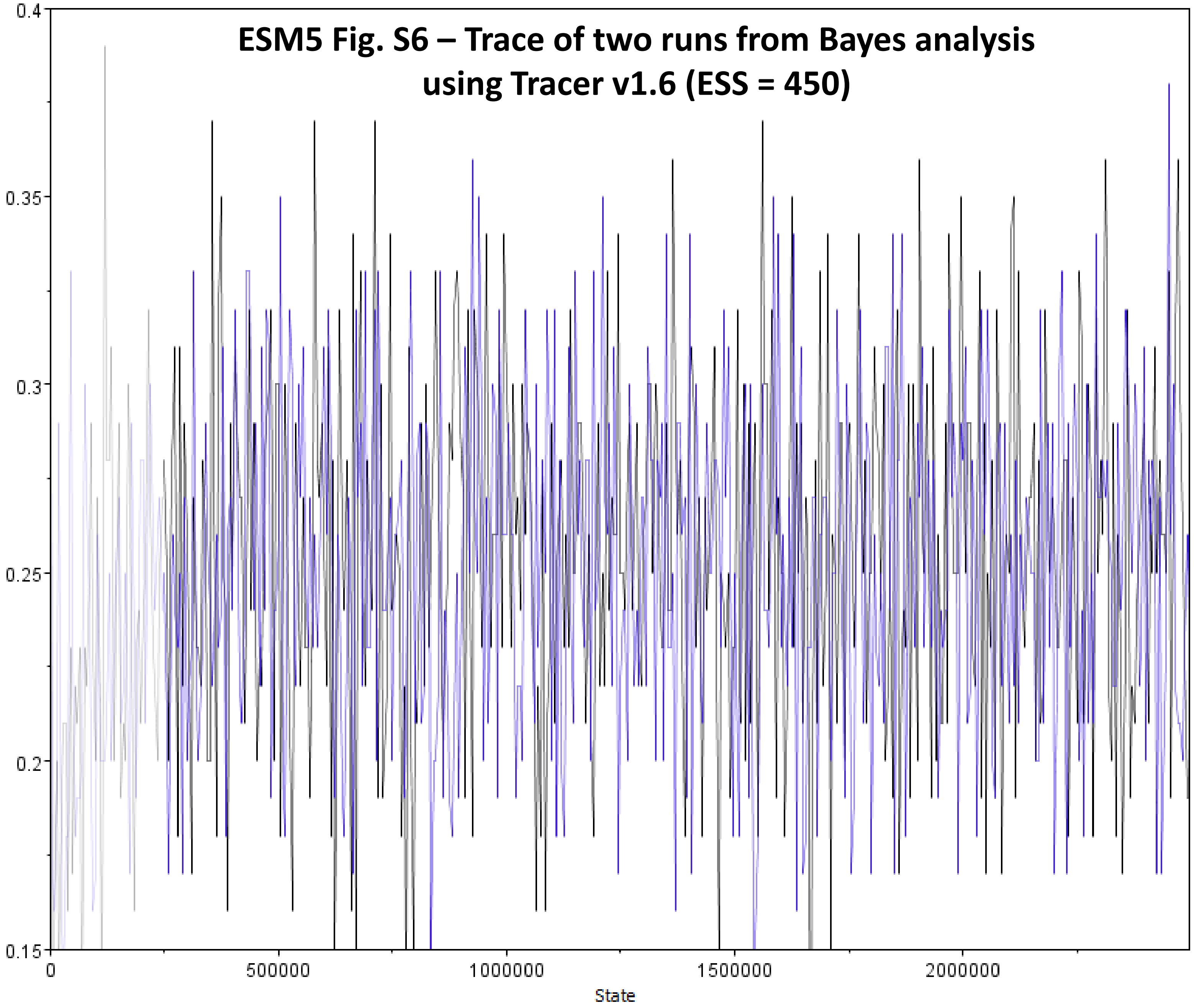

Supplement: ESM5 [file rspb20142671supp5.zip › ESM5revised.pdf]
